# Isogenic Sets of hiPSC-CMs Harboring Distinct *KCNH2* Mutations Differ Functionally and in Susceptibility to Drug-Induced Arrhythmias

**DOI:** 10.1016/j.stemcr.2020.10.005

**Published:** 2020-11-10

**Authors:** Karina O. Brandão, Lettine van den Brink, Duncan C. Miller, Catarina Grandela, Berend J. van Meer, Mervyn P.H. Mol, Tessa de Korte, Leon G.J. Tertoolen, Christine L. Mummery, Luca Sala, Arie O. Verkerk, Richard P. Davis

**Affiliations:** 1 Department of Anatomy and Embryology, Leiden University Medical Center, 2300RC Leiden, The Netherlands; 2 Istituto Auxologico Italiano, IRCCS, Laboratory of Cardiovascular Genetics, 20095 Milan, Italy; 3 Department of Medical Biology, Amsterdam UMC, University of Amsterdam, 1105AZ Amsterdam, The Netherlands; 4 Department of Experimental Cardiology, Amsterdam UMC, University of Amsterdam, 1105AZ Amsterdam, The Netherlands

**Keywords:** long QT syndrome 2, disease modeling, induced pluripotent stem cells, isogenic, arrhythmia, risk stratification, genome editing, electrophysiology

## Abstract

Mutations in *KCNH2* can lead to long QT syndrome type 2. Variable disease manifestation observed with this channelopathy is associated with the location and type of mutation within the protein, complicating efforts to predict patient risk. Here, we demonstrated phenotypic differences in cardiomyocytes derived from isogenic human induced pluripotent stem cells (hiPSC-CMs) genetically edited to harbor mutations either within the pore or tail region of the ion channel. Electrophysiological analysis confirmed that the mutations prolonged repolarization of the hiPSC-CMs, with differences between the mutations evident in monolayer cultures. Blocking the hERG channel revealed that the pore-loop mutation conferred greater susceptibility to arrhythmic events. These findings showed that subtle phenotypic differences related to *KCNH2* mutations could be captured by hiPSC-CMs under genetically matched conditions. Moreover, the results support hiPSC-CMs as strong candidates for evaluating the underlying severity of individual *KCNH2* mutations in humans, which could facilitate patient risk stratification.

## Highlights

• Mutation-specific differences detected in hiPSC-CMs with same genetic background
• APD and FPD in the hERG pore variant hiPSC-CMs more prolonged than the tail variant
• The pore variant was also more susceptible to drug-induced arrhythmic events
• Potential strategy to determine *KCNH2* mutation-specific arrhythmic risk

## Graphical Abstract

**Figure:**
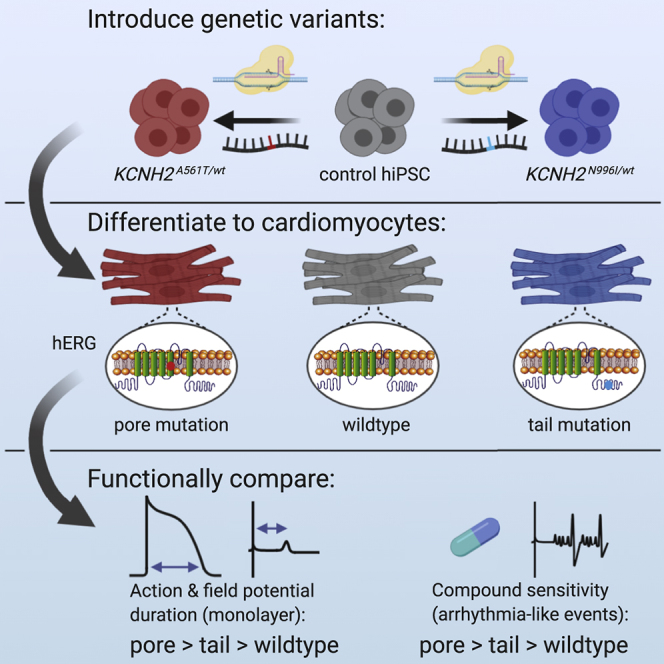

## Teaser

In this article, Davis and colleagues showed that, by studying on the same genetic background different *KCNH2* mutations that can cause LQT2, subtle molecular and electrophysiological differences related to the specific mutation were captured by the corresponding hiPSC-CMs; raising the possibility of using hiPSC-CMs to model mutation-location differences observed in LQT2 patients and thereby assist in patient risk stratification.

## Introduction

Congenital long QT syndrome (LQTS) is a genetic disease with an estimated prevalence of ∼1:2,000 individuals. It is characterized by a prolonged QT interval on an electrocardiogram that can lead to sudden cardiac death, particularly in young people (George, 2013). Although the identification of genes associated with LQTS has dramatically improved our understanding of the disease, clinical management remains complicated by the variability in disease expressivity and penetrance among mutation carriers which range from lifelong asymptomatic to experiencing life-threatening arrhythmias (Giudicessi and Ackerman, 2013). While environmental factors are contributors to this clinical heterogeneity (Bezzina et al., 2015), genetics also plays a major role through both the primary genetic mutation and the presence of additional genetic variants that modify the disease outcome (Napolitano et al., 2018; Schwartz et al., 2018).

Type 2 LQTS (LQT2) is the second most prevalent form of congenital LQTS and is due to mutations in *KCNH2*, which encodes the α subunit of the Kv11.1 (hERG) channel responsible for conducting the rapid delayed rectifier potassium current (I_Kr_) in cardiomyocytes (Curran et al., 1995). Several studies have demonstrated that the location of the mutation within this ion channel is an important determinant of arrhythmic risk in LQT2 patients, with patients harboring mutations in the pore-loop region at higher risk of cardiac events than those with mutations located in other regions (Migdalovich et al., 2011; Moss et al., 2002; Nagaoka et al., 2008; Shimizu et al., 2009). Furthermore, mutations that result in a dominant-negative effect, in which the function of wild-type hERG is reduced or eliminated, also produce higher adverse event rates (Migdalovich et al., 2011). However, mutations within the pore-loop region can, in some instances, result in less severe outcomes (Zhao et al., 2009), highlighting the need for *in vitro* models to accurately classify these rare variants and for gaining mechanistic insights into their contribution to disease phenotypes.

Cardiomyocytes derived from human induced pluripotent stem cells (hiPSCs) are now well established as models for LQT2 (Bellin et al., 2013; Itzhaki et al., 2011; Matsa et al., 2011; Mehta et al., 2018). Indeed, a number of hiPSC lines have been derived from both symptomatic and asymptomatic patients with mutations in various regions of hERG (Brandão et al., 2017). However, as these lines are from different individuals, they harbor additional genetic variants that may functionally influence the disease phenotype observed and limit the utility of hiPSC-derived cardiomyocytes (hiPSC-CMs) for broad intragenotype risk stratification.

To create a tailored model to study the genetic etiology of LQT2, we generated a set of isogenic hiPSC lines that possess heterozygous mutations within the pore-loop domain (KCNH2-A561T) or in the cytoplasmic tail (KCNH2-N996I) of hERG by genetically modifying a control hiPSC line. Molecular and functional comparisons of these edited lines confirmed not only that the *KCNH2* variant hiPSC-CMs phenocopied the key features of LQT2 but also that differences due to the mutation were identified in the cell lines. This included dissimilarities in the mechanism underlying the hERG channel defect caused by these mutations, as well as a more prolonged repolarization observed in hiPSC-CMs with the pore mutation when measured as a syncytium. Furthermore, when these hiPSC-CMs were exposed to E-4031, a hERG channel blocker, they were more susceptible to proarrhythmic effects compared with either the hiPSC-CMs with the tail mutation or the unedited control. Our findings highlight the potential of hiPSC-CMs to reveal the inherent severity of individual *KCNH2* mutations when using genetically matched lines, and also further advance hiPSC-CMs as models for not only predicting risk but also assisting in the stratification of patients.

## Results

### Generation and Characterization of an Isogenic Set of KCNH2 Variant hiPSC Lines

A limitation of hiPSC lines derived from unrelated patients with different LQT2-causing mutations is the inability to compare the resulting hiPSC-CMs under genetically matched conditions. Therefore, to detect phenotypic differences between individual LQT2-causing *KCNH2* mutations, we elected to genetically introduce these variants into a well-characterized hiPSC line derived from a healthy individual (KCNH2^WT/WT^) (Zhang et al., 2014). Furthermore, we confirmed that this cell line did not carry any known disease-causing mutations by performing whole-exome sequencing and examining a panel of 107 genes known to be linked to inherited arrhythmia syndromes or cardiomyopathies (Pua et al., 2016) (Table S1). All coding sequence variants identified were predicted to be benign due to their frequency in the general population being ≥1%. The only exception was a rare variant identified in *DOLK*, the gene encoding dolichol kinase. Homozygous mutations in this gene can lead to multi-systemic glycosylation disorders, including dilated cardiomyopathy, with individuals typically not surviving to adulthood (Lefeber et al., 2011). However, the variant identified in the KCNH2^WT/WT^ line is unlikely to be pathogenic as the hiPSCs were derived from a healthy 51-year-old female and were heterozygous for the *DOLK* variant.

**Figure 1:**
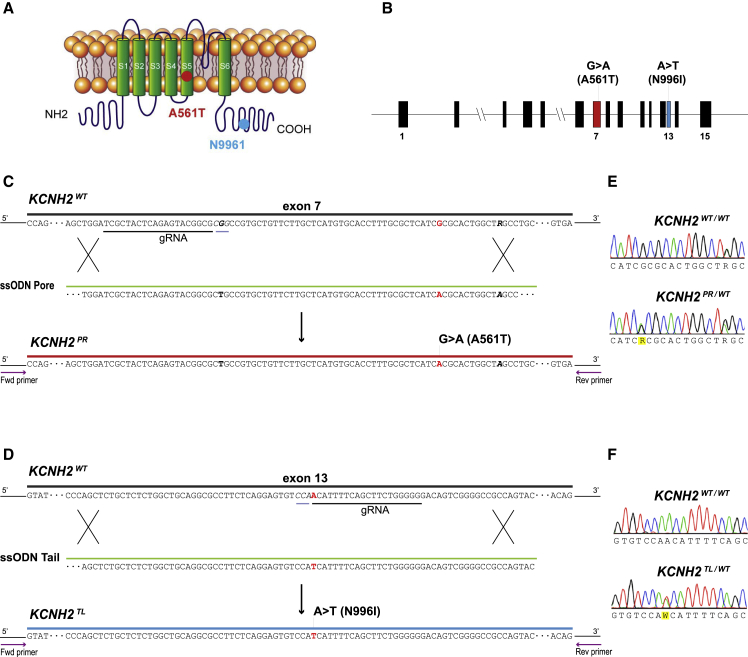
Generation of Isogenic hiPSC Lines with *KCNH2* Mutations (A) Structure of the potassium ion channel hERG encoded by *KCNH2* indicating the introduced mutations (KCNH2-A561T, red dot; KCNH2-N996I, blue dot). (B) Structure of the *KCNH2* genomic locus highlighting the exons modified to generate the KCNH2 variant hiPSC lines. (C and D) Schematic outlining strategy to introduce the KCNH2-A561T mutation (*KCNH2*^*PR*^) (C) or the KCNH2-N996I mutation (*KCNH2*^*TL*^) (D) by homologous recombination into a *KCNH2* wild-type (*KCNH2*^*WT*^) sequence. The gRNA and their corresponding protospacer adjacent motif sequences are underlined in black and gray, respectively. Part of the ssODN sequences to introduce the A561T (ssODN Pore) and N996I (ssODN Tail) mutations are shown. Nucleotides modified to introduce the mutations are indicated in red, silent mutations and SNPs used to assist with the targeting and screening are bolded in black. Arrows represent the PCR primers used to identify targeted clones. (E and F) Sequence analysis of the PCR-amplified genomic DNA showing heterozygous introduction of NM_000238.3:c.G1681A (E) and NM_000238.3:c.A2987T (F). See also Figure S1.

We then used a CRISPR-Cas9-mediated gene editing strategy to generate an isogenic set of hiPSC lines harboring a missense variant either within the pore-loop domain (NM_000238.3:c.1681G > A, NP_000229.1:p.Ala561Thr) or cytoplasmic tail region (NM_000238.3:c.2987A > T, NP_000229.1:p.Asp996Iso) of *KCNH2* (Figure 1). Restriction fragment length polymorphism analysis identified clones that appeared to be genetically modified and these were subsequently confirmed by Sanger sequencing to be either heterozygous for the KCNH2-A561T (KCNH2^PR/WT^; Figure 1E) or the KCNH2-N996I (KCNH2^TL/WT^; Figure 1F) variant. For each mutation, a second independent isogenic heterozygous clone (KCNH2^PR/WT^ cl2 and KCNH2^TL/WT^ cl2) was also selected for further characterization (Figures S1A–S1C). All clones were assessed by Sanger sequencing for potential off-target modifications due to the CRISPR-Cas9 transfection, with no insertions or deletions detected at any of the genomic loci examined (Table S2). Furthermore, G-band karyotyping of these clones indicated that the lines were karyotypically normal, and the undifferentiated hPSCs expressed the stem cell markers SOX2, OCT-4, NANOG, SSEA4, and TRA-1-60 (Figures S1D–S1F).

### Distinct hERG Mutations Result in Differing Channel Functionality

**Figure 2:**
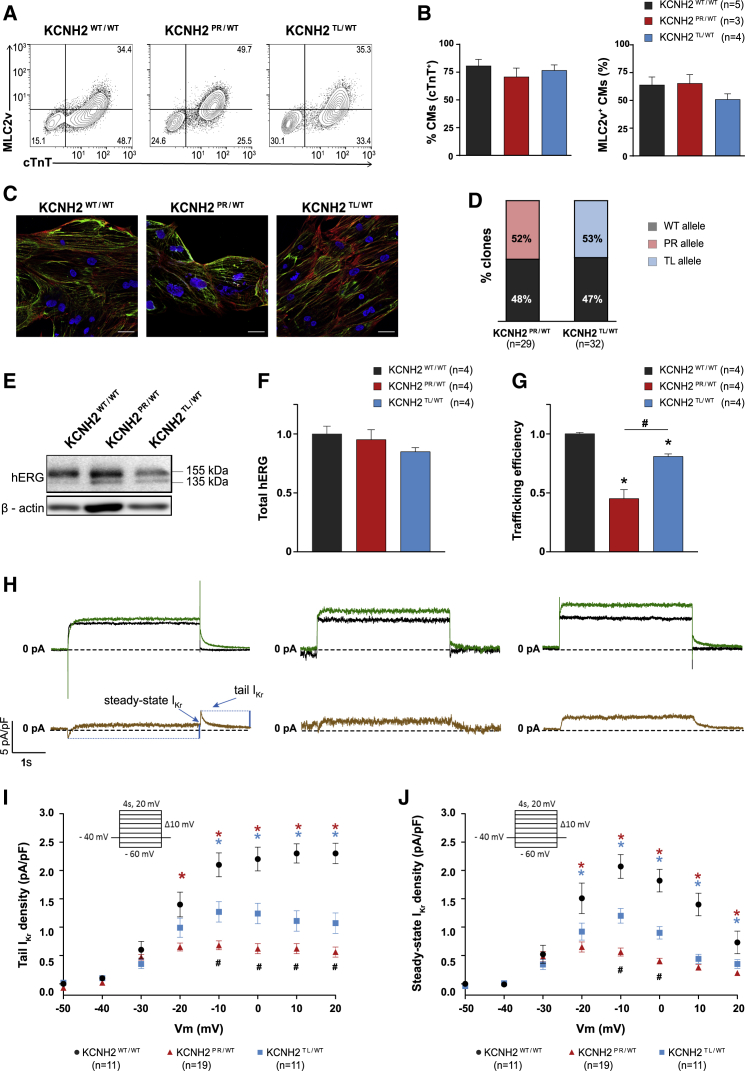
Evaluation of hERG Channel Function in Differentiation Day 21 + 7 KCNH2^WT/WT^, KCNH2^PR/WT^, and KCNH2^TL/WT^ hiPSC-CMs (A) Representative flow cytometry plots of hiPSC-CMs for expression of cTnT and MLC2v in the indicated lines. Values inside the plots are the percentage of cells within the gated region. (B) Overall cardiac differentiation efficiency of the three hiPSC lines, showing the average percentage of hiPSC-CMs (cTnT^+^) (left graph), and the proportion of ventricular-like (MLC2v^+^) cardiomyocytes within the hiPSC-CM population (right graph). Values (n) refer to the number of independent differentiations analyzed. (C) Immunofluorescence images of the cardiac sarcomeric proteins α-actinin (red) and myosin heavy chain isoforms α and β (green) in the indicated lines. Nuclei (blue) were stained with DAPI. Scale bars, 25 μm. (D) Percentage of *KCNH2* mRNA in the KCNH2^PR/WT^ and KCNH2^TL/WT^ hiPSC-CMs transcribed from the wild-type (WT) or mutated (PR, TL) alleles. Values (n) refer to the number of independent clones sequenced. (E) Western blot analysis of hERG in the indicated lines. Bands corresponding to core and fully glycosylated hERG (135 and 155 kDa, respectively) are marked. β-Actin was used as a loading control. (F and G) Densitometric quantification of western blots in (E) and Figure S2C for total hERG protein (155 +135 kDa; relative to KCNH2^WT/WT^) (F), and the ratio of fully glycosylated over total hERG protein (trafficking efficiency) (G). Data normalized to β-actin. ^∗^statistical significance to KCNH2^WT/WT^ (p < 0.05); #statistical significance between KCNH2^PR/WT^ and KCNH2^TL/WT^ (p < 0.001); one-way ANOVA with Tukey's multiple comparisons test. Values (n) refer to the number of independent differentiations analyzed. (H) Top panels show representative current traces evoked by a 4-s voltage step from −40 to 0 mV before (green lines) and after (black lines) application of 5 μM E-4031. Bottom panels present the E-4031-sensitive current (brown lines). Arrows indicate the sections of the traces analyzed to determine the tail and steady-state I_Kr_ current densities. (I and J) Average current-voltage relationships for tail (I) and steady-state (J) I_Kr_ current densities in the indicated hiPSC-CMs. All CMs activated upon depolarization reached a maximum steady-state current at −10 and 0 mV, which decreased at more positive potentials due to the onset of inactivation. Inset: voltage protocol; ^∗^statistical significance to KCNH2^WT/WT^ (tail I_Kr_, p < 0.0001; steady-state I_Kr_, p < 0.01); #statistical significance between KCNH2^PR/WT^ and KCNH2^TL/WT^ (tail I_Kr_: −10 mV, 0 mV, p < 0.001; 10 mV, 20 mV, p < 0.01; steady-state I_Kr_: −10 mV, p < 0.0001, 0 mV, p < 0.01); two-way ANOVA with Tukey's multiple comparisons test. Values (n) refer to the number of individual hiPSC-CMs analyzed. Error bars represent SEM. See also Figure S2.

A monolayer-based differentiation protocol was used to differentiate the KCNH2 variant and KCNH2^WT/WT^ hiPSC lines to cardiomyocytes. Flow cytometric analysis for the pan-cardiomyocyte marker cardiac troponin T (cTnT) indicated that all lines, except KCNH2^PR/WT^ cl2, differentiated with similar efficiencies, with on average ∼70%–80% of the cells being cTnT^+^ (Figures 2A, 2B, S2A, and S2B). The hiPSC-CMs (cTnT^+^ cells) were also characterized for the expression of the ventricular cardiomyocyte marker, MLC2v. On average, the proportion of hiPSC-CMs that were ventricular was between 45% and 64% including for the KCNH2^PR/WT^ cl2 line (Figures 2A, 2B, S2A, and S2B). The hiPSC-CMs also displayed characteristic sarcomeric structures that were positive for α-actinin and myosin heavy chain (Figure 2C).

Both KCNH2^PR/WT^ and KCNH2^TL/WT^ hiPSC-CMs expressed equal fractions of mutant and wild-type *KCNH2* transcripts (Figure 2D), confirming that the introduced mutations did not disrupt biallelic expression of the gene and suggesting that any differences observed between the cell lines would be due to dysfunction of hERG. Western blot analysis (Figure 2E) for hERG identified two protein bands—the fully glycosylated mature form (155 kDa) and the core-glycosylated precursor form (135 kDa) (Zhou et al., 1998a, 1998b). Although there was no significant difference in the total amount of hERG present between the three lines (Figure 2F), the mature form (155 kDa) was predominantly present in KCNH2^WT/WT^ hiPSC-CMs, while in both KCNH2^PR/WT^ and KCNH2^TL/WT^ hiPSC-CMs, higher expression of the precursor form (135 kDa) was detected (Figures S2C and S2D). This is in agreement with previous studies that have reported these mutations as affecting glycosylation of hERG, leading to impaired protein trafficking (Bellin et al., 2013; Matsa et al., 2014). hERG-trafficking efficiency, calculated as the ratio of fully glycosylated hERG over total hERG present (Peterson et al., 2012), indicated a significant reduction in both KCNH2 variant lines relative to the KCNH2^WT/WT^ line (KCNH2^PR/WT^: 55%; KCNH2^TL/WT^: 19%; Figure 2G).

Finally, we determined the effect of these *KCNH2* mutations on I_Kr_. Representative examples of current traces in individual hiPSC-CMs from both KCNH2 variant and KCNH2^WT/WT^ hiPSC-CMs are shown in Figure 2H, with I_Kr_ measured as an E-4031-sensitive current. The I_Kr_ tail and steady-state current densities were significantly lower in both KCNH2^PR/WT^ and KCNH2^TL/WT^ hiPSC-CMs compared with the KCNH2^WT/WT^ hiPSC-CMs (Figures 2I and 2J). For example, after a voltage step to −10 mV, the I_Kr_ tail current density was 39% smaller in the KCNH2^TL/WT^ hiPSC-CMs and 67% smaller in the KCNH2^PR/WT^ hiPSC-CMs. A similar fold reduction was also observed for the steady-state current at −10 mV (KCNH2^PR/WT^: 73%; KCNH2^TL/WT^: 42%). Taken together, these findings indicate that both *KCNH2* variants result in impaired glycosylation of the hERG protein but, in terms of the availability of functional channels, the KCNH2^TL/WT^ mutation leads to haploinsufficiency, while the KCNH2^PR/WT^ mutation causes a dominant-negative phenotype.

We also endeavored to demonstrate that reduced trafficking of hERG might contribute to the decrease in I_Kr_ density. To investigate this, we treated the hiPSC-CMs with lumacaftor, a drug shown previously to rescue hERG-trafficking defects and lead to a shortening of the field potential duration (FPD) (Mehta et al., 2018). While we also observed a significant shortening of the FPD in the KCNH2 variant lines upon treatment with 5 μM lumacaftor for 8 days (Figure S2E), a similar effect with the vehicle control (0.1% DMSO) was detected. DMSO is known to be able to stabilize protein conformation during their maturation and rescue trafficking defects (Thomas, 2003). Therefore, it is possible that the chaperone properties of this chemical as well as lumacaftor contributed to the FPD reduction we observed.

### The Dominant-Negative *KCNH2* Mutation Causes a More Severe Electrical Phenotype

**Figure 3:**
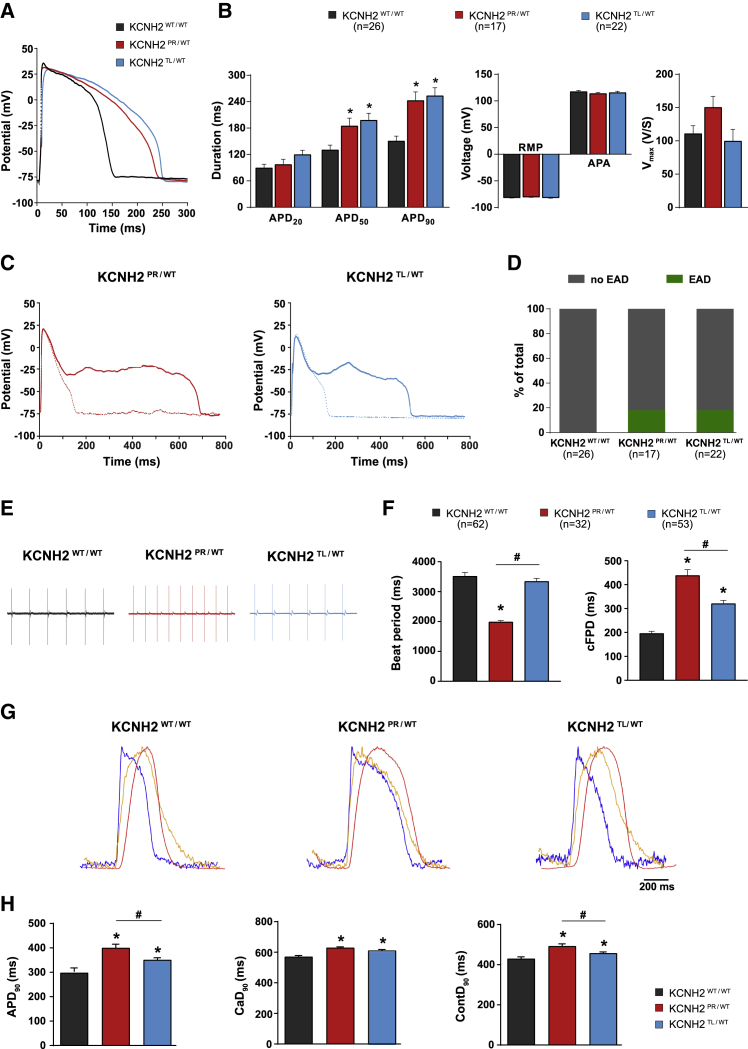
Electrophysiological Characterization of the KCNH2^WT/WT^, KCNH2^PR/WT^, and KCNH2^TL/WT^ hiPSC-CMs (A and B) Representative AP traces (A), and average APD_20_, APD_50_, APD_90_, RMP, AP amplitude (APA), and V_max_ values (B) for the indicated lines paced at 1 Hz. ^∗^Statistical significance to KCNH2^WT/WT^ (KCNH2^PR/WT^: APD_50_, p < 0.05; APD_90_, p < 0.001; KCNH2^TL/WT^: APD_50_, p < 0.01; APD_90_, p = 0.0001; one-way ANOVA with Tukey's multiple comparisons test). Values (n) refer to the number of individual hiPSC-CMs analyzed. (C) Consecutive AP traces from a KCNH2^PR/WT^ (left) and KCNH2^TL/WT^ (right) hiPSC-CM with and without (solid and dotted lines, respectively) oscillations in membrane potential interrupting the repolarization and indicating the occurrence of EADs. (D) Percentage of the indicated lines displaying EADs when paced at 0.2 Hz. Values (n) refer to the number of individual hiPSC-CMs analyzed. (E and F) Representative MEA traces (E) and average values for beat period and cFPD (F) for the indicated lines. ^∗^Statistical significance to KCNH2^WT/WT^ (KCNH2^PR/WT^: beat period, cFPD, p < 0.0001; KCNH2^TL/WT^: cFPD, p < 0.0001; one-way ANOVA with Tukey's multiple comparisons test. #Significance between KCNH2^PR/WT^ and KCNH2^TL/WT^ (beat period, cFPD, p < 0.0001). Values (n) refer to the number of independent wells analyzed from at least four differentiations for each cell line. G) Representative averaged time plots of baseline-normalized fluorescence signals for the indicated lines stimulated at 1.2 Hz. AP traces are shown in blue, cytosolic Ca^2+^ flux in orange, and contraction-relaxation in red. (H) Average APD_90_, CaD_90_, and ContD_90_ values for the indicated lines as determined by changes in fluorescent signal. ^∗^Statistical significance to KCNH2^WT/WT^ (KCNH2^PR/WT^: APD_90_, CaD_90_, ContD_90_, p ≤ 0.0001; KCNH2^TL/WT^: APD_90_, ContD_90_, p < 0.05; CaD_90_, p < 0.001); #statistical significance between KCNH2^PR/WT^ and KCNH2^TL/WT^ (APD_90_, ContD_90_, p < 0.05); n = 35–39 recordings from three differentiations for each cell line (Student's t test following one-way ANOVA with Tukey's multiple comparisons test). Error bars represent SEM. See also Figure S3.

To determine whether the differences in I_Kr_ density between the two KCNH2 variants was also reflected in the action potential duration (APD), action potentials (APs) from individual hiPSC-CMs were recorded (Figure 3A). All cells measured had AP plateau amplitude values >85 mV (Figure S3A), indicating that the hiPSC-CMs analyzed were all ventricular-like (Devalla et al., 2015; Veerman et al., 2016). The APD at 50% and 90% repolarization (APD_50_ and APD_90_, respectively) were significantly prolonged for both KCNH2 variant lines (KCNH2^PR/WT^: 185 ± 17 ms [APD_50_], 243 ± 19 ms [APD_90_]; KCNH2^TL/WT^: 198 ± 16 ms [APD_50_], 254 ± 18 ms [APD_90_]) compared with the KCNH2^WT/WT^ hiPSC-CMs (131 ± 11 ms [APD_50_], 151 ± 11 ms [APD_90_]), although there was no significant difference between the two KCNH2 variant lines (Figure 3B). At both slower and faster pacing frequencies (0.2–4 Hz), the differences in APD_90_ between the KCNH2 variant and wild-type lines remained, without any significant differences between the KCNH2^PR/WT^ and KCNH2^TL/WT^ hiPSC-CMs (Figure S3B and S3C). In addition, arrhythmogenic activity, as evidenced by the presence of early after depolarizations (EADs) during 0.2 Hz stimulation, were only detected in the KCNH2 variant lines with no difference in the frequency of EADs between the two lines (KCNH2^PR/WT^: 18.2%; KCNH2^TL/WT^: 17.6%) (Figures 3C and 3D). No significant differences in AP amplitude and resting membrane potential (RMP) were observed between any of the lines, and while upstroke velocity (V_max_) appeared faster in the KCNH2^PR/WT^ hiPSC-CMs, this was not significant (p = 0.07) (Figure 3B).

Next, we investigated whether differences could be detected between the KCNH2^PR/WT^ and KCNH2^TL/WT^ hiPSC-CMs if measured in a syncytium. Figure 3E shows representative field potential (FP) recordings of the hiPSC-CMs obtained from a multi-electrode arrays (MEA) platform. Differences in beating frequency were observed, with the KCNH2^PR/WT^ hiPSC-CMs showing a significantly shorter beat period than the KCNH2^WT/WT^ and KCNH2^TL/WT^ hiPSC-CMs (1,985 ± 43 versus 3,515 ± 126 and 3,342 ± 102 ms, respectively). Therefore, the FPD was corrected (cFPD) for the beat rate according to Fridericia's formula (Figure 3F). Mirroring the APD differences observed in single hiPSC-CMs, the cFPD was prolonged in KCNH2^PR/WT^ and KCNH2^TL/WT^ hiPSC-CMs (438 ± 24 and 320 ± 13 ms, respectively) compared with KCNH2^WT/WT^ hiPSC-CMs (196 ± 9 ms). Importantly, the cFPD of the KCNH2^PR/WT^ hiPSC-CMs was significantly prolonged compared with the KCNH2^TL/WT^ hiPSC-CMs. The second KCNH2^PR/WT^ and KCNH2^TL/WT^ clones corroborated these findings, indicating that the differences observed were not clone specific (Figure S3D).

Finally, we evaluated the KCNH2 variant lines using a high-speed optical system that can simultaneously measure the APs, intracellular Ca^2+^ transients and contraction-relaxation kinetics of hiPSC-CM monolayers under paced conditions (van Meer et al., 2019). This enables the rapid assessment of how LQT2-causing mutations affect the complete excitation-contraction coupling cascade. Representative transients of the three measured parameters for each of the lines are shown in Figure 3G. Analysis of the voltage traces also showed a significant increase in APD_90_ for both the KCNH2^PR/WT^ and KCNH2^TL/WT^ hiPSC-CMs (403 ± 35 and 350 ± 21ms, respectively) compared with the KCNH2^WT/WT^ hiPSC-CMs (297 ± 33.5 ms; Figure 3H). Both Ca^2+^ transient and contraction at 90% duration (CaD_90_ and ContD_90_, respectively) were also significantly prolonged in the KCNH2 variant lines compared with the wild-type hiPSC-CMs (KCNH2^WT/WT^: 570 ± 8 ms [CaD_90_], 429 ± 18 ms [ContD_90_]; KCNH2^PR/WT^: 620 ± 10 ms [CaD_90_], 492 ± 23 ms [ContD_90_]; KCNH2^TL/WT^: 610 ± 9 ms [CaD_90_], 456 ± 6 ms [ContD_90_]).

Also here, the APD_90_ as well as the ContD_90_ of the KCNH2^PR/WT^ hiPSC-CMs were significantly prolonged compared with the KCNH2^TL/WT^ hiPSC-CMs. Although the CaD_90_ appeared prolonged, this did not reach significance (p = 0.05). These results indicate that when the two variants are examined as confluent monolayers, differences in I_Kr_ density are also reflected in the electrophysiological phenotype of the hiPSC-CMs, with the dominant-negative-causing *KCNH2* mutation leading to a more pronounced increase in cFPD and APD than the haploinsufficiency-causing *KCNH2* mutation. Furthermore, these differences are also reflected in both intracellular Ca^2+^ transients and contraction-relaxation kinetics, suggesting that these parameters are also differentially influenced by mild and severe LQT2-causing mutations.

### KCNH2^PR/WT^ and KCNH2^TL/WT^ hiPSC-CMs Exhibit Differing Sensitivities to E-4031

**Figure 4:**
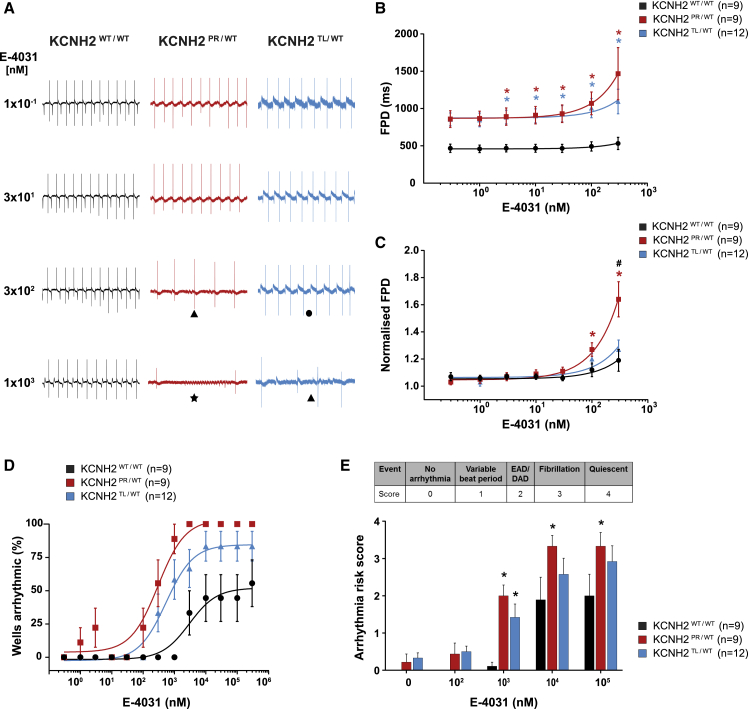
Effect of I_Kr_ Blockade on FPD and Arrhythmogenesis in KCNH2^WT/WT^, KCNH2^PR/WT^, and KCNH2^TL/WT^ hiPSC-CMs (A) Representative MEA traces highlighting the differences between the indicated lines in the development of arrhythmic events during a recording as E-4031 is cumulatively added. Symbols indicate examples of the different types of arrhythmias detected: (•) variable beat period; (▴) abnormal repolarizations; (★) fibrillation. (B and C) FPD (B) and FPD normalized to baseline (C) of the indicated lines upon accumulative addition of E-4031. ^∗^Statistical significance to KCNH2^WT/WT^ (FPD: 3–300 nM, p < 0.05; normalized FPD: 100 nM, p < 0.05; 300 nM, p < 0.0001); #statistical significance between KCNH2^PR/WT^ and KCNH2^TL/WT^ (p < 0.0001); two-way ANOVA with Tukey's multiple comparisons test. (D) Scatterplot illustrating relationship between occurrence of arrhythmic events and concentration of E-4031 for the indicated lines. Curve fitting with nonlinear regression. (E) Arrhythmia risk scoring system and bar graph summarizing the arrhythmia risk for each of the cell lines at different concentrations of E-4031. EAD/DAD, abnormal repolarization; ^∗^statistical significance to KCNH2^WT/WT^ (p < 0.05; two-way ANOVA with Tukey's multiple comparisons test). Values in figure (n) refer to the number of independent wells analyzed from at least three differentiations for each cell line. Error bars represent SEM. See also Figure S4.

To determine if the electrophysiological differences observed between the three lines also led to differing responses to known arrhythmogenic compounds, we examined the response of the hiPSC-CMs to E-4031 (Figure 4). Figure 4A shows representative FP recordings in the presence of increasing concentrations of E-4031, with arrhythmic responses, such as EAD-like events or fibrillations, detected in all three lines. As spontaneous beating ceased in some recordings when the cells were exposed to >300 nM E-4031 (Figure S4A), analysis of the effect of E-4031 on FPD prolongation was performed up to this concentration. The FPD of KCNH2^PR/WT^ and KCNH2^TL/WT^ hiPSC-CMs was significantly prolonged compared with KCNH2^WT/WT^ hiPSC-CMs at >1 nM E-4031 (Figure 4B). When FPD was normalized to baseline measurements, the change in FPD at 300 nM was significantly different for the KCNH2^PR/WT^ hiPSC-CMs compared with the other two lines (Figure 4C), indicating that the KCNH2^PR/WT^ hiPSC-CMs were more sensitive to I_Kr_ blockade. Although the beat period between cell lines varied, it was unaffected for both the KCNH2^TL/WT^ and KCNH2^WT/WT^ hiPSC-CMs at <10 μM E-4031 (Figure S4B). The cFPD was also examined, with differences between the KCNH2^PR/WT^ hiPSC-CMs and the other two lines still discernible (Figures S4C and S4D).

The second KCNH2^PR/WT^ and KCNH2^TL/WT^ clones also showed similar differences in sensitivity to E-4031 following FPD normalization, with analysis performed to 100 nM E-4031 due to some KCNH2^PR/WT^ hiPSC-CMs becoming quiescent at 300 nM (Figures S4A and S4E). Analysis of cFPD showed a similar trend, although it was not significant (Figure S4E), possibly due to overcompensation of the FPD beat rate dependence for the KCNH2^TL/WT^ cl2 hiPSC-CMs (Figures S4F and S4G). Overall, the multiple analyses we have performed with two separate clones for each mutation clearly demonstrate that the KCNH2-A561T mutation results in hiPSC-CMs that are more sensitive to I_Kr_ blockade than the KCNH2-N996I mutation.

To determine if these E-4031-induced differences in FPD between the lines also led to changes in the frequency of arrhythmia-like events, we examined the FP recordings for the occurrence of abnormal repolarizations, fibrillation, and quiescence. Persistent E-4031-induced arrhythmic events were first detected in the KCNH2^PR/WT^, KCNH2^TL/WT^, and KCNH2^WT/WT^ hiPSC-CMs at 100 nM, 300 nM, and 1 μM, respectively (Figure S4A). We also quantified the proportion of recordings that exhibited these arrhythmic responses with increasing concentrations of E-4031 (Figures 4A and 4D). Here too the KCNH2^PR/WT^ hiPSC-CMs were the most predisposed with 100% of recordings showing such events at ≥3 μM E-4031, followed by the KCNH2^TL/WT^ hiPSC-CMs with >80% of recordings becoming arrhythmic; while <55% of KCNH2^WT/WT^ hiPSC-CMs recordings showed such a response even at the highest E-4031 concentration (300 μM). The E-4031 concentration that resulted in 50% of the maximal response was also significantly different between the lines (KCNH2^PR/WT^: 298 nM; KCNH2^TL/WT^: 536 nM; KCNH2^WT/WT^: 2.98 μM; p < 0.01). This increased susceptibility to E-4031-induced arrhythmia-like events in the KCNH2^PR/WT^ hiPSC-CMs was also observed in the second set of KCNH2^PR/WT^ and KCNH2^TL/WT^ clones examined (Figure S4H).

Finally, we investigated the possibility of developing a scoring system based on methods described previously (Blinova et al., 2018; Shaheen et al., 2018) to estimate the arrhythmogenic risk to E-4031 for the different hiPSC-CM lines. We included variable beat period (score 1), which has previously been classified as a “mild” arrhythmia type (Blinova et al., 2018) as an additional category. Abnormal repolarization was given a score of 2, while hiPSC-CMs that were fibrillating or became quiescent were scored 3 and 4, respectively. Both KCNH2^PR/WT^ and KCNH2^TL/WT^ hiPSC-CMs had higher arrhythmia risk scores compared with the KCNH2^WT/WT^ hiPSC-CMs at all concentrations of E-4031 analyzed, with both lines significantly greater at 1 μM E-4031 and these differences remaining between the KCNH2^PR/WT^ and KCNH2^WT/WT^ hiPSC-CMs at higher concentrations (Figure 4E). Differences in the arrhythmia risk score were also observed between the second KCNH2^PR/WT^ and KCNH2^TL/WT^ clones (Figure S4I). Taken together, these results demonstrate a difference in susceptibility to arrhythmias between the variant lines and the KCNH2^WT/WT^ hiPSC-CMs, with KCNH2^PR/WT^ hiPSC-CMs more sensitive to E-4031 than the KCNH2^TL/WT^ hiPSC-CMs.

## Discussion

Interpreting the functional consequences of potential disease-causing variants in LQTS patients is often inconclusive due to the variable expressivity and incomplete penetrance of these diseases (Giudicessi and Ackerman, 2013), as well as the high level of background genetic variation observed in LQTS-susceptibility genes (Giudicessi et al., 2012). The ability to generate hiPSCs from patients, combined with advances in genome editing technologies, has demonstrated how such a platform can be used to determine the pathogenicity of variants of uncertain significance (Garg et al., 2018; Ma et al., 2018), or the contribution of genetic modifiers to the disease phenotype (Chai et al., 2018). However, the extent to which hiPSCs can reflect intragenotype differences in disease risk such as that observed between LQT2 patients has not been fully explored (van den Brink et al., 2020). Here, we provide evidence that genetically matched hiPSC lines can model differences in disease severity attributable to the *KCNH2* mutation.

Most *KCNH2* mutations in the cytoplasmic tail cause haploinsufficiency, with mutant subunits failing to co-assemble with wild-type subunits (Kaufman, 2013). This results in approximately half of the usual number of functional channels and a ≤50% reduction in I_Kr._ In contrast, *KCNH2* mutations within the pore region typically have dominant-negative effects (Anderson et al., 2014), thereby resulting in even fewer functional channels and a >50% I_Kr_ reduction (Kaufman, 2013; Vandenberg et al., 2012). In the hiPSC-CMs we detected a <50% I_Kr_ decrease with the KCNH2-N996I mutation and an ∼70% reduction for the KCNH2-A561T mutation, indicating haploinsufficiency and dominant-negative effects, respectively, but also suggesting that tetrameric ion channels containing one KCNH2-A561T subunit remain functional. A previous study evaluating KCNH2-A561T in COS-7 cells observed an almost identical reduction in I_Kr_ current density (70%) (Bellocq et al., 2004). While heterologous expression systems are simple to use and have the advantage that I_Kr_ kinetics can be studied without the need to block other ion channels, the effect of these changes on the functionality of the cardiomyocyte cannot be directly ascertained and the channel is expressed at unnaturally high levels. In contrast, hiPSC-CMs offer a more native cell environment, with hERG as well as other proteins involved in the transduction of the current, such as auxiliary channel subunits, expressed closer to physiological levels, and functional implications on the AP directly measurable.

We hypothesized that the differences in I_Kr_ would also be reflected in the electrophysiological phenotype of the hiPSC-CMs. All three platforms used to assess the electrophysiology of the lines demonstrated a clear prolongation of the APD and FPD for both KCNH2 variant lines compared with the KCNH2^WT/WT^ hiPSC-CMs, with recordings made using confluent monolayers of hiPSC-CMs revealing that the KCNH2^PR/WT^ lines had longer FPs and APs compared with the KCNH2^TL/WT^ hiPSC-CMs. The discrepancy between the data measured by patch clamp compared with the MEA or optical recordings is possibly due to differences in the setup and cellular configuration between the experimental approaches. Sparsely seeded hiPSC-CMs, such as those used for patch-clamp recordings, show greater electrophysiological variability compared with measurements performed on confluent monolayers (Du et al., 2015), which could confound the detection of subtle electrophysiological differences. In addition, both the MEA and optical system offer analysis at higher throughput than traditional patch-clamp techniques, enabling larger numbers of cells to be measured and obtain statistical confidence where phenotypic differences are expected to be small.

We also evaluated the cytosolic Ca^2+^ transients and contraction kinetics of all three lines, as these are also key parameters that can be altered in LQTS (Kiviaho et al., 2015; Leren et al., 2015; Sala et al., 2018; Spencer et al., 2014). As expected, both LQT2-causing mutations resulted in a significant prolongation in Ca^2+^ transients and contraction-relaxation duration when compared with KCNH2^WT/WT^ hiPSC-CMs. We also observed significant differences in contractility kinetics between the two KCNH2 variants. Contraction duration differences are known to exist between symptomatic and asymptomatic LQTS patients (Haugaa et al., 2010), and this has been proposed as an additional parameter to measure alongside QT interval for improving risk stratification in LQTS patients (ter Bekke et al., 2015).

In LQT2 patients, sudden arousal is the most frequent trigger of an arrhythmic cardiac event (Wilde et al., 1999), and patients with mutations in the pore-loop region have a significantly increased risk to this and other (e.g., fever, medication, sleep) triggering factors (Kim et al., 2010). To mimic the effect of such triggers, we examined the behavior of the hiPSC-CMs when treated with the I_Kr_ blocker, E-4031. We observed differing responses to the QT-prolonging drug between the three lines, with the KCNH2^PR/WT^ hiPSC-CMs exhibiting a greater prolongation in normalized FPD than the KCNH2^TL/WT^ or KCNH2^WT/WT^ hiPSC-CMs. These differences in sensitivity to I_Kr_ block could be due to the dominant-negative effect of the KCNH2-PR mutation, additional gating kinetic defects in trafficked hERG channels that include the KCNH2-A561T subunit (Perry et al., 2016), or a combination of both. The results differ from those recently reported by Yoshinaga et al. (2019), who observed a smaller change in FPD in response to I_Kr_ blockade in hiPSC-CMs derived from LQT2 patients than in control or mutation-corrected hiPSC-CMs. This discordance could be mutation-specific as they also observed an increased arrhythmia susceptibility with the LQT2 hiPSC-CMs.

In line with their differing FPD response to I_Kr_ blockade, the KCNH2^PR/WT^ hiPSC-CMs also exhibited an increased occurrence of arrhythmia events in the presence of E-4031. In addition, a greater proportion of measurements from KCNH2^TL/WT^ hiPSC-CMs displayed arrhythmic activity compared with KCNH2^WT/WT^ hiPSC-CMs. Akin to systems being established to evaluate the arrhythmogenic risk of pharmacological compounds (Blinova et al., 2018), a similar matrix could be developed to assess the risk of specific mutations in patients to different triggering conditions. As proof-of-concept, we determined the arrhythmia risk score for all three lines at various concentrations of E-4031, observing that the KCNH2^PR/WT^ hiPSC-CMs had a higher arrhythmogenic risk at 1 μM. It will be necessary to further evaluate this scoring system with a larger panel of *KCNH2* mutations as well as for different triggers, but this study suggests that genetically matched sets of hiPSC-CMs are sufficiently sensitive to detect these subtle intragenotype-phenotype mutational differences. Such an approach could have clinical implications, for example, by identifying particular *KCNH2* mutations that predispose patients to increased arrhythmic risk and whom might benefit from more vigilant monitoring.

Moreover, other methods such as *in silico* prediction tools still perform poorly in even correctly classifying benign and pathogenic mutations (Musunuru et al., 2018). This study highlights the benefit of introducing mutations into a well-characterized control hiPSC line. Indeed, while technically challenging, this approach is one of the only options to identify subtle mutation-specific functional changes, as genetic background differences would likely mask these if studied in patient-derived hiPSCs. This strategy also means that the mutations examined are not limited by the availability of patient material. In addition, this method is cheaper and faster as the isogenic control for each variant does not need to be generated and characterized. While common variants present in the control hiPSC line might modify the disease phenotype, in our approach all mutations are evaluated on the same genetic background, thereby nullifying their effect. Furthermore, as similar responses were detected in both clonal lines generated for each mutation, the differences observed between the experimental groups are unlikely to be due to variants that have arisen spontaneously in culture or from CRISPR-Cas9-induced off-target effects.

In conclusion, we have established that genetically matched hiPSC-CMs can capture electrophysiological differences related to the *KCNH2* mutation, with these differences also reflected in the occurrence of drug-induced arrhythmias. This study demonstrates an application in which hiPSC-CMs could be used to model mutation-location risk differences seen in LQT2 patients and contribute to improvements in the diagnosis, prognosis, and risk stratification of patients with congenital LQTS.

## Experimental Procedures

An extended methods section is provided in the Supplemental Information.

### Genome Editing

The *KCNH2* variants (c.G1681A and c.A2987T) were introduced into a hiPSC control (KCNH2^WT/WT^; LUMC0020iCTRL-06) (Zhang et al., 2014) line by CRISPR-Cas9-mediated gene editing. Heterozygosity of clones was confirmed by Sanger sequencing. Sequences of the guide RNAs, single-strand oligonucleotides (ssODNs), and PCR primers used in this study are listed in Tables S3 and S4.

### Differentiation to hiPSC-CM

The hiPSC lines were differentiated into cardiomyocytes as described in the Supplemental Information. All analyses were performed on cryopreserved hiPSC-CMs 5–9 days after thawing.

### Electrophysiology

Voltage-clamp recordings of I_Kr_ were made using pipette and bath solutions as described previously (Bellin et al., 2013). APs of the hiPSC-CMs were recorded by perforated patch clamp and the dynamic clamp technique with injection of an inward rectifier potassium current (I_K1_) used to achieve a close-to-physiological RMP (Verkerk et al., 2017). For electrophysiological analysis on MEAs, the FP was recorded as described previously (Sala et al., 2017). Sequential addition of increasing concentrations of E-4031, a specific I_Kr_ blocker, was performed with recordings initiated following 1 min of incubation.

### Optical Recordings

The hiPSC-CMs were labeled with organic fluorescent dyes and the resulting signals recorded and analyzed as described in the Supplemental Information.

### Statistical Analysis

Results are presented as mean ± SEM, with comparison between groups performed using one-way or two-way ANOVA followed by Tukey's multiple comparisons test for *post hoc* analysis. Pairwise comparisons were also performed using the Student's t test following one-way ANOVA if one of the null hypotheses could be rejected (Goeman and Solari, 2011). Curve fitting from regression models and statistical analyses was performed using GraphPad Prism 8 software, with p < 0.05 considered statistically significant.

### Data and Code Availability

Requests for the data used in this paper, including the genome sequencing data, should be directed to and will be fulfilled by the corresponding author.

## Author Contributions

Conceptualization, K.O.B. and R.P.D.; Methodology, K.O.B., L.v.d.B., D.C.M., C.G., B.J.v.M., M.P.H.M., T.d.K., and R.P.D.; Investigation, K.O.B., L.v.d.B., D.C.M., T.d.K., A.O.V., and R.P.D.; Supervision, C.L.M. and R.P.D.; Formal Analysis, K.O.B., L.v.d.B., D.C.M., T.d.K., L.S., and R.P.D.; Software, B.J.v.M. and L.G.J.T.; Writing – Original Draft, K.O.B. and R.P.D.; Writing – Review & Editing, K.O.B., L.v.d.B., D.C.M., C.G., B.J.v.M., C.L.M., L.S., and R.P.D.; Funding Acquisition, R.P.D.

## Conflicts of Interests

C.L.M. is a cofounder of Pluriomics B.V. (now Ncardia B.V.).

## Supplemental Information

Document S1. Supplemental Experimental Procedures, Figures S1–S4, and Tables S2–S4

Table S1. Coding Sequence SNPs and INDELs in Genes Associated with Cardiac Arrhythmias and Cardiomyopathies in hiPSC Line LUMC0020iCTRL04 (KCNH2WT/WT), Related to Figure 1

Document S2. Article plus Supplemental Information
