# Supplementary material for: Isogenic Sets of hiPSC-CMs Harboring Distinct KCNH2 Mutations Differ Functionally and in Susceptibility to Drug-Induced Arrhythmias
Source: Stem Cell Reports. 2020 Nov 10;15(5):1127–39. doi: 10.1016/j.stemcr.2020.10.005 (PMC7664051; doi:10.1016/j.stemcr.2020.10.005)
Supplement: Document S1. Supplemental Experimental Procedures, Figures S1–S4, and Tables S2–S4 [file mmc1.pdf]

Stem Cell Reports, Volume *15*

Supplemental Information

Isogenic Sets of hiPSC-CMs Harboring Distinct *KCNH2* Mutations Differ Functionally and in Susceptibility to Drug-Induced Arrhythmias

Karina O. Brandão, Lettine van den Brink, Duncan C. Miller, Catarina Grandela, Berend J. van Meer, Mervyn P.H. Mol, Tessa de Korte, Leon G.J. Tertoolen, Christine L. Mummery, Luca Sala, Arie O. Verkerk, and Richard P. Davis

Supplemental Figures

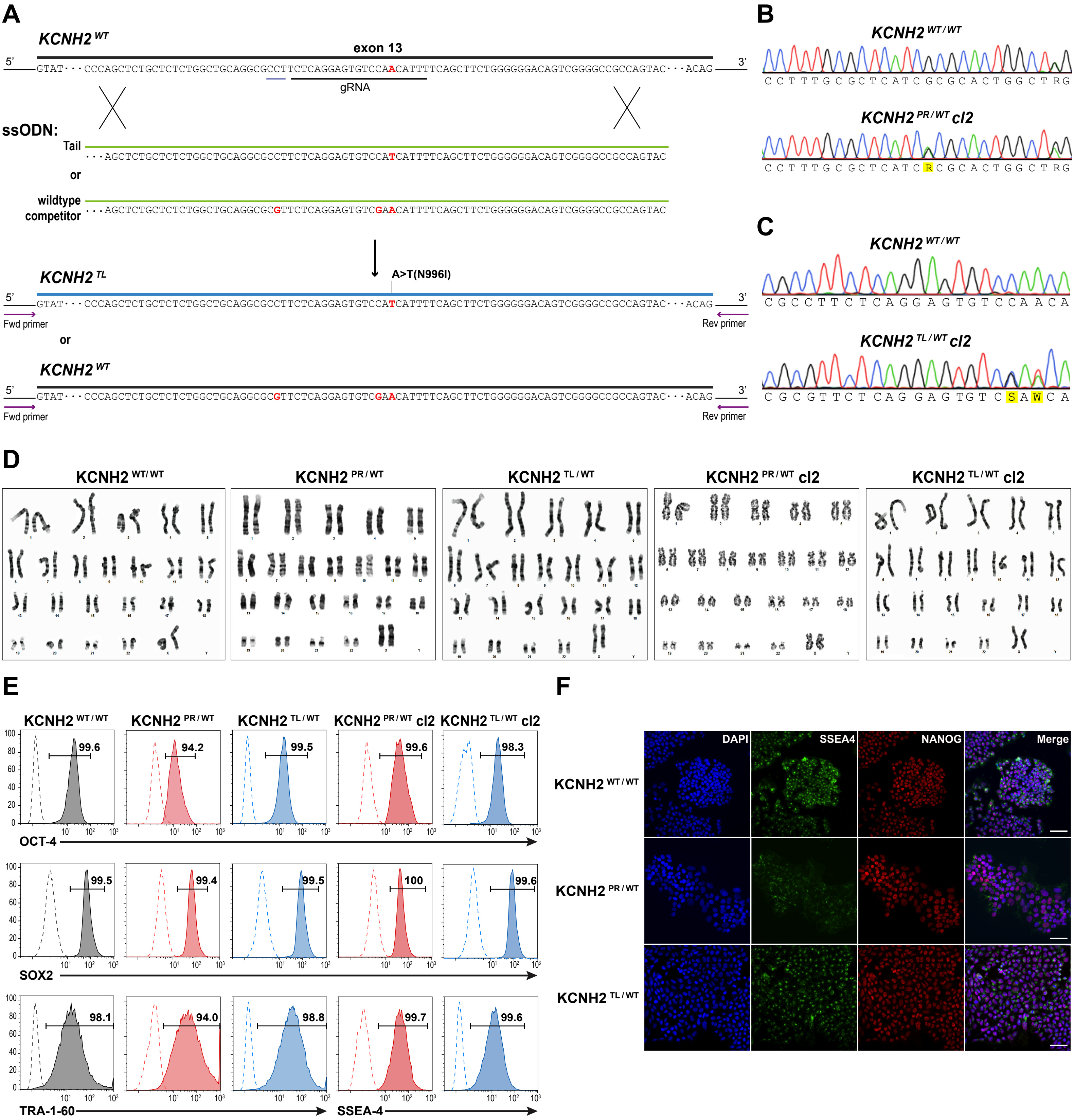

Figure S1: Generation and characterisation of the set of isogenic KCNH2 hiPSC lines. Related to Figure 1. (A) Schematic outlining the alternative gene editing strategy used to generate the KCNH2 $^{TL/WT}$ cl2 hiPSC line. The *ssODN Tail* included the c.A2987T (N996I) mutation (red), while the *ssODN wildtype competitor* included silent nucleotide mutations (red) that did not alter any amino acids but assisted with RFLP screening and prevented Cas9 from cutting the modified sequence due to a mutation in the PAM sequence (grey underline).Sequence analysis of PCR-amplified DNA showing **(B)** heterozygous introduction of the c.G1681A (A561T) mutation (highlighted R) in the KCNH2 $^{PR/WT}$ cl2 hiPSC line, and **(C)** heterozygous introduction of the c.A2987T (N996I) mutation (highlighted W), as well as the RFLP screening silent mutation (highlighted S), in the KCNH2 $^{TL/WT}$ cl2 hiPSC line. **(D)** G-band karyograms indicating a normal euploid karyotype for the KCNH2 hiPSC lines used in the study. **(E)** Flow cytometry and **(F)** immunofluorescence analysis of the pluripotency-associated markers, NANOG, OCT4, SOX2, TRA-1-60 and SSEA4 in the indicated hiPSC lines. Scale bar: 50 µm.

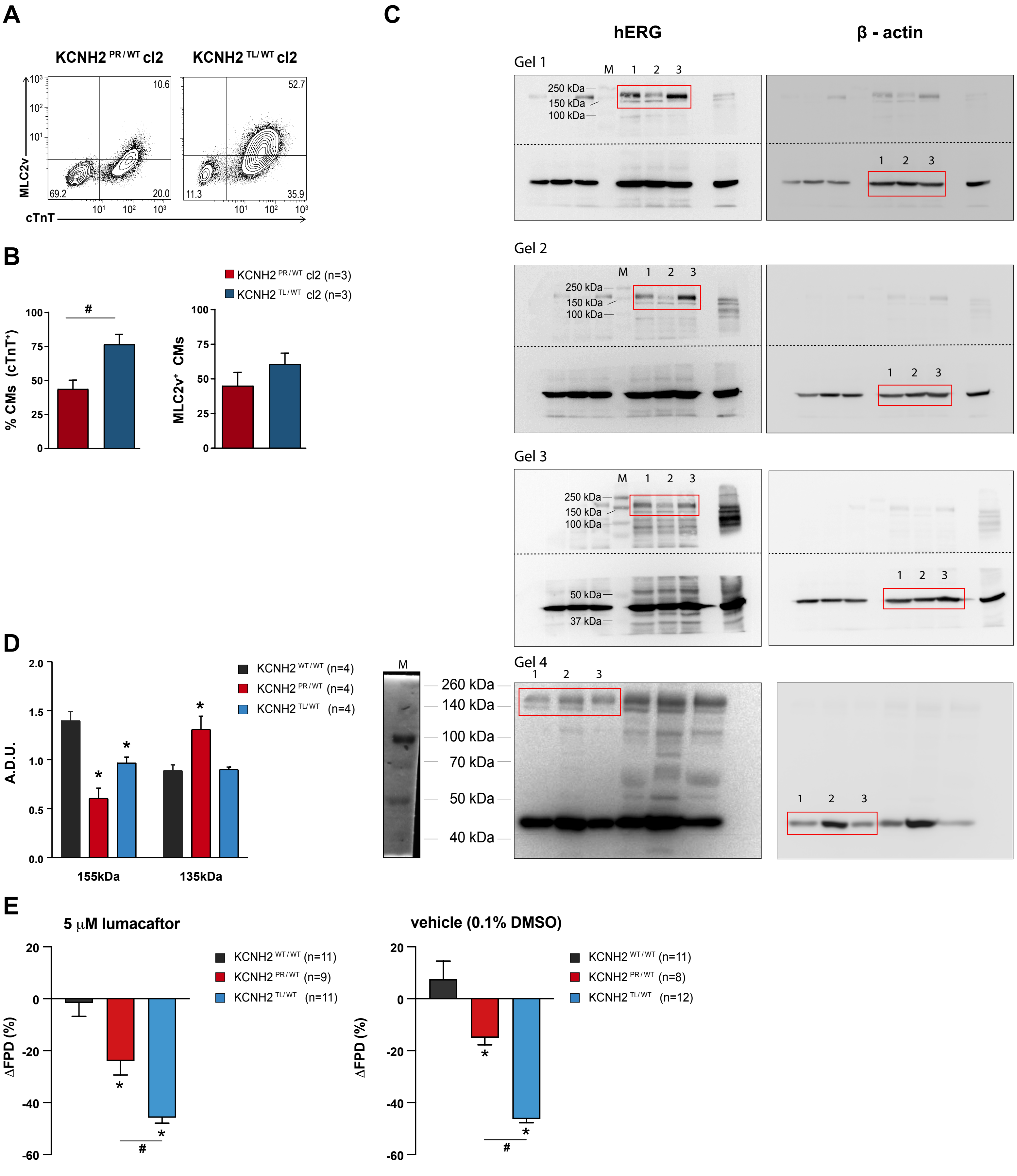

Figure S2: Evaluation of effect of KCNH2 mutations on hERG channel function in hiPSC-CMs. Related to Figure 2. Representative flow cytometry plots **(A)** and overall cardiac differentiation efficiency to ventricular-like cardiomyocytes **(B)** for the KCNH2$^{PR/WT}$ cl2 and KCNH2$^{TL/WT}$ cl2 hiPSC lines (#, *P*=0.02 (two-tailed Student's t-test); *n*, number of independent differentiations). **(C)** Full western blots showing detection of hERG protein bands and β-actin (loading control) in the hiPSC-CMs. Order is: protein marker (M); KCNH2$^{TL/WT}$ (1); KCNH2$^{PR/WT}$ (2); and KCNH2$^{WT/WT}$ (3) hiPSC-CMs. Dotted lines indicate where membrane was cut. The hERG and β-actin bands used for quantification are marked by red boxes. **(D)** Densitometric quantification of the 155 kDa and 135 kDa bands (normalised to β-actin); A.D.U., arbitrary densitometric units (*, *P*<0.01 compared to KCNH2$^{WT/WT}$ (two-way ANOVA with Tukey's multiple comparisons for post hoc test); *n*, number of independent differentiations). **(E)** Percent change in FPD relative to baseline (ΔFPD) after 8 days of treatment with either 5 μM lumacaftor (*left*) or vehicle control (0.1% DMSO; *right*) for the indicated hiPSC-CM lines. A negative value indicates shortening of the FPD. * indicates significance to KCNH2$^{WT/WT}$ (KCNH2$^{PR/WT}$, *P*<0.01; KCNH2$^{PR/WT}$, *P*<0.0001); # indicates statistical significance between KCNH2$^{PR/WT}$ and KCNH2$^{TL/WT}$ (lumacaftor, *P*<0.01; DMSO, *P*<0.001); *n*, number of wells from at least 3 independent differentiations.

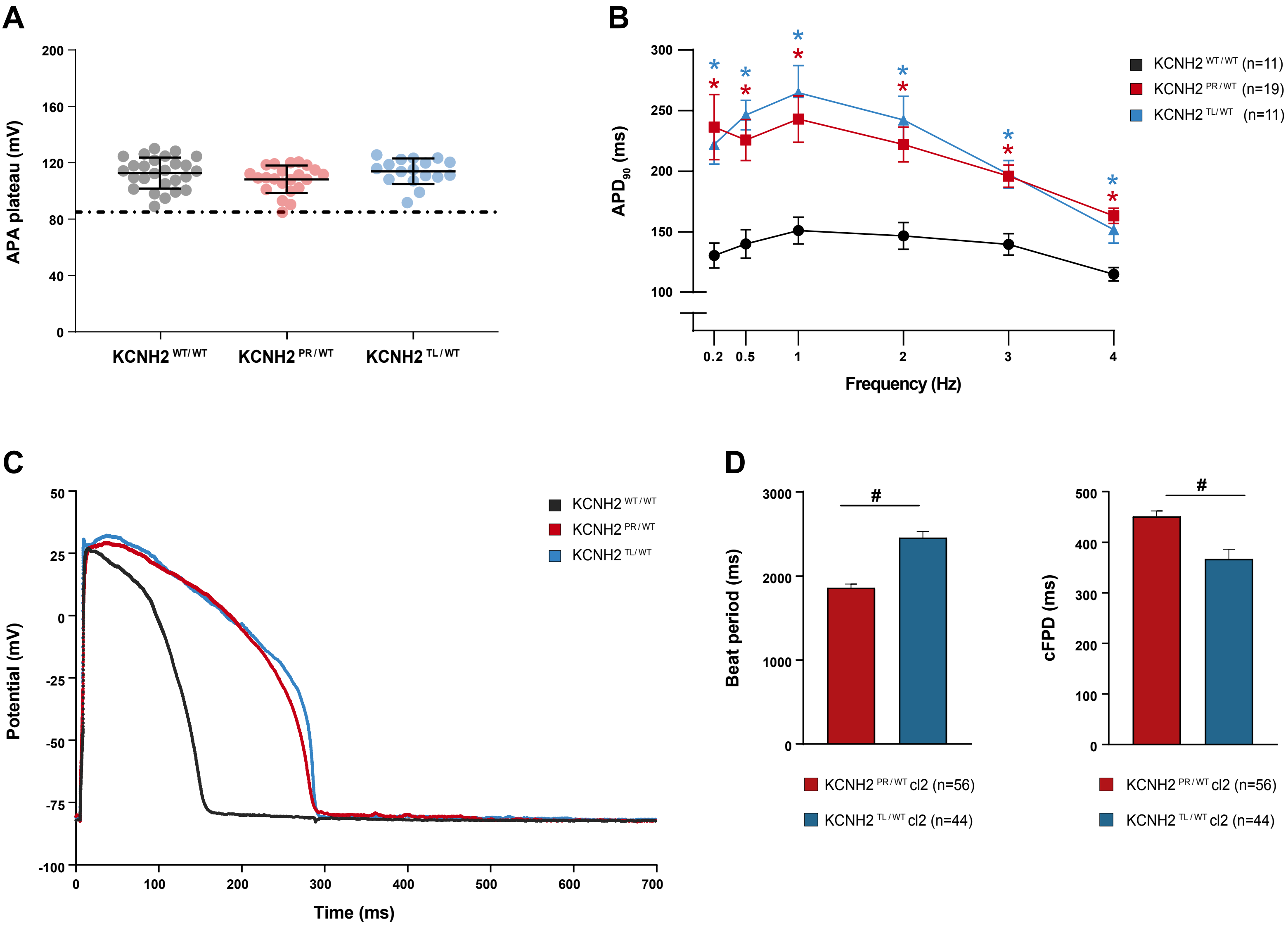

Figure S3: Electrophyisological characterisation of KCNH2 $^{WT/WT}$, KCNH2 $^{PR/WT}$ and KCNH2 $^{TL/WT}$ hiPSC-CMs. Related to Figure 3. (A) Dot plots of AP plateau amplitude measured at 20 ms after initiation of the AP upstroke in the indicated hiPSC-CMs paced at 1 Hz. The dotted line indicates 85 mV. **(B)** Average APD_{90} values for the indicated hiPSC-CMs paced between 0.2–4 Hz. *, P<0.01 compared to KCNH2 $^{WT/WT}$ (two-way ANOVA with Tukey's multiple comparisons); *n*, number of individual hiPSC-CMs analysed. **(C)** Representative examples of non-arrhythmogenic AP traces for indicated hiPSC-CMs paced at 0.2 Hz. **(D)** Average values for beat period interval (*left*) and the cFPD (*right*) for the indicated cell lines. #, P<0.05 (two-tailed Student's t-test); *n,* number of independent wells analysed from at least 3 differentiations.

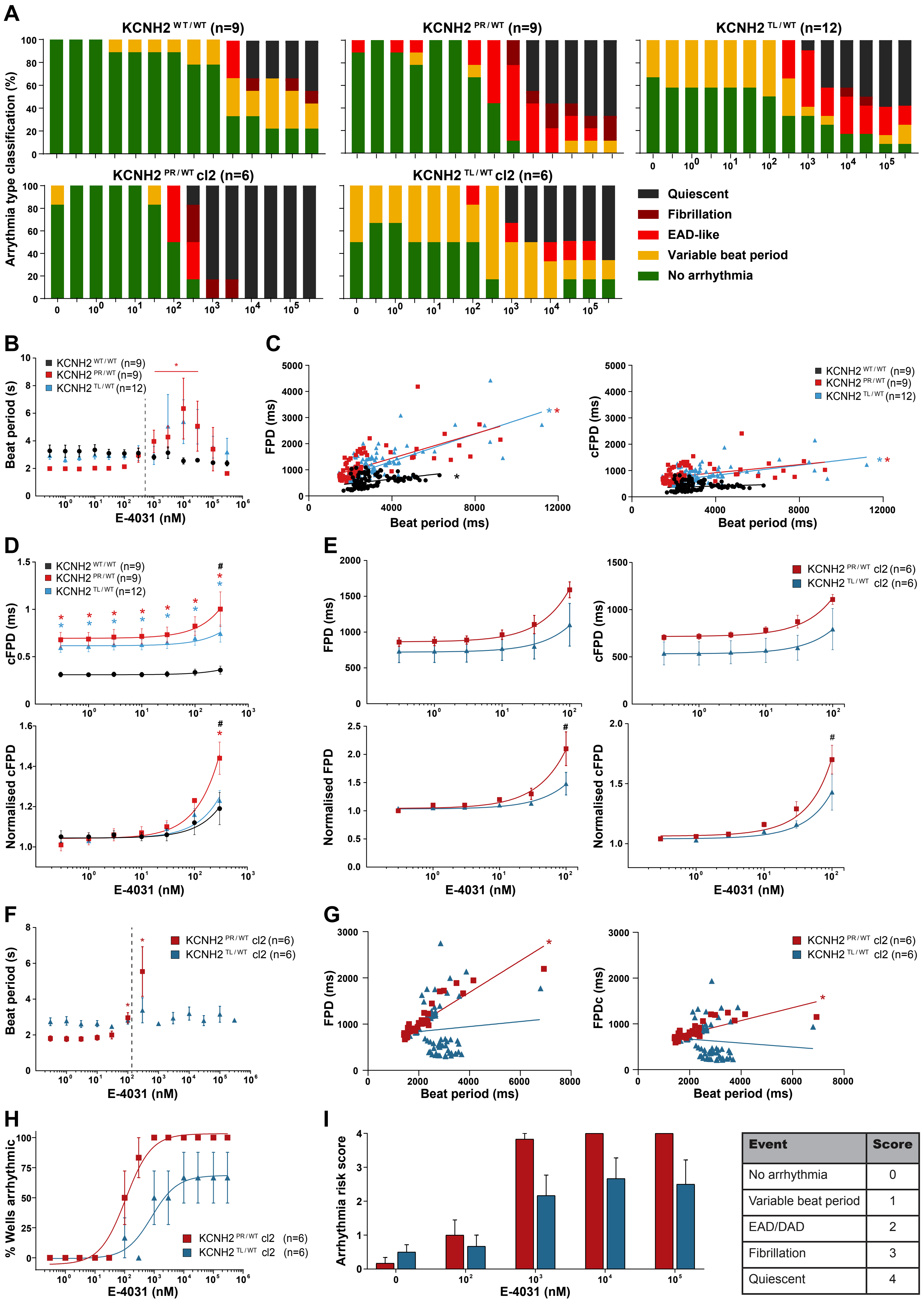

A
KCNH2 WT/WT (n=9)
KCNH2 PR/WT (n=9)
KCNH2 TL/WT (n=12)
KCNH2 PR/WT cl2 (n=6)
KCNH2 TL/WT cl2 (n=6)
Arrythmia type classification (%)
Quiescent
Fibrillation
EAD-like
Variable beat period
No arrhythmia
B
Beat period (s)
E-4031 (nM)
C
FPD (ms)
cFPD (ms)
Beat period (ms)
D
cFPD (ms)
Normalised cFPD
E-4031 (nM)
E
FPD (ms)
cFPD (ms)
Normalised FPD
Normalised cFPD
E-4031 (nM)
F
Beat period (s)
E-4031 (nM)
G
FPD (ms)
FPDc (ms)
Beat period (ms)
H
% Wells arrhythmic
E-4031 (nM)
I
Arrhythmia risk score
E-4031 (nM)
Event
Score
No arrhythmia
0
Variable beat period
1
EAD/DAD
2
Fibrillation
3
Quiescent
4

Figure S4. Analysis of the effect of E-4031 on hiPSC-CMs with different *KCNH2* mutations. Related to Figure 4. (A) Quantification of the arrhythmia subtypes detected with increasing concentrations of E-4031 for the indicated lines. **(B, F)** Analysis of the effect of increasing concentrations of E-4031 (0.3 nM - 300 μM) on the average beat period for the indicated lines. The dotted line demarcates the maximum concentration of E-4031 (300 nM: $KCNH2^{WT/WT}$, $KCNH2^{PR/WT}$ and $KCNH2^{TL/WT}$ hiPSC-CMs; 100 nM: $KCNH2^{PR/WT}$ cl2 and $KCNH2^{TL/WT}$ cl2 hiPSC-CMs) included in the analysis of the effect of this compound on (c)FPD. **(C, G)** Major-axis regression analysis on the relationship between beat period and either FPD (*left graphs*) or cFPD (*right graphs*) for the indicated lines upon increasing concentrations of E-4031. * indicates linear coefficients significantly different from 0 ($P<0.0001$). **(D)** cFPD (*upper graph*) and cFPD normalised to baseline (*lower graph*) of the indicated lines upon accumulative addition of E-4031. * indicates statistical significance to $KCNH2^{WT/WT}$ (cFPD: 0.3-300 nM, $P<0.02$; normalised cFPD: 300 nM, $P<0.0001$); # indicates statistical significance between $KCNH2^{PR/WT}$ and $KCNH2^{TL/WT}$ hiPSC-CMs ($P<0.0001$). **(E)** FPD **(***upper left graph***)**, FPD normalised to baseline **(***lower left graph***)**, cFPD (*upper right graph***)** and cFPD normalised to baseline **(***lower right graph***)** of $KCNH2^{PR/WT}$ cl2 and $KCNH2^{TL/WT}$ cl2 hiPSC-CMs upon accumulative addition of E-4031. # indicates statistical significance between mutated lines ($P<0.006$). **(H)** Scatter plot illustrating relationship between occurrence of arrhythmic events and concentration of E-4031 for $KCNH2^{PR/WT}$ cl2 and $KCNH2^{TL/WT}$ cl2 hiPSC-CMs. Curve fitting with nonlinear regression. **(I)** Arrhythmia risk scoring system and bar graph summarising the arrhythmia risk these two cell lines at different concentrations of E- 4031. Values (*n*) refer to the number of independent wells analysed from at least 3 differentiations for each of the cell lines $KCNH2^{WT/WT}$, $KCNH2^{PR/WT}$ and $KCNH2^{TL/WT}$, and from 2 differentiations for the cell lines $KCNH2^{PR/WT}$ cl2 and $KCNH2^{TL/WT}$ cl2.

Supplemental Tables

Table S2: Predicted gRNA off-target exonic sequences analysed for each *KCNH2* variant hiPSC line. Related to Figure 1.

	Off-target Sequence	Mismatch Position	Mismatch	Score	Chromosome	Strand	Gene
**KCNH2$^{PR/WT}$ clones**	CCGCTACTCTGAGTATGGGGCGG	*........*.....*..*.	4	0.02	chr17	+	***KCNH6***
**KCNH2$^{TL/WT}$ clone 1**	CTACCAGAAAATGAAAATGTGGG	.**......**.........	4	0.55	chrX	-	***BEND2***
	TCCCCAGAAGCAAAAAATTTAGG	*..........**.....*.	4	0.49	chr4	-	***HSPA4L***
	AGCCCAGAAACTGAAAATATTGG	**.......*........*.	4	0.46	chr7	-	***ETV1***
	TCCCCAACAGCTGAAAATGTGGG	*.....**............	3	0.42	chr7	-	***SGCE***
	CTGTAAGAAGCTGAAAATGTTGG	.****...............	4	0.2	chr1	+	***PRAMEF20***
**KCNH2$^{TL/WT}$ clone 2**	AATGCAGGACACTTCAGAGAAGG	....**.......*.*....	4	0.58	chr16	-	**ACSM5**
	AAAGTTGGACATTCCAAAGATGG	..*........*...**...	4	0.33	chr2	+	***SCN9A***
	AATGATGGATAATCCTGAAATGG	....*....*.*......*.	4	0.24	chr14	+	***SYT16***
	AAAGTTGGCGATTCCTGAGACGG	..*.....**.*........	4	0.09	chr7	+	***DLX5***
	TAGGATGGACTCTCCTGAGAAGG	*.*.*.....*.........	4	0.07	chr11	+	***CBL***

Table S3: Sequences of gRNAs and ssODNs used to generate the *KCNH2* variant hiPSC lines. Related to Figure 1

$KCNH2^{PR/WT}$ clones

**gRNA#1**	5'-TCGCTACTCAGAGTACGGCG-3'
**ssODN_pore**	5'-GGCTGCTGCGGCTGGTGCGCGTGGCGCGGAAGCTGGATCGCTACTCAGAGTACGGCGCGGCCGTGCTGTTCTTGCTCA TGTGCACCTTTGCGCTCATCGCGCACTGGCTAGCCTGCATCTGGTACG-3'

$KCNH2^{TL/WT}$ clone 1

**gRNA#2**	5'-TGTAAAAGTCGAAGACCCCC-3'
**ssODN_tail**	5'-GACCGGGCGTGGCAGCGGTGGTGCGTCTACCCCGCTCACCCAGCTCTGCTCTCTGGCTGCAGGCGCCTTCTCAGGAGT GTCCATCATTTTCAGCTTCTGGGGGGACAGTCGGGGCCGCCAGTAC-3'

$KCNH2^{TL/WT}$ clone 2

**gRNA#3**	5'-AATGTTGGACACTCCTGAGA-3'
**ssODN_tail**	5'-GACCGGGCGTGGCAGCGGTGGTGCGTCTACCCCGCTCACCCAGCTCTGCTCTCTGGCTGCAGGCGCCTTCTCAGGAGT GTCCATCATTTTCAGCTTCTGGGGGGACAGTCGGGGCCGCCAGTAC-3'
**ssODN_wt competitor**	5'-GACCGGGCGTGGCAGCGGTGGTGCGTCGACCCCGCTCACCCAGCTCTGCTCTCTGGCTGCAGGCGCCTTCTCAGGAGT GTCCAACATTTTCAGCTTCTGGGGGGACAGTCGGGGCCGCCAGTAC-3'

Table S4: List of primers used in this study. Related to Figures 1 and 2.

Primer name	Sequence (5' - 3')	Purpose
**KCNH2_exon 7_Fwd**	CAAGGAGGCAGGTGGTGTAG	amplification of *KCNH2* exon 7
**KCNH2_exon 7_Rev**	CCTCCAACTTGGGTTCCTCC	amplification of *KCNH2* exon 7
**KCNH2_exon 7_seq_Fwd**	CCCCATCAACGGAATGTG	sequencing of *KCNH2* exon 7
**KCNH2_exon 7_seq_Rev**	CACAGCCAATGAGCATGACG	sequencing of *KCNH2* exon 7
**KCNH2_exon 7_cDNA_Fwd**	CTGATCGGGCTGCTGAAGACT	determining *KCNH2* allele transcript expression for KCNH2$^{PR/WT}$
**KCNH2_exon 7_cDNA_Rev**	CCGAAGATGCTAGCGTACATG	determining *KCNH2* allele transcript expression for KCNH2$^{PR/WT}$
**KCNH6_off_target_Fwd**	GCTCTCACTGCTCCTCCATC	amplification of off-target for gRNA#1
**KCNH6_off_target_Rev**	TTCTCGAGTTGGTGTTGGG	amplification of off-target for gRNA#1
**KCNH6_off_target_seq_Fwd**	CTCATCCATGAAGCCTCCCC	sequencing of off-target for gRNA#1
**KCNH6_off_target_seq_Rev**	GCTGAAGGTGAAGTAGAGGG	sequencing of off-target for gRNA#1
**KCNH2_exon 13_Fwd**	CCCTGAGAGCAGTGAGGATG	amplification of *KCNH2* exon 13
**KCNH2_exon 13_Rev**	GGTGGTCACAGCACTGTAGG	amplification of *KCNH2* exon 13
**KCNH2_exon 13_seq_Fwd**	TCAGGTATCCCGGGCGAC	sequencing of *KCNH2* exon 13
**KCNH2_exon 13_seq_Rev**	CTCCCTCTACCAGACAACACC	sequencing of *KCNH2* exon 13
**KCNH2_exon 13_cDNA_Fwd**	CCCTGAGAGCAGTGAGGATG	determining *KCNH2* allele transcript expression for KCNH2$^{TL/WT}$
**KCNH2_exon 13_cDNA_Rev**	GGTGGTCACAGCACTGTAGG	determining *KCNH2* allele transcript expression for KCNH2$^{TL/WT}$
**KCNH2_gRNA#2_IVT_F**	TGTAATACGACTCACTATAGAATGTTGGACACTCCTGAGAGTTTTAGAGCTAGAAATAGC	Forward primer for in vitro transcription of gRNA#2
**KCNH2_gRNA#3_IVT_F**	TGTAATACGACTCACTATAGCCCCCAGAAGCTGAAAATGTGTTTTAGAGCTAGAAATAGC	Forward primer for in vitro transcription of gRNA#3
**gRNA_IVT_Rev**	AGCACCGACTCGGTGCCACT	Reverse primer for in vitro transcription of gRNA#2 and #3
**Off_T.ex13_#2_BEND2_Fwd**	GGGGAAAATTGGGGAAAGGG	assessing potential off-target for gRNA#2
**Off_T.ex13_#2_BEND2_Rev**	CAGCATGTCATGAACTGCAGG	assessing potential off-target for gRNA#2
**Off_T.ex13_#2_HSPA4L_Fwd**	GGTCATGCCCTAAGTCACAGG	assessing potential off-target for gRNA#2
**Off_T.ex13_#2_HSPA4L_Rev**	GACGGGGTTTTGCCATGTTG	assessing potential off-target for gRNA#2
**Off_T.ex13_#2_ETV1_Fwd**	TCCATTTGCGATTTGGTATGGAG	assessing potential off-target for gRNA#2
**Off_T.ex13_#2_ETV1_Rev**	CTGTCTGGCATGTGGGAGTC	assessing potential off-target for gRNA#2
**Off_T.ex13_#2_SGCE_Fwd**	GGTTACCCAGACCGACCTG	assessing potential off-target for gRNA#2
**Off_T.ex13_#2_SGCE_Rev**	GATGTGTTGTTTCCTCCGCC	assessing potential off-target for gRNA#2
**Off_T.ex13_#2_P.20_Fwd**	GTTTGGTCTGAAGCCATGGC	assessing potential off-target for gRNA#2
**Off_T.ex13_#2_P.20_Rev**	CAGGTGGCCTTCGAGGAAAG	assessing potential off-target for gRNA#2
**Off_T.ex13_#3_VWA8_Fwd**	CTTGACTCCCAGCTCTAGCC	assessing potential off-target for gRNA#3
**Off_T.ex13_#3_VWA8_Rev**	CTTTGGGGACTAAGGTGGGG	assessing potential off-target for gRNA#3
**Off_T.ex13_#3_CBL_Fwd**	CCCCTGCTGTGAGACTTCAG	assessing potential off-target for gRNA#3
**Off_T.ex13_#3_CBL_Rev**	AAGGCAGGGGAAAACTGAGG	assessing potential off-target for gRNA#3
**Off_T.ex13_#3_DLX5_Fwd**	GCAAAAACACACACAAGCGC	assessing potential off-target for gRNA#3
**Off_T.ex13_#3_DLX5_Rev**	GCTGTGACCCCCAATCTACC	assessing potential off-target for gRNA#3
**Off_T.ex13_#3_SYT16_Fwd**	CAGTGAGCCTGAAACACAGC	assessing potential off-target for gRNA#3
**Off_T.ex13_#3_SYT16_Rev**	CATGCCCGGCTCTATGCTAG	assessing potential off-target for gRNA#3
**Off_T.ex13_#3_SCN9A_Fwd**	TGTGTCCCCTACCCTGTTCC	assessing potential off-target for gRNA#3
**Off_T.ex13_#3_SCN9A_Rev**	GGTTCAGTACTTTCTTCAGTGGC	assessing potential off-target for gRNA#3
**Off_T.ex13_#3_ACSM5_Fwd**	GTCCATCTGGGGCATCTGAG	assessing potential off-target for gRNA#3
**Off_T.ex13_#3_ACSM5_Rev**	CTCTGCCTCCCGAGTTCAAG	assessing potential off-target for gRNA#3

Supplemental Methods

Culture of human induced pluripotent stem cells (hiPSCs)

For maintenance of the hiPSC lines, Essential 8™ Medium (Gibco) and Vitronectin (VTN-N; Gibco)-coated plates were used. For passaging, the cells were dissociated with TrypLE Select Enzyme (Gibco) and re-plated in Essential 8™ Medium containing RevitaCell™ Supplement (1:200 dilution; Gibco). For gene editing experiments, the hiPSCs were maintained either on irradiated mouse embryonic fibroblasts in human embryonic stem cell medium as previously described (Davis et al., 2009) or in StemFlex™ Medium (Gibco) according to the manufacturer's instructions.

Differentiation and culture of hiPSC-derived cardiomyocytes (hiPSC-CMs)

One day prior to differentiation, hiPSCs were seeded at 3.75 x 10^4/cm^2 into Matrigel® Matrix (BD)-coated wells in Essential 8™ Medium containing RevitaCell™ Supplement. The hiPSCs were differentiated into cardiomyocytes using the Pluricyte Cardiomyocyte Differentiation Kit (NCardia) according to the manufacturer's instructions, with cells maintained in Medium C until day 19-21 of differentiation. The hiPSC-CMs were dissociated with 5x TrypLE Select Enzyme for 10 min at 37°C and cryopreserved. For replating the hiPSC-CMs, the frozen cells were thawed at 37°C and transferred to a conical tube. Immediately thereafter, 1 ml of BPEL medium (van den Berg et al., 2014) was added dropwise (1 drop every 5 s), followed by ~4.7 ml BPEL (1 drop every 2 s). Cells were pelleted at 250*g* for 3 min, resuspended in Medium C and plated as required.

Exome sequencing

Genomic DNA (gDNA) from KCNH2$^{WT/WT}$ hiPSCs was extracted using the Blood and Cultured Cells DNA Mini Kit (Qiagen), according to manufacturer's instructions. Whole exome sequencing was performed by BGI (Hong Kong) using a HiSeq X platform (Illumina), with the library constructed from 6 µg gDNA using the SureSelect Human All Exon V5 +UTR enrichment kit (Agilent). Bioinformatics analysis (Mendel disease) was performed by BGI. Exome sequencing coverage and variant calls were manually curated for 107 genes known to be linked to inherited arrhythmia syndromes or cardiomyopathies (Pua et al., 2016).

Generation of the guide RNAs (gRNAs)

Candidate gRNAs were identified around the intended mutation site using the bioinformatic website http://crispor.tefor.net, and gRNAs with higher specificity were favoured to minimise potential off-target sites (Table S3). The gRNAs were either synthesised as chimeric single gRNA (sgRNA) by *in vitro* transcription or purchased as crRNA and complexed with tracrRNA (both from IDT) prior to transfection.

For *in vitro* transcription, primers containing the T7 promoter, crRNA and a sequence homologous to the tracrRNA component present in the vector pSp-Cas9(BB)-2A-puro_v2 (Addgene #62988) were designed along with a primer complementary to the 3' end of the tracrRNA (Table S4). A 110bp amplicon was generated by PCR and purified using the QiaQuick PCR purification kit (Qiagen). The sgRNAs were transcribed using the HiScribe T7 kit (New England Biolabs). Following purification using the NucleoSpin RNA Clean-up XS kit (Macherey-Nagel), the integrity of the sgRNAs was confirmed by electrophoresis using a 10% TBE-urea precast gel (Bio-Rad).

The crRNAs and tracrRNA were resuspended in Nuclease-Free Duplex Buffer (IDT) to a final concentration of 100 µM. For complexing the RNAs, a 20 µM reaction was prepared by mixing 1 µl of each RNA with 3 µl of nuclease-free duplex buffer, incubating at 95˚C for 5 min, and leaving to cool to room temperature for at least 2 h before transfection.

Design of the single stranded oligonucleotides (ssODNs)

Approximately 125 bp asymmetric oligonucleotides were designed to contain the desired mutations and to have at least 36 nucleotides homologous to the genomic sequence 3' of the Cas9-induced double strand break (Table S3). The ssODNs were purchased as Ultramer DNA oligos (IDT) with standard desalting purification and resuspended in TE buffer to a final concentration of 100 µM.

CRISPR/Cas9 and ssODN transfection into hiPSCs

The gRNA, Cas9 protein (IDT or kindly provided by Niels Giessen (D'Astolfo et al., 2015)) and ssODN were transfected into the hiPSCs by either electroporation or lipofection. For electroporation, 1 µg Cas9 and 240 ng gRNA were mixed in a 0.5 ml sterile Protein LoBind tube (Eppendorf) and incubated at 25°C for 10 min to form the Cas9 ribonucleotide protein (RNP) complex. The hiPSCs were harvested and 1 x 10^5 cells mixed with the Cas9 RNP and 40 pmol ssODN in a 10 µl total reaction. Electroporation was performed using the Neon Transfection System (ThermoFisher), and the electroporated cells transferred immediately into a Laminin-521

coated well containing 500 µl StemFlex™ Medium with RevitaCell™ Supplement (1:100). Approximately 3 days later, the cells were harvested and expanded for subcloning.

For lipofection, MEF-maintained hiPSCs were transfected when 60–75% confluent. On the day of transfection, medium was refreshed 1 h before the procedure. Transfection reactions were prepared in a 0.5 ml sterile Protein LoBind tube by mixing 1.5 µg Cas9 with 380 ng of sgRNA, followed by the addition of Opti-MEM™ I Reduced Serum Medium (Gibco) to a final volume of 25 µl, and incubated as described above to form the Cas9 RNP complex. For co-delivery of the donor template, 4 pmol of ssODN was diluted in Opti-MEM™ I Reduced Serum Medium to a final volume of 25 µl. A lipofectamine mixture was prepared by diluting Lipofectamine® 2000 Transfection Reagent (Invitrogen) in Opti-MEM™ I Reduced Serum Medium (1:10). The Cas9 RNP, and ssODN solution were then combined with the diluted lipofectamine to a final volume of 100 µl and incubated at room temperature for 10 min before added to the hiPSCs. Approximately 6 h after transfection the media was refreshed, and at 72 h, the cells harvested and expanded for subcloning.

Targeting strategy to generate the KCNH2$^{PR/WT}$ hiPSC lines

Using the electroporation method, the KCNH2$^{PR/WT}$ hiPSC lines, LUMC0020iHERG-03 (KCNH2$^{PR/WT}$) and LUMC0020iHERG-04 (KCNH2$^{PR/WT}$ cl2), were created by introducing a heterozygous point mutation to substitute alanine to threonine at position 561 of KCNH2. A gRNA site 45 nucleotides 5' of the mutation site was targeted with a crRNA-tracrRNA complex, and the ssODN (ssODN_Pore) was designed to have a silent mutation within the PAM sequence to aide both colony screening and to prevent re-cutting of the modified locus by Cas9. For screening the resulting colonies, approximately 1 kB surrounding the target site was amplified by PCR and digested with *HaeII* to identify putative mono-allelic targeted clones. These were subsequently confirmed to be heterozygous for the mutation by Sanger sequencing.

Targeting strategy to generate the KCNH2$^{TL/WT}$ hiPSC lines

The KCNH2$^{TL/WT}$ hiPSC line (LUMC0020iHERG-01) was generated by introducing a heterozygous point mutation to substitute asparagine to isoleucine at position 996 of KCNH2. The target sequence for the sgRNA included the site to mutate, and the ssODN (ssODN Tail) was designed to include the desired nucleotide modification. For the KCNH2$^{TL/WT}$ cl2 hiPSC line (LUMC0020iHERG-02) a different sgRNA also covering the mutation site was transfected, along with both the ssODN Tail as well as a ssODN lacking the mutation but including two silent mutations. This additional ssODN functioned as competitor homology template to improve the frequency of obtaining a heterozygous clone. For screening the resulting colonies, approximately 1 kB surrounding the target site was amplified by PCR and digested with *BccI* to identify putative mono-allelic targeted clones. These were subsequently confirmed to be heterozygous for the mutation by Sanger sequencing.

Subcloning and PCR screening of the hiPSCs

Transfected hiPSCs were clonally isolated by single-cell deposition using a flow cytometer. Briefly, the cells were harvested using TrypLE™ Select Enzyme and filtered to remove cell clumps. If the transfected hiPSCs had been cultured on MEFs, prior to subcloning, the cells were stained with anti-MEF antibody (PE-conjugated; Miltenyi Biotec) to exclude these cells. All lines were stained with 4',6 Diamidino-2-Phenylindole (DAPI, Invitrogen) to exclude dead cells. A single hiPSC was deposited directly into each well of a 96-well plate in the same culture conditions as what the transfected hiPSCs had been maintained in. To assist with clonal recovery, the culture media also contained RevitaCell™ Supplement (1:100). Medium was initially changed three days after deposition and cells were maintained for ~2 weeks with medium changes every 3-4 days. Wells containing hiPSC colonies were dissociated with StemPro™ Accutase™ Cell Dissociation Reagent (Gibco) and duplicated across two 96-well plates. DNA for PCR screening and restriction fragment length polymorphism (RFLP) analysis was isolated from the cells in one plate with QuickExtract™ DNA Extraction Solution (Lucigen), while the cells in the replicate plate were cryopreserved.

Off-target analysis

Potential genomic off-targets for each gRNA were identified using http://crispor.tefor.net. The top 5 candidates with up to 4 base mismatches, were evaluated for the presence of Cas9-induced mutations (Table S2). The region around the off-target site was PCR amplified from both the targeted and KCNH2$^{WT/WT}$ hiPSC lines. The PCR amplicons were treated with Exonuclease I and Shrimp Alkaline Phosphatase (both New England Biolabs) and sequenced by Sanger sequencing to confirm the absence of off-target modifications.

Karyotyping

The genetically-modified hiPSC lines were karyotyped by G-banding. Chromosome spreads and analyses were performed by Cell Guidance Systems (UK) or by the Laboratory for Diagnostic Genome Analysis (Leiden University Medical Center). For each cell line, 20 metaphase spreads were examined with samples of sufficient quality to detect numerical and large structural abnormalities.

Allele-specific expression of *KCNH2*

Total RNA was isolated from hiPSC-CMs using the NucleoSpin® RNA Kit (Macherey-Nagel) according to manufacturer's instructions, with DNAse treatment performed using the DNA-free™ DNA Removal Kit (Ambion). RNA was reverse transcribed into cDNA using the iScript™ cDNA Synthesis Kit (Bio-Rad). Using primer pairs flanking each mutation (Table S4), PCR products generated from the *KCNH2* transcripts were cloned into the pMiniT™ 2.0 vector using the NEB® PCR Cloning Kit (New England Biolabs). For each *KCNH2* variant line at least 29 clones containing the transcript underwent Sanger sequencing.

Flow cytometric analysis

A single cell suspension of hiPSCs or hiPSC-CMs was obtained by dissociating the cells with TrypLE™ Select Enzyme and filtering the cell suspension. Cells were fixed and permeabilised using the Fix & Perm Cell Permeabilization Kit (Invitrogen) according manufacturer's instructions. For the hiPSC-CMs, the cells were first incubated with a Viobility™ 405/520 fixable dye (Miltenyi Biotec) prior to fixation for subsequent exclusion of dead cells. The hiPSCs were incubated with the conjugated antibodies OCT4-BV421 (1:25, BD #565644), Sox2-A488 (1:200, eBioscience, #53-9811-80), Tra-1-60-PE (1:20, Miltenyi Biotec, #130-100-347) and SSEA4-PE-vio770 (1:100, Miltenyi Biotec, #130-105-082), while the hiPSC-CMs were incubated with cTnT-Vioblue (1:11, Miltenyi Biotec, #130-106-686) and MLC2v-PE (1:11, Miltenyi Biotec, #130-106-183). All antibodies were diluted in permeabilization medium (medium B; Invitrogen). Samples were measured using a MACSQuant VYB flow cytometer (Miltenyi Biotec), and data analysed using FlowJo software (FlowJo).

Immunofluorescence analysis

For hiPSCs, cells were fixed in 2% paraformaldehyde for 30 min, permeabilized with phosphate buffer saline (PBS)/0.1% Triton X-100 (Sigma-Aldrich) and blocked with 1% bovine serum albumin (BSA, Sigma-Aldrich) and 0.05% Tween (Merck) in PBS. Samples were incubated overnight at 4°C with antibodies specific for NANOG (1:200, R&D, #AF1997) and SSEA4 (1:200, Santa Cruz Biotechnology, #SC59368). These primary antibodies were detected with Alexa Fluor 555- (1:500, ThermoFisher, #A21432) and Alexa Fluor 488- (1:500, Life Technologies, #A-21202) conjugated antibodies, respectively. Nuclei were visualised with DAPI (0.3 μM) and images captured using an EVOS FL Auto 2 Cell Imaging System (ThermoFisher).

The hiPSC-CMs plated on glass coverslips were fixed using the Inside Stain Kit (Miltenyi Biotec) according to manufacturer's instructions. The fixed cells were incubated with α-actinin (1:250, Sigma-Aldrich, #A7811) and myosin heavy chain (1:50, Miltenyi Biotec, #130-112-757) antibodies, followed by Alexa Fluor 594- (1:250, ThermoFisher, #A-21203) and Vio515- (1:100, Miltenyi Biotec, #130-112-760) conjugated secondary antibodies. Nuclei were stained with DAPI and images captured using a confocal laser scanning microscope SP8 (Leica) at 40x magnification.

Western Blot

Protein isolation and western blot in hiPSC-CMs were performed as previously described (Bellin et al., 2013). Briefly, samples were lysed in RIPA Lysis and Extraction Buffer and total protein measured using the Pierce™ BCA Protein Assay (both Thermo Fisher) according to manufacturer's protocols. The protein samples (40 μg) were loaded on an 8% polyacrylamide gel, and transfer performed using standard protocols (Kurien and Scofield, 2006). The membrane was incubated with the primary antibodies hERG1a (1:1000, Cell Signalling Technology, #12889) and β-actin (1:2000, Abcam, #8227 or 1:2000, Merck, #MAB1501R), followed by appropriate HRP-conjugated secondary antibodies (1:10000, Cell Signalling Technology, #7074 and #7076S), and the chemiluminescence signal detected using WesternBright Quantum HRP substrate (Isogen Life Science).

Patch clamp data acquisition

Electrophysiological recordings were performed on single hiPSC-CMs. For action potential (AP) and rapid delayed rectifier potassium current (I_{Kr}) measurements, spontaneously contracting cardiomyocytes were selected in Tyrode's solution containing (in mM): NaCl 140, KCl 5.4, $CaCl_2$ 1.8, $MgCl_2$ 1.0, glucose 5.5, and HEPES 5.0; pH 7.4 (NaOH). I_{Kr} and APs were measured with the ruptured or perforated patch-clamp technique, respectively, using an Axopatch 200B amplifier (Molecular Devices) at 36 ± 0.2°C. Voltage control and data acquisition of I_{Kr} and APs were performed with pClamp 10.4/Clampfit (Axon Instruments) and custom-made software, respectively. Potentials were corrected for the calculated liquid junction potentials (Barry and Lynch, 1991), which was 15 mV for both AP and I_{Kr} measurements. Low-resistance patch pipettes (2-3 MΩ; Borosilicate glass capillaries, Harvard Apparatus) were used, and for I_{Kr} measurements, series resistance (Rs) was compensated for ≥80%. I_{Kr} and APs were low-pass filtered (cut-off frequency of 5 kHz and 2 kHz, respectively), and digitised (55 kHz and 40 kHz, respectively).

Voltage-clamp experiments

I_{Kr} was measured using 4 s hyper- and depolarising pulses from a holding potential of -40 mV, at a cycle length of 10 s. The extracellular solution was Tyrode's solution, while the pipette solution contained (in mM): K-gluconate 125, KCl 20, K_2-ATP 5, HEPES 10, and EGTA 10; pH 7.2 (KOH). The L-type Ca^{2+} current was blocked by adding 5 µM nifedipine (Sigma) to the extracellular solution. I_{Kr} was measured as an E-4031-sensitive current by subtracting the current recorded before and after application of 5 µM E-4031 (Tocris). I_{Kr} densities (steady-state and tail) were calculated by dividing current amplitude (pA), measured at the depolarizing voltage steps and upon stepping back to the holding potential, by cell membrane capacitance (pF). Cell membrane capacitance was measured by dividing the decay time constant of the capacitive transient in response to 5 mV hyperpolarising steps from -40 mV, by the Rs.

Current-clamp experiments

APs were measured in Tyrode's solution, while the pipette solution contained (in mM): K-gluconate 125; KCl 20; NaCl 5.0; amphotericin-B 0.44, and HEPES 10; pH 7.2 (KOH). Because hiPSC-CMs typically have a small or even absent inward rectifying potassium current (I_{K1}), the cells have a depolarised resting membrane potential (RMP) and are frequently spontaneously active. We therefore injected an *in silico* 2 pA/pF I_{K1} with kinetics of $Kir_{2.1}$ channels through dynamic clamp, resulting in quiescent hiPSC-CMs with a RMP of less than -75 mV. APs were elicited at 0.2 Hz, 0.5 Hz, 1 Hz, 2 Hz, 3 Hz and 4 Hz by 3 ms, ~1.2x threshold current pulses through the patch pipette. The RMP, upstroke velocity, AP amplitude, AP plateau amplitude and AP duration at 20%, 50% and 90% repolarisation were analysed. Parameters from 13 consecutive APs were averaged.

MEA electrophysiology

Multi-electrode array (MEA) experiments to record spontaneous electrical activity of hiPSC-CMs were done using 60-electrode MEA chips or multi-well MEA plates (both Multichannel Systems). The MEAs were coated with human fibronectin (40 µg/ml, Alfa Aesar) for 1 h at 37°C, and hiPSC-CMs seeded directly on the electrodes at a density of 1.5 x 10^5 cells/cm^2. Medium was changed at least 1 h before baseline recordings. All measurements were made at 37°C in Medium C and at least 15 min after placing the MEA in the recording system. Extracellular recordings for the 60-electrode MEA chips were performed using a MEA1060INV amplifier (Multichannel systems) as previously described (Sala et al., 2017). Recordings for the multi-well MEA plates were made using a Multi-well-MEA system (Multichannel systems) at a sampling rate of 20 kHz and high- and low pass filters of 3500 Hz and 1 Hz respectively.

For evaluating E-4031-induced effects on the hiPSC-CMs, sequential addition of increasing concentrations of E-4031 was performed. E-4031 (Tocris) was dissolved in DMSO (Sigma-Aldrich) at 10 mM, with serial dilutions made in Medium C. The final concentration of the drug was achieved by stepwise removal of medium and addition of the same volume of diluted E-4031 to the well. No more than 7% of the total volume was replaced. The response to each E-4031 concentration was recorded for 1 min after an incubation period of 1 min. The maximum amount of DMSO present in the culture medium was 0.3% at the highest concentration of E-4031. Controls indicated that this percentage of DMSO did not alter the field potential duration (FPD) of the hiPSC-CMs or trigger arrhythmic events.

MEA traces were analysed with the investigator blinded, with FPD and peak-to-peak intervals quantified over the whole recording time as previously described (Sala et al., 2017). Good quality traces were defined by the presence of a clear peak corresponding to the Na^+ influx and membrane depolarization, a clear repolarization phase corresponding to K^+ efflux, and a high signal-to-noise ratio (SNR). The SNR was very high for the vast majority of recordings. However if the trace was noisy, filtering was performed prior to analysis. Fridericia's formula for frequency correction was used to correct for FPD dependence on beating rate (cFPD)

$$\frac{FPD}{\sqrt[3]{RR}}$$

where FPD is in ms and RR in s. Compound responses of the hiPSC-CMs were normalised to their baseline measurement. Arrhythmia-like events were classified and scored based on the following categories: no arrhythmia (0); variable beat period (1); abnormal depolarisations (2); fibrillation (3); quiescent (4). Fibrillation was defined as a severe reduction in the FP amplitude, leading to an almost complete loss the Na^+ peak, concomitant with the presence of uncoordinated electrical activity. Quiescent cells were counted as arrhythmic cells with the highest score based on the I_{Kr}-blocking effects of E-4031. Increasing amounts of E-4031 prolonged the FPD which resulted at high concentrations in a failure of membrane potential to properly repolarise, and consequently an inability to evoke APs at such depolarised diastolic potentials.

For evaluating lumacaftor-induced effects on hiPSC-CMs, the cells were seeded at a density of 4.4 x 10^5 cells/cm^2 and cultured on 96-well MEA plates (M768-tMEA-96OPT, Axion Biosystems) as described above. 7 days after seeding, the hiPSC-CMs were treated with 5 µM (final concentration) lumacaftor (VX-809; MedChemExpress) or vehicle control (final concentration 0.1% DMSO) for 8 days with the compound refreshed every 48 hrs. Extracellular recordings were performed using a Maestro Pro system (Axion Biosystems) before addition of the compounds and after 8 days of treatment. MEA traces were analysed using the platform-based Cardiac Analysis Tool to determine the FPD and beat period. Frequency correction was not used for lumacaftor responses since this overestimated the rate-dependency of the FPD. Compound responses of the hiPSC-CMs were normalised to their baseline measurement.

Optical evaluation using the Triple Transient Measurement (TTM) system
The hiPSC-CMs were plated at between 3 – 5.5 x 10^4 cells per well in black glass-bottom 96-well plates (Greiner), pre-coated with 1:100 Matrigel in DMEM/F-12 (Gibco). Medium was replaced the next day and thereafter every 2-3 days with Pluricyte Cardiomyocyte Medium (PCM; NCardia). Analysis was performed 5-7 days after plating. Cells were incubated for 20 min at 37°C (protected from light) with a voltage sensitive dye (6 µM ANNINE-6plus; Sensitive Farbstoffe), a Ca^{2+} sensitive dye (6 µM Rhod 3; ThermoFisher) and a cell membrane labelling dye (5 µM CellMask Deep Red; ThermoFisher) in PCM for measuring electrical activity, Ca^{2+} flux and contraction, respectively. The dye medium was then removed and PCM added, with cells left to recover for 10-15 min at 37°C protected from light before analysis.

Recording and data processing were performed using a bespoke fast optical switch microscopy system and algorithms developed in-house (van Meer et al., 2019). Briefly, cells were paced at 1.2 Hz using 10 ms pulses of 14 V with a pair of field stimulation electrodes placed in the culture medium. An Eclipse Ti microscope (Nikon) was fitted with a high-power three LED system (Mightex; 470 nm, 560 nm and 656 nm) including collimators and excitation filters (Semrock; 470±14 nm, 544±12 nm, 650±7.5 nm). A 63x oil immersion objective was used, together with an image intensifier (Photonis) and a high-speed camera (Optronis). Plates were maintained in an environmental chamber at 5% CO_2 and 37°C. Three fields of view were measured per well, with recordings of all three fluorescence channels performed at 1000 frames/s for 7 s using fast optical switching of LEDs (1 ms per channel). The three channel signals were then separated and peaks averaged. The 90% durations were then calculated. For voltage this was taken as the top of the peak to 90% of repolarisation, whereas for Ca^{2+} and contraction this was taken from 10% above baseline in the upstroke to 90% of the decay. Five wells were analysed per differentiation of hiPSC-CMs, with three independent differentiations used to generate the mean data per line (n = 15 wells, 45 fields of view).

References

Barry, P.H., and Lynch, J.W. (1991). Liquid junction potentials and small cell effects in patch-clamp analysis. J. Membr. Biol. *121*, 101–117.

Bellin, M., Casini, S., Davis, R.P., D'Aniello, C., Haas, J., Ward-van Oostwaard, D., Tertoolen, L.G.J., Jung, C.B., Elliott, D.A., Welling, A., et al. (2013). Isogenic human pluripotent stem cell pairs reveal the role of a KCNH2 mutation in long-QT syndrome. EMBO J. *32*, 3161–3175.

van den Berg, C.W., Elliott, D.A., Braam, S.R., Mummery, C.L., and Davis, R.P. (2014). Differentiation of Human Pluripotent Stem Cells to Cardiomyocytes Under Defined Conditions. Methods Mol. Biol. *1353*, 163–180.

D'Astolfo, D.S., Pagliero, R.J., Pras, A., Karthaus, W.R., Clevers, H., Prasad, V., Lebbink, R.J., Rehmann, H., and Geijsen, N. (2015). Efficient Intracellular Delivery of Native Proteins. Cell *161*, 674–690.

Davis, R.P., Grandela, C., Sourris, K., Hatzistavrou, T., Dottori, M., Elefanty, A.G., Stanley, E.G., and Costa, M. (2009). Generation of Human Embryonic Stem Cell Reporter Knock-In Lines by Homologous Recombination. Curr Protoc Stem Cell Biol Chap.5 Unit 5B 1 1 1-34.

Kurien, B., and Scofield, R. (2006). Western blotting. Methods *38*, 283–293.

van Meer, B.J., Krotenberg, A., Sala, L., Davis, R.P., Eschenhagen, T., Denning, C., Tertoolen, L.G.J., and Mummery, C.L. (2019). Simultaneous measurement of excitation-contraction coupling parameters identifies mechanisms underlying contractile responses of hiPSC-derived cardiomyocytes. Nat. Commun. *10*, 4325.

Pua, C.J., Bhalshankar, J., Miao, K., Walsh, R., John, S., Lim, S.Q., Chow, K., Buchan, R., Soh, B.Y., Lio, P.M., et al. (2016). Development of a Comprehensive Sequencing Assay for Inherited Cardiac Condition Genes. J. Cardiovasc. Transl. Res. *9*, 3–11.

Sala, L., Ward-van Oostwaard, D., Tertoolen, L.G.J., Mummery, C.L., and Bellin, M. (2017). Electrophysiological Analysis of human Pluripotent Stem Cell-derived Cardiomyocytes (hPSC-CMs) Using Multi-electrode Arrays (MEAs). J. Vis. Exp. *123*, e55587.
