# Supplementary material for: Isogenic Sets of hiPSC-CMs Harboring Distinct KCNH2 Mutations Differ Functionally and in Susceptibility to Drug-Induced Arrhythmias
Source: Stem Cell Reports. 2020 Nov 10;15(5):1127–39. doi: 10.1016/j.stemcr.2020.10.005 (PMC7664051; doi:10.1016/j.stemcr.2020.10.005)
Supplement: Document S2. Article plus Supplemental Information [file mmc3.pdf]

Stem Cell Reports
Article
OPEN ACCESS

Isogenic Sets of hiPSC-CMs Harboring Distinct *KCNH2* Mutations Differ Functionally and in Susceptibility to Drug-Induced Arrhythmias

Karina O. Brandão,[1] Lettine van den Brink,[1] Duncan C. Miller,[1] Catarina Grandela,[1] Berend J. van Meer,[1] Mervyn P.H. Mol,[1] Tessa de Korte,[1] Leon G.J. Tertoolen,[1] Christine L. Mummery,[1] Luca Sala,[2] Arie O. Verkerk,[3,4] and Richard P. Davis[1,*]
[1]Department of Anatomy and Embryology, Leiden University Medical Center, 2300RC Leiden, The Netherlands
[2]Istituto Auxologico Italiano, IRCCS, Laboratory of Cardiovascular Genetics, 20095 Milan, Italy
[3]Department of Medical Biology, Amsterdam UMC, University of Amsterdam, 1105AZ Amsterdam, The Netherlands
[4]Department of Experimental Cardiology, Amsterdam UMC, University of Amsterdam, 1105AZ Amsterdam, The Netherlands
*Correspondence: r.p.davis@lumc.nl
https://doi.org/10.1016/j.stemcr.2020.10.005

SUMMARY

Mutations in *KCNH2* can lead to long QT syndrome type 2. Variable disease manifestation observed with this channelopathy is associated with the location and type of mutation within the protein, complicating efforts to predict patient risk. Here, we demonstrated phenotypic differences in cardiomyocytes derived from isogenic human induced pluripotent stem cells (hiPSC-CMs) genetically edited to harbor mutations either within the pore or tail region of the ion channel. Electrophysiological analysis confirmed that the mutations prolonged repolarization of the hiPSC-CMs, with differences between the mutations evident in monolayer cultures. Blocking the hERG channel revealed that the pore-loop mutation conferred greater susceptibility to arrhythmic events. These findings showed that subtle phenotypic differences related to *KCNH2* mutations could be captured by hiPSC-CMs under genetically matched conditions. Moreover, the results support hiPSC-CMs as strong candidates for evaluating the underlying severity of individual *KCNH2* mutations in humans, which could facilitate patient risk stratification.

INTRODUCTION

Congenital long QT syndrome (LQTS) is a genetic disease with an estimated prevalence of ~1:2,000 individuals. It is characterized by a prolonged QT interval on an electrocardiogram that can lead to sudden cardiac death, particularly in young people (George, 2013). Although the identification of genes associated with LQTS has dramatically improved our understanding of the disease, clinical management remains complicated by the variability in disease expressivity and penetrance among mutation carriers which range from lifelong asymptomatic to experiencing life-threatening arrhythmias (Giudicessi and Ackerman, 2013). While environmental factors are contributors to this clinical heterogeneity (Bezzina et al., 2015), genetics also plays a major role through both the primary genetic mutation and the presence of additional genetic variants that modify the disease outcome (Napolitano et al., 2018; Schwartz et al., 2018).

Type 2 LQTS (LQT2) is the second most prevalent form of congenital LQTS and is due to mutations in *KCNH2*, which encodes the α subunit of the Kv11.1 (hERG) channel responsible for conducting the rapid delayed rectifier potassium current (I_{Kr}) in cardiomyocytes (Curran et al., 1995). Several studies have demonstrated that the location of the mutation within this ion channel is an important determinant of arrhythmic risk in LQT2 patients, with patients harboring mutations in the pore-loop region at higher risk of cardiac events than those with mutations located in other regions (Migdalovich et al., 2011; Moss et al., 2002; Nagaoka et al., 2008; Shimizu et al., 2009). Furthermore, mutations that result in a dominant-negative effect, in which the function of wild-type hERG is reduced or eliminated, also produce higher adverse event rates (Migdalovich et al., 2011). However, mutations within the pore-loop region can, in some instances, result in less severe outcomes (Zhao et al., 2009), highlighting the need for *in vitro* models to accurately classify these rare variants and for gaining mechanistic insights into their contribution to disease phenotypes.

Cardiomyocytes derived from human induced pluripotent stem cells (hiPSCs) are now well established as models for LQT2 (Bellin et al., 2013; Itzhaki et al., 2011; Matsa et al., 2011; Mehta et al., 2018). Indeed, a number of hiPSC lines have been derived from both symptomatic and asymptomatic patients with mutations in various regions of hERG (Brandão et al., 2017). However, as these lines are from different individuals, they harbor additional genetic variants that may functionally influence the disease phenotype observed and limit the utility of hiPSC-derived cardiomyocytes (hiPSC-CMs) for broad intragenotype risk stratification.

To create a tailored model to study the genetic etiology of LQT2, we generated a set of isogenic hiPSC lines that possess heterozygous mutations within the pore-loop domain (KCNH2-A561T) or in the cytoplasmic tail (KCNH2-N996I) of hERG by genetically modifying a control hiPSC line. Molecular and functional comparisons of

This is an open access article under the CC BY license (http://creativecommons.org/licenses/by/4.0/).

these edited lines confirmed not only that the *KCNH2* variant hiPSC-CMs phenocopied the key features of LQT2 but also that differences due to the mutation were identified in the cell lines. This included dissimilarities in the mechanism underlying the hERG channel defect caused by these mutations, as well as a more prolonged repolarization observed in hiPSC-CMs with the pore mutation when measured as a syncytium. Furthermore, when these hiPSC-CMs were exposed to E-4031, a hERG channel blocker, they were more susceptible to proarrhythmic effects compared with either the hiPSC-CMs with the tail mutation or the unedited control. Our findings highlight the potential of hiPSC-CMs to reveal the inherent severity of individual *KCNH2* mutations when using genetically matched lines, and also further advance hiPSC-CMs as models for not only predicting risk but also assisting in the stratification of patients.

RESULTS

Generation and Characterization of an Isogenic Set of KCNH2 Variant hiPSC Lines

A limitation of hiPSC lines derived from unrelated patients with different LQT2-causing mutations is the inability to compare the resulting hiPSC-CMs under genetically matched conditions. Therefore, to detect phenotypic differences between individual LQT2-causing *KCNH2* mutations, we elected to genetically introduce these variants into a well-characterized hiPSC line derived from a healthy individual (KCNH2$^{WT/WT}$) (Zhang et al., 2014). Furthermore, we confirmed that this cell line did not carry any known disease-causing mutations by performing whole-exome sequencing and examining a panel of 107 genes known to be linked to inherited arrhythmia syndromes or cardiomyopathies (Pua et al., 2016) (Table S1). All coding sequence variants identified were predicted to be benign due to their frequency in the general population being $\geq$1%. The only exception was a rare variant identified in *DOLK*, the gene encoding dolichol kinase. Homozygous mutations in this gene can lead to multi-systemic glycosylation disorders, including dilated cardiomyopathy, with individuals typically not surviving to adulthood (Lefeber et al., 2011). However, the variant identified in the KCNH2$^{WT/WT}$ line is unlikely to be pathogenic as the hiPSCs were derived from a healthy 51-year-old female and were heterozygous for the *DOLK* variant.

We then used a CRISPR-Cas9-mediated gene editing strategy to generate an isogenic set of hiPSC lines harboring a missense variant either within the pore-loop domain (NM_000238.3:c.1681G > A, NP_000229.1:p.Ala561Thr) or cytoplasmic tail region (NM_000238.3:c.2987A > T, NP_000229.1:p.Asp996Iso) of *KCNH2* (Figure 1). Restriction fragment length polymorphism analysis identified clones that appeared to be genetically modified and these were subsequently confirmed by Sanger sequencing to be either heterozygous for the KCNH2-A561T (KCNH2$^{PR/WT}$; Figure 1E) or the KCNH2-N996I (KCNH2$^{TL/WT}$; Figure 1F) variant. For each mutation, a second independent isogenic heterozygous clone (KCNH2$^{PR/WT}$ cl2 and KCNH2$^{TL/WT}$ cl2) was also selected for further characterization (Figures S1A–S1C). All clones were assessed by Sanger sequencing for potential off-target modifications due to the CRISPR-Cas9 transfection, with no insertions or deletions detected at any of the genomic loci examined (Table S2). Furthermore, G-band karyotyping of these clones indicated that the lines were karyotypically normal, and the undifferentiated hPSCs expressed the stem cell markers SOX2, OCT-4, NANOG, SSEA4, and TRA-1-60 (Figures S1D–S1F).

Distinct hERG Mutations Result in Differing Channel Functionality

A monolayer-based differentiation protocol was used to differentiate the KCNH2 variant and KCNH2$^{WT/WT}$ hiPSC lines to cardiomyocytes. Flow cytometric analysis for the pan-cardiomyocyte marker cardiac troponin T (cTnT) indicated that all lines, except KCNH2$^{PR/WT}$ cl2, differentiated with similar efficiencies, with on average ~70%–80% of the cells being cTnT^{+} (Figures 2A, 2B, S2A, and S2B). The hiPSC-CMs (cTnT^{+} cells) were also characterized for the expression of the ventricular cardiomyocyte marker, MLC2v. On average, the proportion of hiPSC-CMs that were ventricular was between 45% and 64% including for the KCNH2$^{PR/WT}$ cl2 line (Figures 2A, 2B, S2A, and S2B). The hiPSC-CMs also displayed characteristic sarcomeric structures that were positive for α-actinin and myosin heavy chain (Figure 2C).

Both KCNH2$^{PR/WT}$ and KCNH2$^{TL/WT}$ hiPSC-CMs expressed equal fractions of mutant and wild-type *KCNH2* transcripts (Figure 2D), confirming that the introduced mutations did not disrupt biallelic expression of the gene and suggesting that any differences observed between the cell lines would be due to dysfunction of hERG. Western blot analysis (Figure 2E) for hERG identified two protein bands—the fully glycosylated mature form (155 kDa) and the core-glycosylated precursor form (135 kDa) (Zhou et al., 1998a, 1998b). Although there was no significant difference in the total amount of hERG present between the three lines (Figure 2F), the mature form (155 kDa) was predominantly present in KCNH2$^{WT/WT}$ hiPSC-CMs, while in both KCNH2$^{PR/WT}$ and KCNH2$^{TL/WT}$ hiPSC-CMs, higher expression of the precursor form (135 kDa) was detected (Figures S2C and S2D). This is in agreement with previous studies that have reported these mutations as affecting glycosylation of hERG, leading to impaired protein trafficking (Bellin et al., 2013; Matsa et al., 2014).

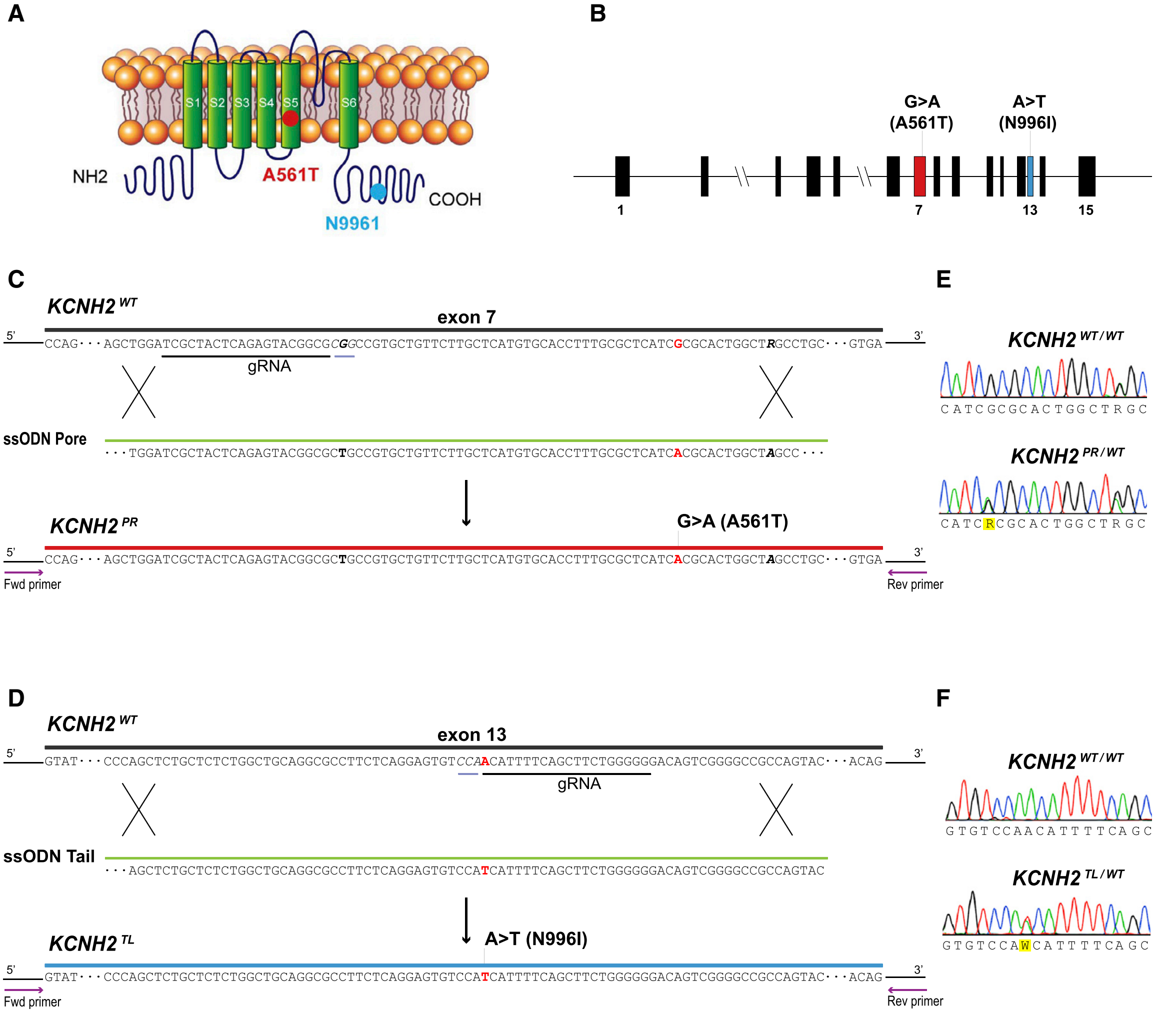

Figure 1. Generation of Isogenic hiPSC Lines with *KCNH2* Mutations

(A) Structure of the potassium ion channel hERG encoded by *KCNH2* indicating the introduced mutations (KCNH2-A561T, red dot; KCNH2-N996I, blue dot).

(B) Structure of the *KCNH2* genomic locus highlighting the exons modified to generate the KCNH2 variant hiPSC lines.

(C and D) Schematic outlining strategy to introduce the KCNH2-A561T mutation ($KCNH2^{PR}$) (C) or the KCNH2-N996I mutation ($KCNH2^{TL}$) (D) by homologous recombination into a *KCNH2* wild-type ($KCNH2^{WT}$) sequence. The gRNA and their corresponding protospacer adjacent motif sequences are underlined in black and gray, respectively. Part of the ssODN sequences to introduce the A561T (ssODN Pore) and N996I (ssODN Tail) mutations are shown. Nucleotides modified to introduce the mutations are indicated in red, silent mutations and SNPs used to assist with the targeting and screening are bolded in black. Arrows represent the PCR primers used to identify targeted clones.

(E and F) Sequence analysis of the PCR-amplified genomic DNA showing heterozygous introduction of NM_000238.3:c.G1681A (E) and NM_000238.3:c.A2987T (F).

See also Figure S1.

hERG-trafficking efficiency, calculated as the ratio of fully glycosylated hERG over total hERG present (Peterson et al., 2012), indicated a significant reduction in both KCNH2 variant lines relative to the $KCNH2^{WT/WT}$ line ($KCNH2^{PR/WT}$: 55%; $KCNH2^{TL/WT}$: 19%; Figure 2G).

Finally, we determined the effect of these *KCNH2* mutations on I_{Kr}. Representative examples of current traces in individual hiPSC-CMs from both KCNH2 variant and $KCNH2^{WT/WT}$ hiPSC-CMs are shown in Figure 2H, with I_{Kr} measured as an E-4031-sensitive current. The I_{Kr} tail and steady-state current densities were significantly lower in both $KCNH2^{PR/WT}$ and $KCNH2^{TL/WT}$ hiPSC-CMs compared with the $KCNH2^{WT/WT}$ hiPSC-CMs (Figures 2I and 2J). For example, after a voltage step to -10 mV, the I_{Kr} tail current density was 39% smaller in the $KCNH2^{TL/WT}$ hiPSC-CMs and 67% smaller in the $KCNH2^{PR/WT}$

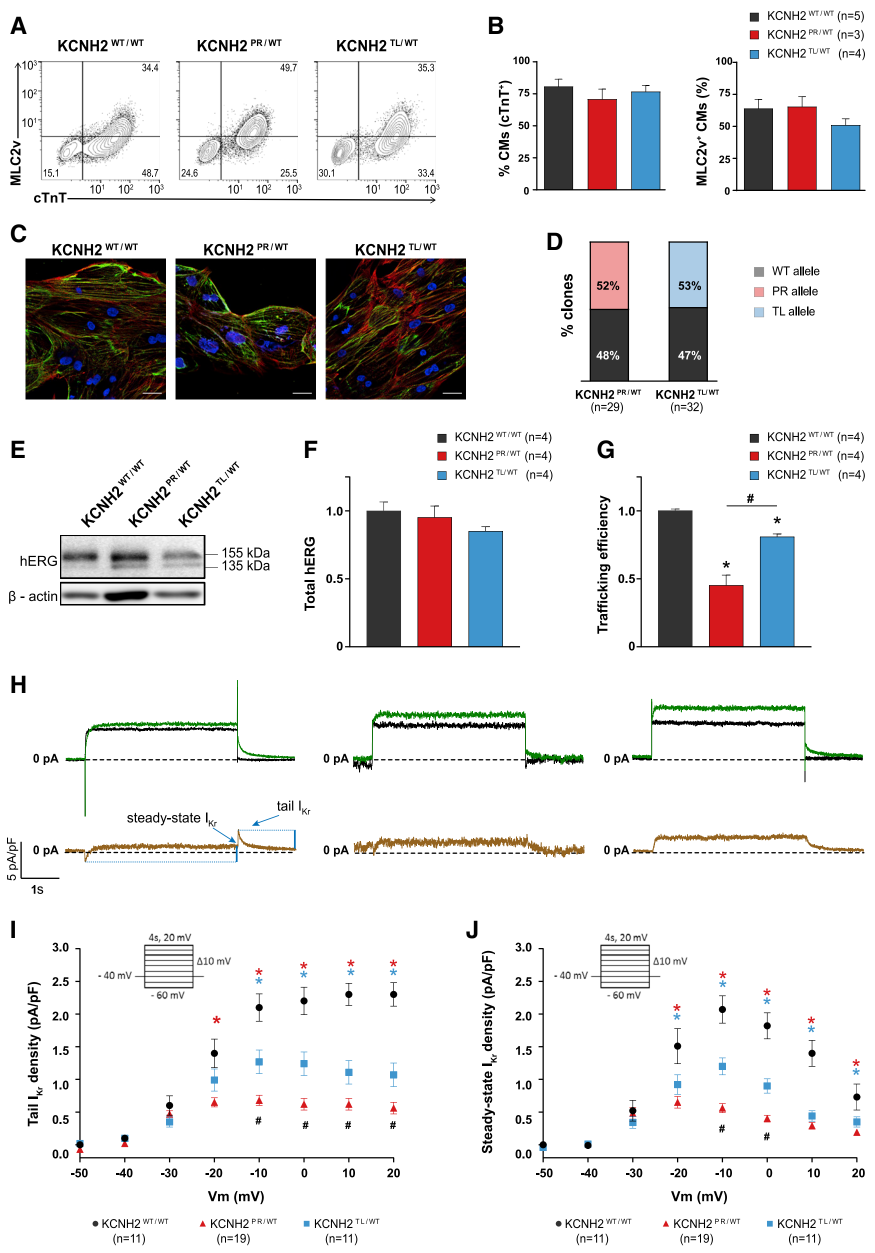

Figure 2. Evaluation of hERG Channel Function in Differentiation Day 21 + 7 KCNH2$^{WT/WT}$, KCNH2$^{PR/WT}$, and KCNH2$^{TL/WT}$ hiPSC-CMs

(A) Representative flow cytometry plots of hiPSC-CMs for expression of cTnT and MLC2v in the indicated lines. Values inside the plots are the percentage of cells within the gated region.

(B) Overall cardiac differentiation efficiency of the three hiPSC lines, showing the average percentage of hiPSC-CMs (cTnT^{+}) (left graph), and the proportion of ventricular-like (MLC2v^{+}) cardiomyocytes within the hiPSC-CM population (right graph). Values (n) refer to the number of independent differentiations analyzed.

(C) Immunofluorescence images of the cardiac sarcomeric proteins α-actinin (red) and myosin heavy chain isoforms α and β (green) in the indicated lines. Nuclei (blue) were stained with DAPI. Scale bars, 25 μm.

(D) Percentage of *KCNH2* mRNA in the KCNH2$^{PR/WT}$ and KCNH2$^{TL/WT}$ hiPSC-CMs transcribed from the wild-type (WT) or mutated (PR, TL) alleles. Values (n) refer to the number of independent clones sequenced.

(E) Western blot analysis of hERG in the indicated lines. Bands corresponding to core and fully glycosylated hERG (135 and 155 kDa, respectively) are marked. β-Actin was used as a loading control.

(F and G) Densitometric quantification of western blots in (E) and Figure S2C for total hERG protein (155 +135 kDa; relative to KCNH2$^{WT/WT}$) (F), and the ratio of fully glycosylated over total hERG protein (trafficking efficiency) (G). Data normalized to β-actin. *statistical significance to KCNH2$^{WT/WT}$ ($p < 0.05$); #statistical significance between KCNH2$^{PR/WT}$ and KCNH2$^{TL/WT}$ ($p < 0.001$); one-way ANOVA with Tukey's multiple comparisons test. Values (n) refer to the number of independent differentiations analyzed.

(H) Top panels show representative current traces evoked by a 4-s voltage step from −40 to 0 mV before (green lines) and after (black lines) application of 5 μM E-4031. Bottom panels present the E-4031-sensitive current (brown lines). Arrows indicate the sections of the traces analyzed to determine the tail and steady-state I_{Kr} current densities.

(I and J) Average current-voltage relationships for tail (I) and steady-state (J) I_{Kr} current densities in the indicated hiPSC-CMs. All CMs activated upon depolarization reached a maximum steady-state current at −10 and 0 mV, which decreased at more positive potentials due to the onset of inactivation. Inset: voltage protocol; *statistical significance to KCNH2$^{WT/WT}$ (tail I_{Kr}, $p < 0.0001$; steady-state I_{Kr}, $p < 0.01$); #statistical significance between KCNH2$^{PR/WT}$ and KCNH2$^{TL/WT}$ (tail I_{Kr}: −10 mV, 0 mV, $p < 0.001$; 10 mV, 20 mV, $p < 0.01$; steady-state I_{Kr}: −10 mV, $p < 0.0001$, 0 mV, $p < 0.01$); two-way ANOVA with Tukey's multiple comparisons test. Values (n) refer to the number of individual hiPSC-CMs analyzed.

Error bars represent SEM.

See also Figure S2.

hiPSC-CMs. A similar fold reduction was also observed for the steady-state current at −10 mV (KCNH2$^{PR/WT}$: 73%; KCNH2$^{TL/WT}$: 42%). Taken together, these findings indicate that both *KCNH2* variants result in impaired glycosylation of the hERG protein but, in terms of the availability of functional channels, the KCNH2$^{TL/WT}$ mutation leads to haploinsufficiency, while the KCNH2$^{PR/WT}$ mutation causes a dominant-negative phenotype.

We also endeavored to demonstrate that reduced trafficking of hERG might contribute to the decrease in I_{Kr} density. To investigate this, we treated the hiPSC-CMs with lumacaftor, a drug shown previously to rescue hERG-trafficking defects and lead to a shortening of the field potential duration (FPD) (Mehta et al., 2018). While we also observed a significant shortening of the FPD in the KCNH2 variant lines upon treatment with 5 μM lumacaftor for 8 days (Figure S2E), a similar effect with the vehicle control (0.1% DMSO) was detected. DMSO is known to be able to stabilize protein conformation during their maturation and rescue trafficking defects (Thomas, 2003). Therefore, it is possible that the chaperone properties of this chemical as well as lumacaftor contributed to the FPD reduction we observed.

The Dominant-Negative *KCNH2* Mutation Causes a More Severe Electrical Phenotype

To determine whether the differences in I_{Kr} density between the two KCNH2 variants was also reflected in the action potential duration (APD), action potentials (APs) from individual hiPSC-CMs were recorded (Figure 3A). All cells measured had AP plateau amplitude values >85 mV (Figure S3A), indicating that the hiPSC-CMs analyzed were all ventricular-like (Devalla et al., 2015; Veerman et al., 2016). The APD at 50% and 90% repolarization (APD_{50} and APD_{90}, respectively) were significantly prolonged for both KCNH2 variant lines (KCNH2$^{PR/WT}$: 185 ± 17 ms [APD_{50}], 243 ± 19 ms [APD_{90}]; KCNH2$^{TL/WT}$: 198 ± 16 ms [APD_{50}], 254 ± 18 ms [APD_{90}]) compared with the KCNH2$^{WT/WT}$ hiPSC-CMs (131 ± 11 ms [APD_{50}], 151 ± 11 ms [APD_{90}]), although there was no significant difference between the two KCNH2 variant lines (Figure 3B). At both slower and faster pacing frequencies (0.2–4 Hz), the differences in APD_{90} between the KCNH2 variant and wild-type lines remained, without any significant differences between the KCNH2$^{PR/WT}$ and KCNH2$^{TL/WT}$ hiPSC-CMs (Figure S3B and S3C). In addition, arrhythmogenic activity, as evidenced by the presence of early after depolarizations (EADs) during 0.2 Hz stimulation, were only detected in the KCNH2 variant lines with no difference in the frequency of EADs between the two lines (KCNH2$^{PR/WT}$: 18.2%; KCNH2$^{TL/WT}$: 17.6%) (Figures 3C and 3D). No significant differences in AP amplitude and resting membrane potential (RMP) were observed between any of the lines, and while upstroke velocity (V_{max}) appeared faster in the KCNH2$^{PR/WT}$ hiPSC-CMs, this was not significant (p = 0.07) (Figure 3B).

Next, we investigated whether differences could be detected between the KCNH2$^{PR/WT}$ and KCNH2$^{TL/WT}$ hiPSC-CMs if measured in a syncytium. Figure 3E shows representative field potential (FP) recordings of the hiPSC-CMs obtained from a multi-electrode arrays (MEA) platform. Differences in beating frequency were observed, with the KCNH2$^{PR/WT}$ hiPSC-CMs showing a significantly shorter beat period than the KCNH2$^{WT/WT}$ and KCNH2$^{TL/WT}$ hiPSC-CMs (1,985 ± 43 versus 3,515 ± 126 and 3,342 ± 102 ms, respectively). Therefore, the FPD was corrected (cFPD) for the beat rate according to Fridericia's formula (Figure 3F). Mirroring the APD differences observed in single hiPSC-CMs, the cFPD was prolonged in KCNH2$^{PR/WT}$ and KCNH2$^{TL/WT}$ hiPSC-CMs (438 ± 24 and 320 ± 13 ms, respectively) compared with KCNH2$^{WT/WT}$ hiPSC-CMs (196 ± 9 ms). Importantly, the cFPD of the KCNH2$^{PR/WT}$ hiPSC-CMs was significantly prolonged compared with the KCNH2$^{TL/WT}$ hiPSC-CMs. The second KCNH2$^{PR/WT}$ and KCNH2$^{TL/WT}$ clones corroborated these findings, indicating that the differences observed were not clone specific (Figure S3D).

Finally, we evaluated the KCNH2 variant lines using a high-speed optical system that can simultaneously measure the APs, intracellular Ca^{2+} transients and contraction-relaxation kinetics of hiPSC-CM monolayers under paced conditions (van Meer et al., 2019). This enables the rapid assessment of how LQT2-causing mutations affect the complete excitation-contraction coupling cascade. Representative transients of the three measured parameters for each of the lines are shown in Figure 3G. Analysis of the voltage traces also showed a significant increase in APD_{90} for both the KCNH2$^{PR/WT}$ and KCNH2$^{TL/WT}$ hiPSC-CMs (403 ± 35 and 350 ± 21ms, respectively) compared with the KCNH2$^{WT/WT}$ hiPSC-CMs (297 ± 33.5 ms; Figure 3H). Both Ca^{2+} transient and contraction at 90% duration (CaD_{90} and $ContD_{90}$, respectively) were also significantly prolonged in the KCNH2 variant lines compared with the wild-type hiPSC-CMs (KCNH2$^{WT/WT}$: 570 ± 8 ms [CaD_{90}], 429 ± 18 ms [$ContD_{90}$]; KCNH2$^{PR/WT}$: 620 ± 10 ms [CaD_{90}], 492 ± 23 ms [$ContD_{90}$]; KCNH2$^{TL/WT}$: 610 ± 9 ms [CaD_{90}], 456 ± 6 ms [$ContD_{90}$]).

Also here, the APD_{90} as well as the $ContD_{90}$ of the KCNH2$^{PR/WT}$ hiPSC-CMs were significantly prolonged compared with the KCNH2$^{TL/WT}$ hiPSC-CMs. Although the CaD_{90} appeared prolonged, this did not reach significance (p = 0.05). These results indicate that when the two variants are examined as confluent monolayers, differences in I_{Kr} density are also reflected in the electrophysiological phenotype of the hiPSC-CMs, with the dominant-

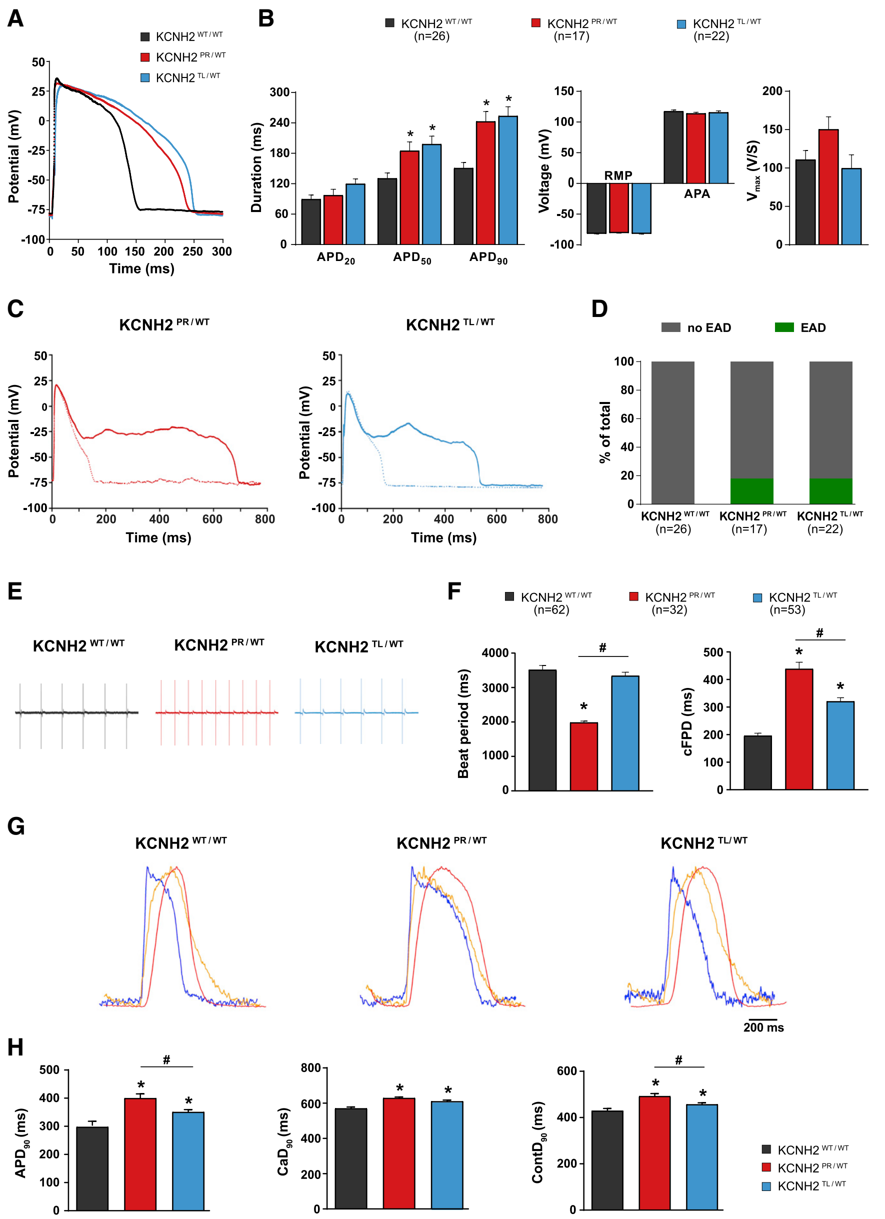

Figure 3. Electrophysiological Characterization of the KCNH2$^{WT/WT}$, KCNH2$^{PR/WT}$, and KCNH2$^{TL/WT}$ hiPSC-CMs

(A and B) Representative AP traces (A), and average APD_{20}, APD_{50}, APD_{90}, RMP, AP amplitude (APA), and V_{max} values (B) for the indicated lines paced at 1 Hz. *Statistical significance to KCNH2$^{WT/WT}$ (KCNH2$^{PR/WT}$: APD_{50}, $p < 0.05$; APD_{90}, $p < 0.001$; KCNH2$^{TL/WT}$: APD_{50}, $p < 0.01$; APD_{90}, $p = 0.0001$; one-way ANOVA with Tukey's multiple comparisons test). Values (n) refer to the number of individual hiPSC-CMs analyzed.

(C) Consecutive AP traces from a KCNH2$^{PR/WT}$ (left) and KCNH2$^{TL/WT}$ (right) hiPSC-CM with and without (solid and dotted lines, respectively) oscillations in membrane potential interrupting the repolarization and indicating the occurrence of EADs.

(D) Percentage of the indicated lines displaying EADs when paced at 0.2 Hz. Values (n) refer to the number of individual hiPSC-CMs analyzed.

(E and F) Representative MEA traces (E) and average values for beat period and cFPD (F) for the indicated lines. *Statistical significance to KCNH2$^{WT/WT}$ (KCNH2$^{PR/WT}$: beat period, cFPD, $p < 0.0001$; KCNH2$^{TL/WT}$: cFPD, $p < 0.0001$; one-way ANOVA with Tukey's multiple comparisons test. #Significance between KCNH2$^{PR/WT}$ and KCNH2$^{TL/WT}$ (beat period, cFPD, $p < 0.0001$). Values (n) refer to the number of independent wells analyzed from at least four differentiations for each cell line.

G) Representative averaged time plots of baseline-normalized fluorescence signals for the indicated lines stimulated at 1.2 Hz. AP traces are shown in blue, cytosolic Ca^{2+} flux in orange, and contraction-relaxation in red.

(H) Average APD_{90}, CaD_{90}, and $ContD_{90}$ values for the indicated lines as determined by changes in fluorescent signal. *Statistical significance to KCNH2$^{WT/WT}$ (KCNH2$^{PR/WT}$: APD_{90}, CaD_{90}, $ContD_{90}$, $p \leq 0.0001$; KCNH2$^{TL/WT}$: APD_{90}, $ContD_{90}$, $p < 0.05$; CaD_{90}, $p < 0.001$); #statistical significance between KCNH2$^{PR/WT}$ and KCNH2$^{TL/WT}$ (APD_{90}, $ContD_{90}$, $p < 0.05$); n = 35–39 recordings from three differentiations for each cell line (Student's t test following one-way ANOVA with Tukey's multiple comparisons test).

Error bars represent SEM.

See also Figure S3.

negative-causing *KCNH2* mutation leading to a more pronounced increase in cFPD and APD than the haploinsufficiency-causing *KCNH2* mutation. Furthermore, these differences are also reflected in both intracellular Ca^{2+} transients and contraction-relaxation kinetics, suggesting that these parameters are also differentially influenced by mild and severe LQT2-causing mutations.

KCNH2$^{PR/WT}$ and KCNH2$^{TL/WT}$ hiPSC-CMs Exhibit Differing Sensitivities to E-4031

To determine if the electrophysiological differences observed between the three lines also led to differing responses to known arrhythmogenic compounds, we examined the response of the hiPSC-CMs to E-4031 (Figure 4). Figure 4A shows representative FP recordings in the

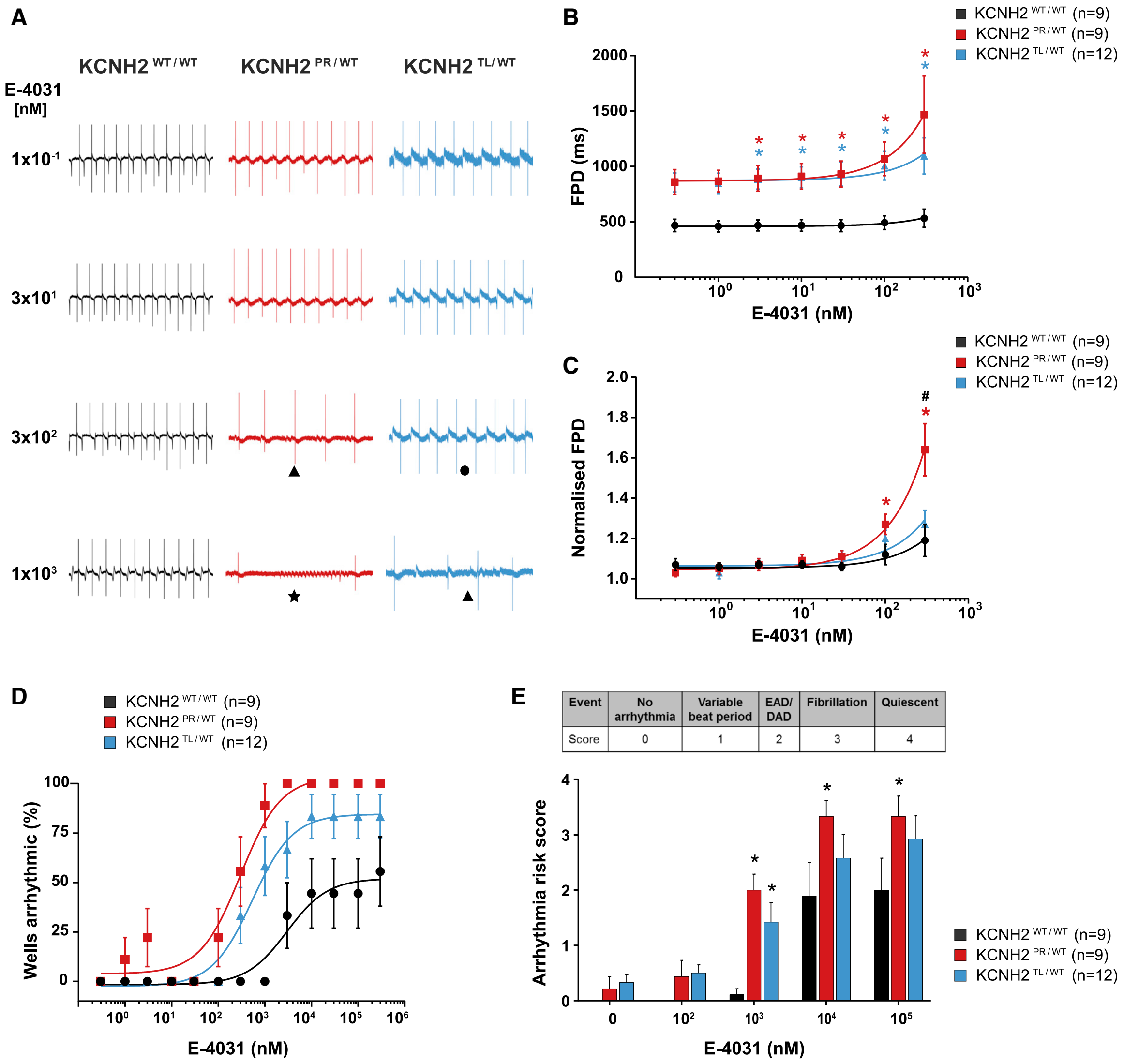

Figure 4. Effect of I_{Kr} Blockade on FPD and Arrhythmogenesis in $KCNH2^{WT/WT}$, $KCNH2^{PR/WT}$, and $KCNH2^{TL/WT}$ hiPSC-CMs

(A) Representative MEA traces highlighting the differences between the indicated lines in the development of arrhythmic events during a recording as E-4031 is cumulatively added. Symbols indicate examples of the different types of arrhythmias detected: (●) variable beat period; (▲) abnormal repolarizations; (★) fibrillation.

(B and C) FPD (B) and FPD normalized to baseline (C) of the indicated lines upon accumulative addition of E-4031. *Statistical significance to $KCNH2^{WT/WT}$ (FPD: 3–300 nM, $p < 0.05$; normalized FPD: 100 nM, $p < 0.05$; 300 nM, $p < 0.0001$); #statistical significance between $KCNH2^{PR/WT}$ and $KCNH2^{TL/WT}$ ($p < 0.0001$); two-way ANOVA with Tukey's multiple comparisons test.

(D) Scatterplot illustrating relationship between occurrence of arrhythmic events and concentration of E-4031 for the indicated lines. Curve fitting with nonlinear regression.

(E) Arrhythmia risk scoring system and bar graph summarizing the arrhythmia risk for each of the cell lines at different concentrations of E-4031. EAD/DAD, abnormal repolarization; *statistical significance to $KCNH2^{WT/WT}$ ($p < 0.05$; two-way ANOVA with Tukey's multiple comparisons test). Values in figure (n) refer to the number of independent wells analyzed from at least three differentiations for each cell line.

Error bars represent SEM.

See also Figure S4.

presence of increasing concentrations of E-4031, with arrhythmic responses, such as EAD-like events or fibrillations, detected in all three lines. As spontaneous beating ceased in some recordings when the cells were exposed to >300 nM E-4031 (Figure S4A), analysis of the effect of E-4031 on FPD prolongation was performed up to this

concentration. The FPD of KCNH2$^{PR/WT}$ and KCNH2$^{TL/WT}$ hiPSC-CMs was significantly prolonged compared with KCNH2$^{WT/WT}$ hiPSC-CMs at >1 nM E-4031 (Figure 4B). When FPD was normalized to baseline measurements, the change in FPD at 300 nM was significantly different for the KCNH2$^{PR/WT}$ hiPSC-CMs compared with the other two lines (Figure 4C), indicating that the KCNH2$^{PR/WT}$ hiPSC-CMs were more sensitive to I_{Kr} blockade. Although the beat period between cell lines varied, it was unaffected for both the KCNH2$^{TL/WT}$ and KCNH2$^{WT/WT}$ hiPSC-CMs at <10 μM E-4031 (Figure S4B). The cFPD was also examined, with differences between the KCNH2$^{PR/WT}$ hiPSC-CMs and the other two lines still discernible (Figures S4C and S4D).

The second KCNH2$^{PR/WT}$ and KCNH2$^{TL/WT}$ clones also showed similar differences in sensitivity to E-4031 following FPD normalization, with analysis performed to 100 nM E-4031 due to some KCNH2$^{PR/WT}$ hiPSC-CMs becoming quiescent at 300 nM (Figures S4A and S4E). Analysis of cFPD showed a similar trend, although it was not significant (Figure S4E), possibly due to overcompensation of the FPD beat rate dependence for the KCNH2$^{TL/WT}$ cl2 hiPSC-CMs (Figures S4F and S4G). Overall, the multiple analyses we have performed with two separate clones for each mutation clearly demonstrate that the KCNH2-A561T mutation results in hiPSC-CMs that are more sensitive to I_{Kr} blockade than the KCNH2-N996I mutation.

To determine if these E-4031-induced differences in FPD between the lines also led to changes in the frequency of arrhythmia-like events, we examined the FP recordings for the occurrence of abnormal repolarizations, fibrillation, and quiescence. Persistent E-4031-induced arrhythmic events were first detected in the KCNH2$^{PR/WT}$, KCNH2$^{TL/WT}$, and KCNH2$^{WT/WT}$ hiPSC-CMs at 100 nM, 300 nM, and 1 μM, respectively (Figure S4A). We also quantified the proportion of recordings that exhibited these arrhythmic responses with increasing concentrations of E-4031 (Figures 4A and 4D). Here too the KCNH2$^{PR/WT}$ hiPSC-CMs were the most predisposed with 100% of recordings showing such events at ≥ 3 μM E-4031, followed by the KCNH2$^{TL/WT}$ hiPSC-CMs with >80% of recordings becoming arrhythmic; while <55% of KCNH2$^{WT/WT}$ hiPSC-CMs recordings showed such a response even at the highest E-4031 concentration (300 μM). The E-4031 concentration that resulted in 50% of the maximal response was also significantly different between the lines (KCNH2$^{PR/WT}$: 298 nM; KCNH2$^{TL/WT}$: 536 nM; KCNH2$^{WT/WT}$: 2.98 μM; $p < 0.01$). This increased susceptibility to E-4031-induced arrhythmia-like events in the KCNH2$^{PR/WT}$ hiPSC-CMs was also observed in the second set of KCNH2$^{PR/WT}$ and KCNH2$^{TL/WT}$ clones examined (Figure S4H).

Finally, we investigated the possibility of developing a scoring system based on methods described previously (Blinova et al., 2018; Shaheen et al., 2018) to estimate the arrhythmogenic risk to E-4031 for the different hiPSC-CM lines. We included variable beat period (score 1), which has previously been classified as a “mild” arrhythmia type (Blinova et al., 2018) as an additional category. Abnormal repolarization was given a score of 2, while hiPSC-CMs that were fibrillating or became quiescent were scored 3 and 4, respectively. Both KCNH2$^{PR/WT}$ and KCNH2$^{TL/WT}$ hiPSC-CMs had higher arrhythmia risk scores compared with the KCNH2$^{WT/WT}$ hiPSC-CMs at all concentrations of E-4031 analyzed, with both lines significantly greater at 1 μM E-4031 and these differences remaining between the KCNH2$^{PR/WT}$ and KCNH2$^{WT/WT}$ hiPSC-CMs at higher concentrations (Figure 4E). Differences in the arrhythmia risk score were also observed between the second KCNH2$^{PR/WT}$ and KCNH2$^{TL/WT}$ clones (Figure S4I). Taken together, these results demonstrate a difference in susceptibility to arrhythmias between the variant lines and the KCNH2$^{WT/WT}$ hiPSC-CMs, with KCNH2$^{PR/WT}$ hiPSC-CMs more sensitive to E-4031 than the KCNH2$^{TL/WT}$ hiPSC-CMs.

DISCUSSION

Interpreting the functional consequences of potential disease-causing variants in LQTS patients is often inconclusive due to the variable expressivity and incomplete penetrance of these diseases (Giudicessi and Ackerman, 2013), as well as the high level of background genetic variation observed in LQTS-susceptibility genes (Giudicessi et al., 2012). The ability to generate hiPSCs from patients, combined with advances in genome editing technologies, has demonstrated how such a platform can be used to determine the pathogenicity of variants of uncertain significance (Garg et al., 2018; Ma et al., 2018), or the contribution of genetic modifiers to the disease phenotype (Chai et al., 2018). However, the extent to which hiPSCs can reflect intragenotype differences in disease risk such as that observed between LQT2 patients has not been fully explored (van den Brink et al., 2020). Here, we provide evidence that genetically matched hiPSC lines can model differences in disease severity attributable to the *KCNH2* mutation.

Most *KCNH2* mutations in the cytoplasmic tail cause haploinsufficiency, with mutant subunits failing to co-assemble with wild-type subunits (Kaufman, 2013). This results in approximately half of the usual number of functional channels and a $\leq$50% reduction in I_{Kr}. In contrast, *KCNH2* mutations within the pore region typically have dominant-negative effects (Anderson et al., 2014), thereby resulting in even fewer functional channels and a >50% I_{Kr} reduction (Kaufman, 2013; Vandenberg et al., 2012). In the

hiPSC-CMs we detected a <50% I_{Kr} decrease with the KCNH2-N996I mutation and an ~70% reduction for the KCNH2-A561T mutation, indicating haploinsufficiency and dominant-negative effects, respectively, but also suggesting that tetrameric ion channels containing one KCNH2-A561T subunit remain functional. A previous study evaluating KCNH2-A561T in COS-7 cells observed an almost identical reduction in I_{Kr} current density (70%) (Bellocq et al., 2004). While heterologous expression systems are simple to use and have the advantage that I_{Kr} kinetics can be studied without the need to block other ion channels, the effect of these changes on the functionality of the cardiomyocyte cannot be directly ascertained and the channel is expressed at unnaturally high levels. In contrast, hiPSC-CMs offer a more native cell environment, with hERG as well as other proteins involved in the transduction of the current, such as auxiliary channel subunits, expressed closer to physiological levels, and functional implications on the AP directly measurable.

We hypothesized that the differences in I_{Kr} would also be reflected in the electrophysiological phenotype of the hiPSC-CMs. All three platforms used to assess the electrophysiology of the lines demonstrated a clear prolongation of the APD and FPD for both KCNH2 variant lines compared with the KCNH2$^{WT/WT}$ hiPSC-CMs, with recordings made using confluent monolayers of hiPSC-CMs revealing that the KCNH2$^{PR/WT}$ lines had longer FPs and APs compared with the KCNH2$^{TL/WT}$ hiPSC-CMs. The discrepancy between the data measured by patch clamp compared with the MEA or optical recordings is possibly due to differences in the setup and cellular configuration between the experimental approaches. Sparsely seeded hiPSC-CMs, such as those used for patch-clamp recordings, show greater electrophysiological variability compared with measurements performed on confluent monolayers (Du et al., 2015), which could confound the detection of subtle electrophysiological differences. In addition, both the MEA and optical system offer analysis at higher throughput than traditional patch-clamp techniques, enabling larger numbers of cells to be measured and obtain statistical confidence where phenotypic differences are expected to be small.

We also evaluated the cytosolic Ca^{2+} transients and contraction kinetics of all three lines, as these are also key parameters that can be altered in LQTS (Kiviaho et al., 2015; Leren et al., 2015; Sala et al., 2018; Spencer et al., 2014). As expected, both LQT2-causing mutations resulted in a significant prolongation in Ca^{2+} transients and contraction-relaxation duration when compared with KCNH2$^{WT/WT}$ hiPSC-CMs. We also observed significant differences in contractility kinetics between the two KCNH2 variants. Contraction duration differences are known to exist between symptomatic and asymptomatic LQTS patients (Haugaa et al., 2010), and this has been proposed as an additional parameter to measure alongside QT interval for improving risk stratification in LQTS patients (ter Bekke et al., 2015).

In LQT2 patients, sudden arousal is the most frequent trigger of an arrhythmic cardiac event (Wilde et al., 1999), and patients with mutations in the pore-loop region have a significantly increased risk to this and other (e.g., fever, medication, sleep) triggering factors (Kim et al., 2010). To mimic the effect of such triggers, we examined the behavior of the hiPSC-CMs when treated with the I_{Kr} blocker, E-4031. We observed differing responses to the QT-prolonging drug between the three lines, with the KCNH2$^{PR/WT}$ hiPSC-CMs exhibiting a greater prolongation in normalized FPD than the KCNH2$^{TL/WT}$ or KCNH2$^{WT/WT}$ hiPSC-CMs. These differences in sensitivity to I_{Kr} block could be due to the dominant-negative effect of the KCNH2-PR mutation, additional gating kinetic defects in trafficked hERG channels that include the KCNH2-A561T subunit (Perry et al., 2016), or a combination of both. The results differ from those recently reported by Yoshinaga et al. (2019), who observed a smaller change in FPD in response to I_{Kr} blockade in hiPSC-CMs derived from LQT2 patients than in control or mutation-corrected hiPSC-CMs. This discordance could be mutation-specific as they also observed an increased arrhythmia susceptibility with the LQT2 hiPSC-CMs.

In line with their differing FPD response to I_{Kr} blockade, the KCNH2$^{PR/WT}$ hiPSC-CMs also exhibited an increased occurrence of arrhythmia events in the presence of E-4031. In addition, a greater proportion of measurements from KCNH2$^{TL/WT}$ hiPSC-CMs displayed arrhythmic activity compared with KCNH2$^{WT/WT}$ hiPSC-CMs. Akin to systems being established to evaluate the arrhythmogenic risk of pharmacological compounds (Blinova et al., 2018), a similar matrix could be developed to assess the risk of specific mutations in patients to different triggering conditions. As proof-of-concept, we determined the arrhythmia risk score for all three lines at various concentrations of E-4031, observing that the KCNH2$^{PR/WT}$ hiPSC-CMs had a higher arrhythmogenic risk at 1 μM. It will be necessary to further evaluate this scoring system with a larger panel of *KCNH2* mutations as well as for different triggers, but this study suggests that genetically matched sets of hiPSC-CMs are sufficiently sensitive to detect these subtle intragenotype-phenotype mutational differences. Such an approach could have clinical implications, for example, by identifying particular *KCNH2* mutations that predispose patients to increased arrhythmic risk and whom might benefit from more vigilant monitoring.

Moreover, other methods such as *in silico* prediction tools still perform poorly in even correctly classifying benign

and pathogenic mutations (Musunuru et al., 2018). This study highlights the benefit of introducing mutations into a well-characterized control hiPSC line. Indeed, while technically challenging, this approach is one of the only options to identify subtle mutation-specific functional changes, as genetic background differences would likely mask these if studied in patient-derived hiPSCs. This strategy also means that the mutations examined are not limited by the availability of patient material. In addition, this method is cheaper and faster as the isogenic control for each variant does not need to be generated and characterized. While common variants present in the control hiPSC line might modify the disease phenotype, in our approach all mutations are evaluated on the same genetic background, thereby nullifying their effect. Furthermore, as similar responses were detected in both clonal lines generated for each mutation, the differences observed between the experimental groups are unlikely to be due to variants that have arisen spontaneously in culture or from CRISPR-Cas9-induced off-target effects.

In conclusion, we have established that genetically matched hiPSC-CMs can capture electrophysiological differences related to the *KCNH2* mutation, with these differences also reflected in the occurrence of drug-induced arrhythmias. This study demonstrates an application in which hiPSC-CMs could be used to model mutation-location risk differences seen in LQT2 patients and contribute to improvements in the diagnosis, prognosis, and risk stratification of patients with congenital LQTS.

EXPERIMENTAL PROCEDURES

An extended methods section is provided in the Supplemental Information.

Genome Editing

The *KCNH2* variants (c.G1681A and c.A2987T) were introduced into a hiPSC control ($KCNH2^{WT/WT}$; LUMC0020iCTRL-06) (Zhang et al., 2014) line by CRISPR-Cas9-mediated gene editing. Heterozygosity of clones was confirmed by Sanger sequencing. Sequences of the guide RNAs, single-strand oligonucleotides (ssODNs), and PCR primers used in this study are listed in Tables S3 and S4.

Differentiation to hiPSC-CM

The hiPSC lines were differentiated into cardiomyocytes as described in the Supplemental Information. All analyses were performed on cryopreserved hiPSC-CMs 5–9 days after thawing.

Electrophysiology

Voltage-clamp recordings of I_{Kr} were made using pipette and bath solutions as described previously (Bellin et al., 2013). APs of the hiPSC-CMs were recorded by perforated patch clamp and the dynamic clamp technique with injection of an inward rectifier potassium current (I_{K1}) used to achieve a close-to-physiological RMP (Verkerk et al., 2017). For electrophysiological analysis on MEAs, the FP was recorded as described previously (Sala et al., 2017). Sequential addition of increasing concentrations of E-4031, a specific I_{Kr} blocker, was performed with recordings initiated following 1 min of incubation.

Optical Recordings

The hiPSC-CMs were labeled with organic fluorescent dyes and the resulting signals recorded and analyzed as described in the Supplemental Information.

Statistical Analysis

Results are presented as mean ± SEM, with comparison between groups performed using one-way or two-way ANOVA followed by Tukey's multiple comparisons test for *post hoc* analysis. Pairwise comparisons were also performed using the Student's t test following one-way ANOVA if one of the null hypotheses could be rejected (Goeman and Solari, 2011). Curve fitting from regression models and statistical analyses was performed using GraphPad Prism 8 software, with $p < 0.05$ considered statistically significant.

Data and Code Availability

Requests for the data used in this paper, including the genome sequencing data, should be directed to and will be fulfilled by the corresponding author.

SUPPLEMENTAL INFORMATION

Supplemental Information can be found online at https://doi.org/10.1016/j.stemcr.2020.10.005.

AUTHOR CONTRIBUTIONS

Conceptualization, K.O.B. and R.P.D.; Methodology, K.O.B., L.v.d.B., D.C.M., C.G., B.J.v.M., M.P.H.M., T.d.K., and R.P.D.; Investigation, K.O.B., L.v.d.B., D.C.M., T.d.K., A.O.V., and R.P.D.; Supervision, C.L.M. and R.P.D.; Formal Analysis, K.O.B., L.v.d.B., D.C.M., T.d.K., L.S., and R.P.D.; Software, B.J.v.M. and L.G.J.T.; Writing – Original Draft, K.O.B. and R.P.D.; Writing – Review & Editing, K.O.B., L.v.d.B., D.C.M., C.G., B.J.v.M., C.L.M., L.S., and R.P.D.; Funding Acquisition, R.P.D.

CONFLICTS OF INTERESTS

C.L.M. is a cofounder of Pluriomics B.V. (now Ncardia B.V.).

ACKNOWLEDGMENTS

We thank M. Gomes Fernandes for assistance with immunofluorescence images, J. Goeman for statistical advice, A. Krotenberg for technical assistance in performing the optical recordings, Y. Ge for help with western blots, and M. Bellin for providing the control (LUMC0020iCTRL) hiPSC line and constructive comments on the manuscript. We also acknowledge Niels Geijsen for providing the Cas9 protein. The graphical abstract was created with BioRender.com. Funding: This work was supported by a Starting Grant (STEMCARDIORISK) from the European Research Council (ERC) under the European Union's Horizon 2020 Research And

Innovation Programme (H2020 European Research Council; grant agreement no. 638030), and a VIDI fellowship from the Netherlands Organisation for Scientific Research (Nederlandse Organisatie voor Wetenschappelijk Onderzoek NWO; ILLUMINATE; no. 91715303), and the Marie Skłodowska-Curie Individual Fellowship (H2020-MSCA-IF-2017; no. 795209 to L.S.).

Received: December 12, 2019
Revised: October 12, 2020
Accepted: October 13, 2020
Published: November 10, 2020

REFERENCES

Anderson, C.L., Kuzmicki, C.E., Childs, R.R., Hintz, C.J., Delisle, B.P., and January, C.T. (2014). Large-scale mutational analysis of Kv11.1 reveals molecular insights into type 2 long QT syndrome. Nat. Commun. *5*, 5535.

ter Bekke, R.M.A., Haugaa, K.H., van den Wijngaard, A., Bos, J.M., Ackerman, M.J., Edvardsen, T., and Volders, P.G.A. (2015). Electromechanical window negativity in genotyped long-QT syndrome patients: relation to arrhythmia risk. Eur. Heart J. *36*, 179–186.

Bellin, M., Casini, S., Davis, R.P., D'Aniello, C., Haas, J., Ward-van Oostwaard, D., Tertoolen, L.G.J., Jung, C.B., Elliott, D.A., Welling, A., et al. (2013). Isogenic human pluripotent stem cell pairs reveal the role of a KCNH2 mutation in long-QT syndrome. EMBO J. *32*, 3161–3175.

Bellocq, C., Wilders, R., Schott, J.-J., Louérat-Oriou, B., Boisseau, P., Le Marec, H., Escande, D., and Baró, I. (2004). A common antitussive drug, clobutinol, precipitates the long QT syndrome 2. Mol. Pharmacol. *66*, 1093–1102.

Bezzina, C.R., Lahrouchi, N., and Priori, S.G. (2015). Genetics of sudden cardiac death. Circ. Res. *116*, 1919–1936.

Blinova, K., Dang, Q., Millard, D., Smith, G., Pierson, J., Guo, L., Brock, M., Lu, H.R., Kraushaar, U., Zeng, H., et al. (2018). International multisite study of human-induced pluripotent stem cell-derived cardiomyocytes for drug proarrhythmic potential assessment. Cell Rep. *24*, 3582–3592.

Brandão, K.O., Tabel, V.A., Atsma, D.E., Mummery, C.L., and Davis, R.P. (2017). Human pluripotent stem cell models of cardiac disease: from mechanisms to therapies. Dis. Model. Mech. *10*, 1039–1059.

van den Brink, L., Grandela, C., Mummery, C.L., and Davis, R.P. (2020). Concise review: inherited cardiac diseases, pluripotent stem cells, and genome editing combined—the past, present, and future. Stem Cells *38*, 174–186.

Chai, S., Wan, X., Ramirez-Navarro, A., Tesar, P.J., Kaufman, E.S., Ficker, E., George, A.L., and Deschênes, I. (2018). Physiological genomics identifies genetic modifiers of long QT syndrome type 2 severity. J. Clin. Invest. *128*, 1043–1056.

Curran, M.E., Splawski, I., Timothy, K.W., Vincen, G.M., Green, E.D., and Keating, M.T. (1995). A molecular basis for cardiac arrhythmia: HERG mutations cause long QT syndrome. Cell *80*, 795–803.

Devalla, H.D., Schwach, V., Ford, J.W., Milnes, J.T., El-Haou, S., Jackson, C., Gkatzis, K., Elliott, D.A., Chuva de Sousa Lopes, S.M., Mummery, C.L., et al. (2015). Atrial-like cardiomyocytes from human pluripotent stem cells are a robust preclinical model for assessing atrial-selective pharmacology. EMBO Mol. Med. *7*, 394–410.

Du, D.T.M., Hellen, N., Kane, C., and Terracciano, C.M.N. (2015). Action potential morphology of human induced pluripotent stem cell-derived cardiomyocytes does not predict cardiac chamber specificity and is dependent on cell density. Biophys. J. *108*, 1–4.

Garg, P., Oikonomopoulos, A., Chen, H., Li, Y., Lam, C.K., Sallam, K., Perez, M., Lux, R.L., Sanguinetti, M.C., and Wu, J.C. (2018). Genome editing of induced pluripotent stem cells to decipher cardiac channelopathy variant. J. Am. Coll. Cardiol. *72*, 62–75.

George, A.L. (2013). Molecular and genetic basis of sudden cardiac death. J. Clin. Invest. *123*, 75–83.

Giudicessi, J.R., and Ackerman, M.J. (2013). Determinants of incomplete penetrance and variable expressivity in heritable cardiac arrhythmia syndromes. Transl. Res. *161*, 1–14.

Giudicessi, J.R., Kapplinger, J.D., Tester, D.J., Alders, M., Salisbury, B.A., Wilde, A.A.M., and Ackerman, M.J. (2012). Phylogenetic and physicochemical analyses enhance the classification of rare nonsynonymous single nucleotide variants in type 1 and 2 long-QT syndrome. Circ. Cardiovasc. Genet. *5*, 519–528.

Goeman, J.J., and Solari, A. (2011). Multiple testing for exploratory Research. Stat. Sci. *26*, 584–597.

Haugaa, K.H., Amlie, J.P., Berge, K.E., Leren, T.P., Smiseth, O.A., and Edvardsen, T. (2010). Transmural differences in myocardial contraction in long-QT syndrome. Circulation *122*, 1355–1363.

Itzhaki, I., Maizels, L., Huber, I., Zwi-Dantsis, L., Caspi, O., Winterstern, A., Feldman, O., Gepstein, A., Arbel, G., Hammerman, H., et al. (2011). Modelling the long QT syndrome with induced pluripotent stem cells. Nature *471*, 225–229.

Kaufman, E.S. (2013). Mutation location matters in long QT syndrome type 2 (but behavior matters more). Heart Rhythm *10*, 68–69.

Kim, J.A., Lopes, C.M., Moss, A.J., McNitt, S., Barsheshet, A., Robinson, J.L., Zareba, W., Ackerman, M.J., Kaufman, E.S., Towbin, J.A., et al. (2010). Trigger-specific risk factors and response to therapy in long QT syndrome type 2. Heart Rhythm. *7*, 1797–1805.

Kiviaho, A.L., Ahola, A., Larsson, K., Penttinen, K., Swan, H., Pekkanen-Mattila, M., Venäläinen, H., Paavola, K., Hyttinen, J., and Aalto-Setälä, K. (2015). Distinct electrophysiological and mechanical beating phenotypes of long QT syndrome type 1-specific cardiomyocytes carrying different mutations. Int. J. Cardiol. Heart Vasc. *8*, 19–31.

Lefeber, D.J., de Brouwer, A.P.M., Morava, E., Riemersma, M., Schuurs-Hoeijmakers, J.H.M., Absmanner, B., Verrijp, K., van den Akker, W.M.R., Huijben, K., Steenbergen, G., et al. (2011). Autosomal recessive dilated cardiomyopathy due to DOLK mutations results from abnormal dystroglycan O-mannosylation. PLoS Genet. *7*, e1002427.

Leren, I.S., Hasselberg, N.E., Saberniak, J., Håland, T.F., Kongsgård, E., Smiseth, O.A., Edvardsen, T., and Haugaa, K.H. (2015). Cardiac mechanical alterations and genotype specific differences in

subjects with long QT syndrome. JACC Cardiovasc. Imaging *8*, 501–510.

Ma, N., Zhang, J.Z., Itzhaki, I., Zhang, S.L., Chen, H., Haddad, F., Kitani, T., Wilson, K.D., Tian, L., Shrestha, R., et al. (2018). Determining the pathogenicity of a genomic variant of uncertain significance using CRISPR/Cas9 and human-induced pluripotent stem cells. Circulation *138*, 2666–2681.

Matsa, E., Rajamohan, D., Dick, E., Young, L., Mellor, I., Staniforth, A., and Denning, C. (2011). Drug evaluation in cardiomyocytes derived from human induced pluripotent stem cells carrying a long QT syndrome type 2 mutation. Eur. Heart J. *32*, 952–962.

Matsa, E., Dixon, J.E., Medway, C., Georgiou, O., Patel, M.J., Morgan, K., Kemp, P.J., Staniforth, A., Mellor, I., and Denning, C. (2014). Allele-specific RNA interference rescues the long-QT syndrome phenotype in human-induced pluripotency stem cell cardiomyocytes. Eur. Heart J. *35*, 1078–1087.

van Meer, B.J., Krotenberg, A., Sala, L., Davis, R.P., Eschenhagen, T., Denning, C., Tertoolen, L.G.J., and Mummery, C.L. (2019). Simultaneous measurement of excitation-contraction coupling parameters identifies mechanisms underlying contractile responses of hiPSC-derived cardiomyocytes. Nat. Commun. *10*, 4325.

Mehta, A., Ramachandra, C.J.A., Singh, P., Chitre, A., Lua, C.H., Mura, M., Crotti, L., Wong, P., Schwartz, P.J., Gnecchi, M., et al. (2018). Identification of a targeted and testable antiarrhythmic therapy for long-QT syndrome type 2 using a patient-specific cellular model. Eur. Heart J. *39*, 1446–1455.

Migdalovich, D., Moss, A.J., Lopes, C.M., Costa, J., Ouellet, G., Barsheshet, A., McNitt, S., Polonsky, S., Robinson, J.L., Zareba, W., et al. (2011). Mutation and gender-specific risk in type 2 long QT syndrome: implications for risk stratification for life-threatening cardiac events in patients with long QT syndrome. Heart Rhythm *8*, 1537–1543.

Moss, A.J., Zareba, W., Kaufman, E.S., Gartman, E., Peterson, D.R., Benhorin, J., Towbin, J.A., Keating, M.T., Priori, S.G., Schwartz, P.J., et al. (2002). Increased risk of arrhythmic events in long-QT syndrome with mutations in the pore region of the human ether-a-go-go-related gene potassium channel. Circulation *105*, 794–799.

Musunuru, K., Sheikh, F., Gupta, R.M., Houser, S.R., Maher, K.O., Milan, D.J., Terzic, A., and Wu, J.C. (2018). Induced pluripotent stem cells for cardiovascular disease modeling and precision medicine: a scientific statement from the American Heart Association. Circ. Genomic Precis. Med. *11*, e000043.

Nagaoka, I., Shimizu, W., Itoh, H., Yamamoto, S., Sakaguchi, T., Oka, Y., Tsuji, K., Ashihara, T., Ito, M., Yoshida, H., et al. (2008). Mutation site dependent variability of cardiac events in Japanese LQT2 form of congenital long-QT syndrome. Circ. J. *72*, 694–699.

Napolitano, C., Mazzanti, A., and Priori, S.G. (2018). Genetic risk stratification in cardiac arrhythmias. Curr. Opin. Cardiol. *33*, 298–303.

Perry, M.D., Ng, C.A., Phan, K., David, E., Steer, K., Hunter, M.J., Mann, S.A., Imtiaz, M., Hill, A.P., Ke, Y., et al. (2016). Rescue of protein expression defects may not be enough to abolish the pro-arrhythmic phenotype of long QT type 2 mutations. J. Physiol. *594*, 4031–4049.

Peterson, L.B., Eskew, J.D., Vielhauer, G.A., and Blagg, B.S.J. (2012). The hERG channel is dependent upon the Hsp90α isoform for maturation and trafficking. Mol. Pharm. *9*, 1841–1846.

Pua, C.J., Bhalshankar, J., Miao, K., Walsh, R., John, S., Lim, S.Q., Chow, K., Buchan, R., Soh, B.Y., Lio, P.M., et al. (2016). Development of a comprehensive sequencing assay for inherited cardiac condition genes. J. Cardiovasc. Transl. Res. *9*, 3–11.

Sala, L., Ward-van Oostwaard, D., Tertoolen, L.G.J., Mummery, C.L., and Bellin, M. (2017). Electrophysiological analysis of human pluripotent stem cell-derived cardiomyocytes (hPSC-CMs) using multi-electrode arrays (MEAs). J. Vis. Exp. *123*, e55587.

Sala, L., van Meer, B.J., Tertoolen, L.G.J., Bakkers, J., Bellin, M., Davis, R.P., Denning, C., Dieben, M.A.E., Eschenhagen, T., Giacomelli, E., et al. (2018). MUSCLEMOTION—a versatile open software tool to quantify cardiomyocyte and cardiac muscle contraction in vitro and in vivo. Circ. Res. *122*, e5–e16.

Schwartz, P.J., Crotti, L., and George, A.L. (2018). Modifier genes for sudden cardiac death. Eur. Heart J. *39*, 3925–3931.

Shaheen, N., Shiti, A., Huber, I., Shinnawi, R., Arbel, G., Gepstein, A., Setter, N., Goldfracht, I., Gruber, A., Chorna, S.V., et al. (2018). Human induced pluripotent stem cell-derived cardiac cell sheets expressing genetically encoded voltage indicator for pharmacological and arrhythmia studies. Stem Cell Reports *10*, 1879–1894.

Shimizu, W., Moss, A.J., Wilde, A.A.M., Towbin, J.A., Ackerman, M.J., January, C.T., Tester, D.J., Zareba, W., Robinson, J.L., Qi, M., et al. (2009). Genotype-phenotype aspects of type 2 long QT syndrome. J. Am. Coll. Cardiol. *54*, 2052–2062.

Spencer, C.I., Baba, S., Nakamura, K., Hua, E.A., Sears, M.A.F., Fu, C., Zhang, J., Balijepalli, S., Tomoda, K., Hayashi, Y., et al. (2014). Calcium transients closely reflect prolonged action potentials in iPSC models of inherited cardiac arrhythmia. Stem Cell Reports *3*, 269–281.

Thomas, D. (2003). Defective protein trafficking in hERG-associated hereditary long QT syndrome (LQT2): molecular mechanisms and restoration of intracellular protein processing. Cardiovasc. Res. *60*, 235–241.

Vandenberg, J.I., Perry, M.D., Perrin, M.J., Mann, S.A., Ke, Y., and Hill, A.P. (2012). hERG K+ channels: structure, function, and clinical significance. Physiol. Rev. *92*, 1393–1478.

Veerman, C.C., Mengarelli, I., Guan, K., Stauske, M., Barc, J., Tan, H.L., Wilde, A.A.M., Verkerk, A.O., and Bezzina, C.R. (2016). hiPSC-derived cardiomyocytes from Brugada syndrome patients without identified mutations do not exhibit clear cellular electrophysiological abnormalities. Sci. Rep. *6*, 30967.

Verkerk, A., Veerman, C., Zegers, J., Mengarelli, I., Bezzina, C., and Wilders, R. (2017). Patch-clamp recording from human induced pluripotent stem cell-derived cardiomyocytes: improving action potential characteristics through dynamic clamp. Int. J. Mol. Sci. *18*, 1873.

Wilde, A.A., Jongbloed, R.J., Doevendans, P.A., Düren, D.R., Hauer, R.N., van Langen, I.M., van Tintelen, J.P., Smeets, H.J., Meyer, H.,

and Geelen, J.L.M. (1999). Auditory stimuli as a trigger for arrhythmic events differentiate HERG-related (LQTS2) patients from KVLQT1-related patients (LQTS1). J. Am. Coll. Cardiol. *33*, 327–332.

Yoshinaga, D., Baba, S., Makiyama, T., Shibata, H., Hirata, T., Akagi, K., Matsuda, K., Kohjitani, H., Wuriyanghai, Y., Umeda, K., et al. (2019). Phenotype-based high-throughput classification of long QT syndrome subtypes using human induced pluripotent stem cells. Stem Cell Reports *13*, 394–404.

Zhang, M., D'Aniello, C., Verkerk, A.O., Wrobel, E., Frank, S., Ward-van Oostwaard, D., Piccini, I., Freund, C., Rao, J., Seebohm, G., et al. (2014). Recessive cardiac phenotypes in induced pluripotent stem cell models of Jervell and Lange-Nielsen syndrome: disease mechanisms and pharmacological rescue. Proc. Natl. Acad. Sci. USA *111*, E5383–E5392.

Zhao, J.T., Hill, A.P., Varghese, A., Cooper, A.A., Swan, H., Laitinen-Forsblom, P.J., Rees, M.I., Skinner, J.R., Campbell, T.J., and Vandenberg, J.I. (2009). Not all hERG pore domain mutations have a severe phenotype: G584S has an inactivation gating defect with mild phenotype compared to G572S, which has a dominant negative trafficking defect and a severe phenotype. J. Cardiovasc. Electrophysiol. *20*, 923–930.

Zhou, Z., Gong, Q., Ye, B., Fan, Z., Makielski, J.C., Robertson, G.A., and January, C.T. (1998a). Properties of HERG channels stably expressed in HEK 293 cells studied at physiological temperature. Biophys. J. *74*, 230–241.

Zhou, Z., Gong, Q., Epstein, M.L., and January, C.T. (1998b). HERG channel dysfunction in human long QT syndrome. J. Biol. Chem. *273*, 21061–21066.

Stem Cell Reports, Volume *15*

Supplemental Information

Isogenic Sets of hiPSC-CMs Harboring Distinct *KCNH2* Mutations Differ Functionally and in Susceptibility to Drug-Induced Arrhythmias

Karina O. Brandão, Lettine van den Brink, Duncan C. Miller, Catarina Grandela, Berend J. van Meer, Mervyn P.H. Mol, Tessa de Korte, Leon G.J. Tertoolen, Christine L. Mummery, Luca Sala, Arie O. Verkerk, and Richard P. Davis

Supplemental Figures

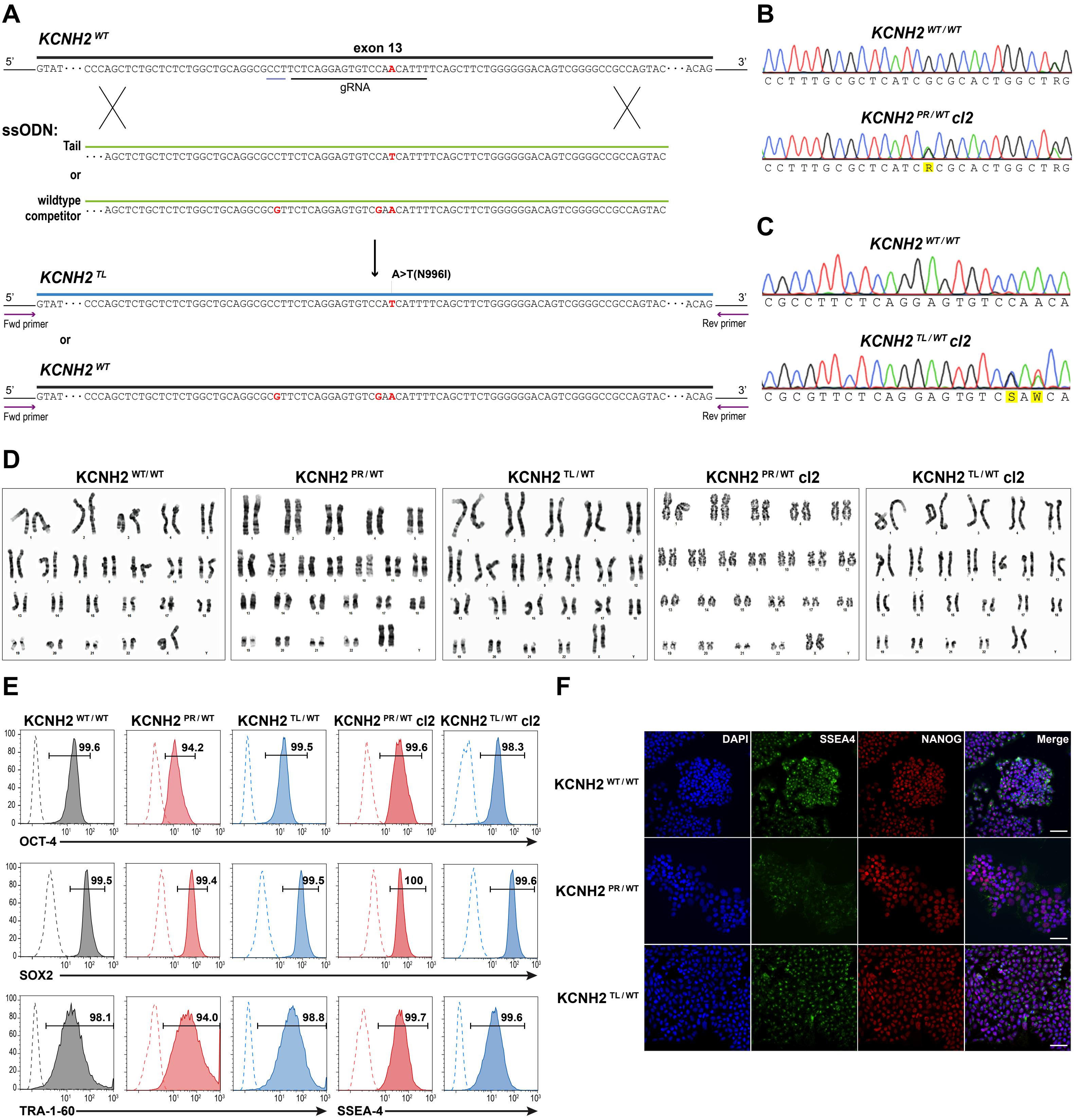

Figure S1: Generation and characterisation of the set of isogenic KCNH2 hiPSC lines. Related to Figure 1. (A) Schematic outlining the alternative gene editing strategy used to generate the KCNH2 $^{TL/WT}$ cl2 hiPSC line. The *ssODN Tail* included the c.A2987T (N996I) mutation (red), while the *ssODN wildtype competitor* included silent nucleotide mutations (red) that did not alter any amino acids but assisted with RFLP screening and prevented Cas9 from cutting the modified sequence due to a mutation in the PAM sequence (grey underline).Sequence analysis of PCR-amplified DNA showing **(B)** heterozygous introduction of the c.G1681A (A561T) mutation (highlighted R) in the KCNH2 $^{PR/WT}$ cl2 hiPSC line, and **(C)** heterozygous introduction of the c.A2987T (N996I) mutation (highlighted W), as well as the RFLP screening silent mutation (highlighted S), in the KCNH2 $^{TL/WT}$ cl2 hiPSC line. **(D)** G-band karyograms indicating a normal euploid karyotype for the KCNH2 hiPSC lines used in the study. **(E)** Flow cytometry and **(F)** immunofluorescence analysis of the pluripotency-associated markers, NANOG, OCT4, SOX2, TRA-1-60 and SSEA4 in the indicated hiPSC lines. Scale bar: 50 μm.

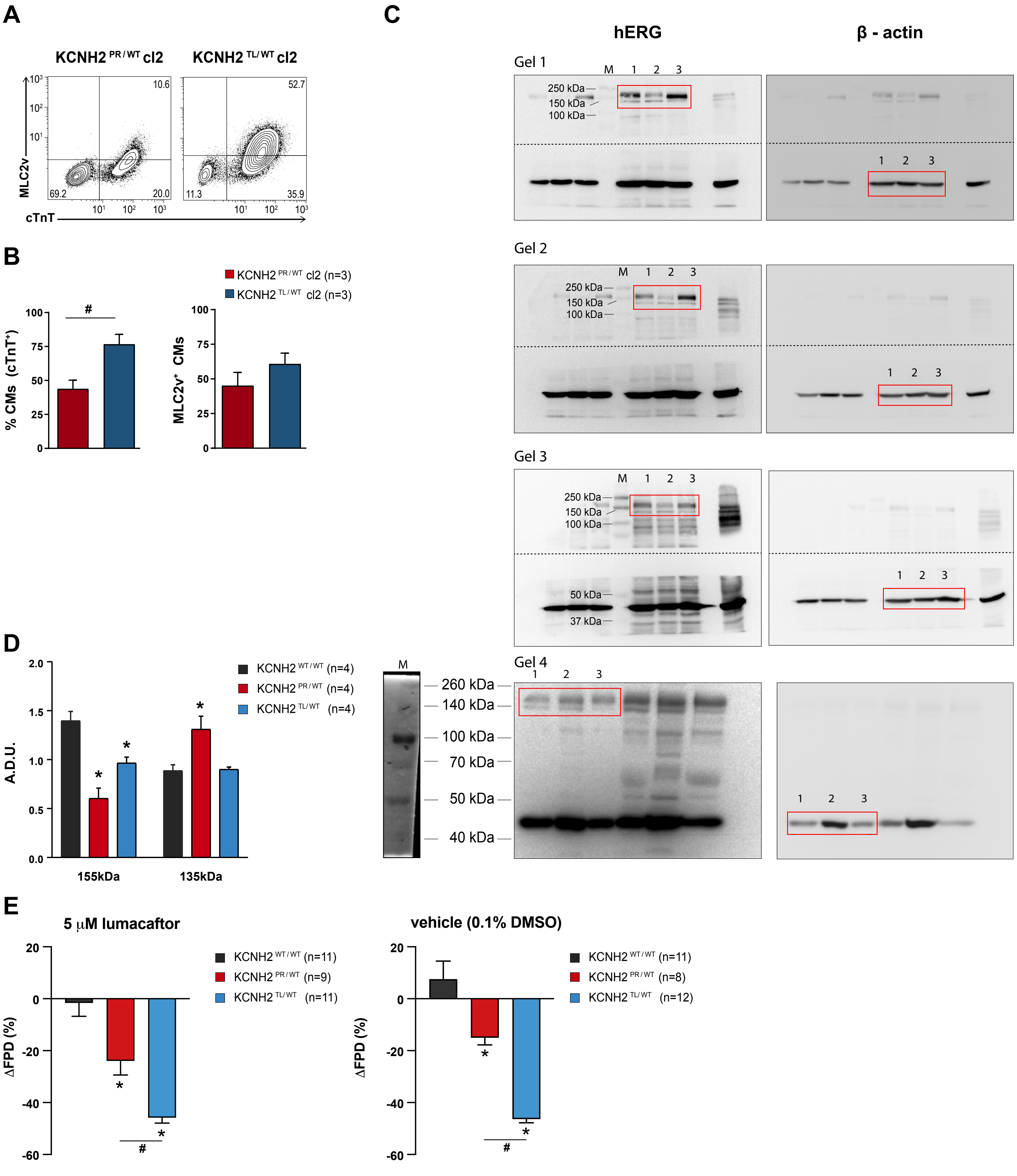

Figure S2: Evaluation of effect of KCNH2 mutations on hERG channel function in hiPSC-CMs. Related to Figure 2. Representative flow cytometry plots **(A)** and overall cardiac differentiation efficiency to ventricular-like cardiomyocytes **(B)** for the KCNH2$^{PR/WT}$ cl2 and KCNH2$^{TL/WT}$ cl2 hiPSC lines (#, *P*=0.02 (two-tailed Student's t-test); *n*, number of independent differentiations). **(C)** Full western blots showing detection of hERG protein bands and β-actin (loading control) in the hiPSC-CMs. Order is: protein marker (M); KCNH2$^{TL/WT}$ (1); KCNH2$^{PR/WT}$ (2); and KCNH2$^{WT/WT}$ (3) hiPSC-CMs. Dotted lines indicate where membrane was cut. The hERG and β-actin bands used for quantification are marked by red boxes. **(D)** Densitometric quantification of the 155 kDa and 135 kDa bands (normalised to β-actin); A.D.U., arbitrary densitometric units (*, *P*<0.01 compared to KCNH2$^{WT/WT}$ (two-way ANOVA with Tukey's multiple comparisons for post hoc test); *n*, number of independent differentiations). **(E)** Percent change in FPD relative to baseline (ΔFPD) after 8 days of treatment with either 5 μM lumacaftor (*left*) or vehicle control (0.1% DMSO; *right*) for the indicated hiPSC-CM lines. A negative value indicates shortening of the FPD. * indicates significance to KCNH2$^{WT/WT}$ (KCNH2$^{PR/WT}$, *P*<0.01; KCNH2$^{PR/WT}$, *P*<0.0001); # indicates statistical significance between KCNH2$^{PR/WT}$ and KCNH2$^{TL/WT}$ (lumacaftor, *P*<0.01; DMSO, *P*<0.001); *n*, number of wells from at least 3 independent differentiations.

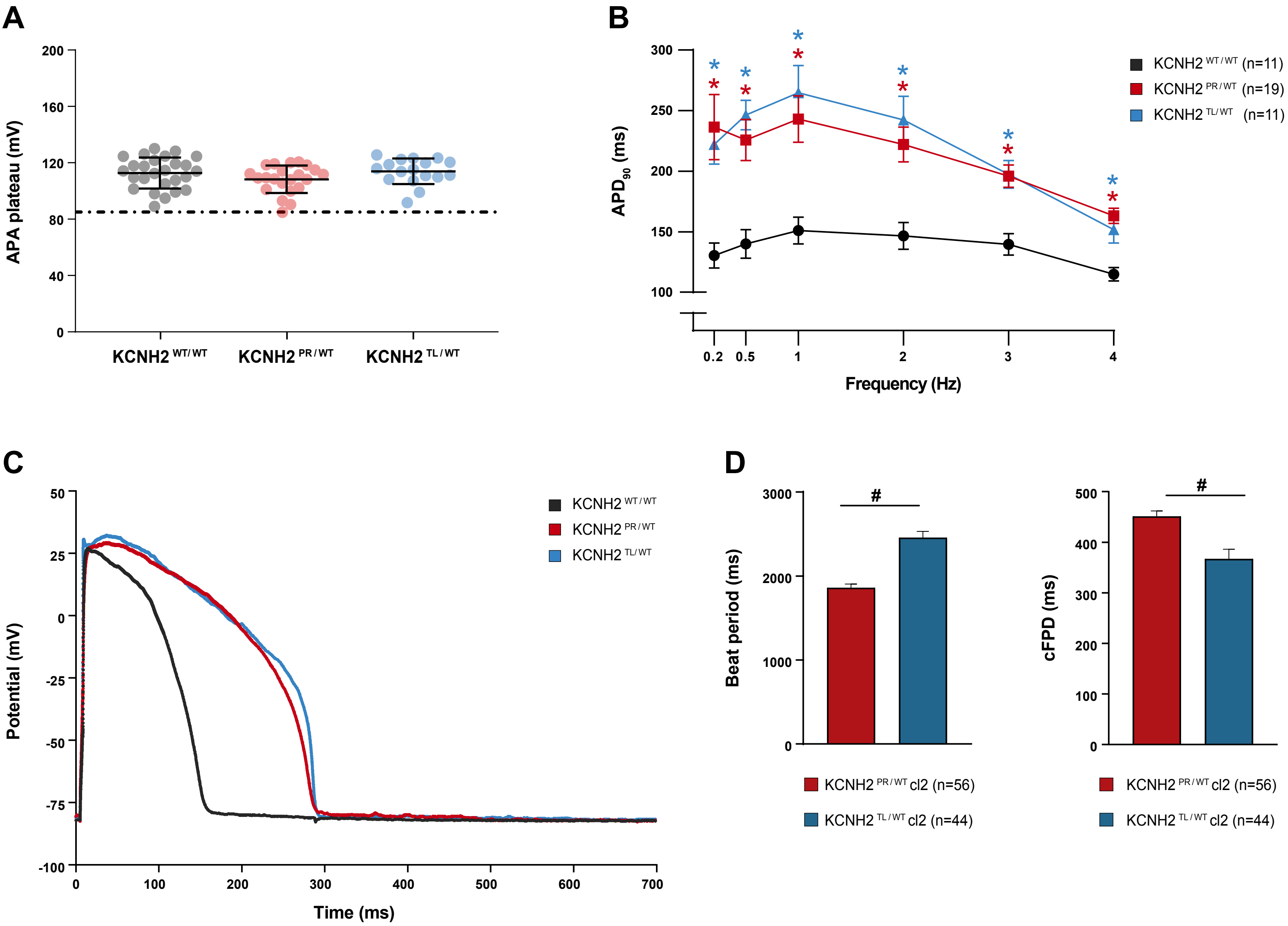

Figure S3: Electrophyisological characterisation of KCNH2 $^{WT/WT}$, KCNH2 $^{PR/WT}$ and KCNH2 $^{TL/WT}$ hiPSC-CMs. Related to Figure 3. (A) Dot plots of AP plateau amplitude measured at 20 ms after initiation of the AP upstroke in the indicated hiPSC-CMs paced at 1 Hz. The dotted line indicates 85 mV. **(B)** Average APD_{90} values for the indicated hiPSC-CMs paced between 0.2–4 Hz. *, P<0.01 compared to KCNH2 $^{WT/WT}$ (two-way ANOVA with Tukey's multiple comparisons); *n*, number of individual hiPSC-CMs analysed. **(C)** Representative examples of non-arrhythmogenic AP traces for indicated hiPSC-CMs paced at 0.2 Hz. **(D)** Average values for beat period interval (*left*) and the cFPD (*right*) for the indicated cell lines. #, P<0.05 (two-tailed Student's t-test); *n,* number of independent wells analysed from at least 3 differentiations.

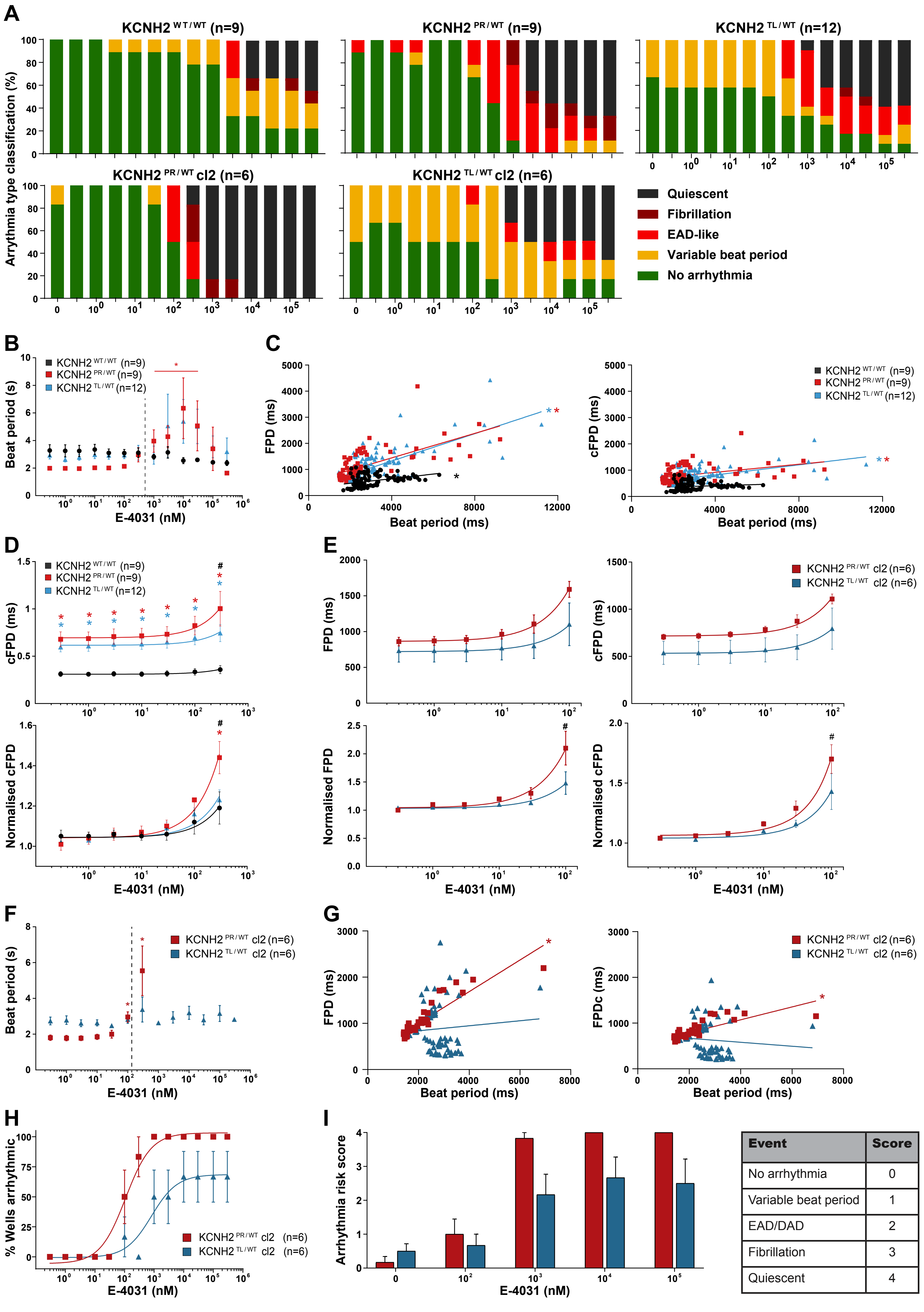
A
KCNH2 WT/WT (n=9)
KCNH2 PR/WT (n=9)
KCNH2 TL/WT (n=12)
KCNH2 PR/WT cl2 (n=6)
KCNH2 TL/WT cl2 (n=6)
Arrythmia type classification (%)
Quiescent
Fibrillation
EAD-like
Variable beat period
No arrhythmia
B
Beat period (s)
E-4031 (nM)
C
FPD (ms)
cFPD (ms)
Beat period (ms)
D
cFPD (ms)
Normalised cFPD
E
FPD (ms)
Normalised FPD
F
G
FPDc (ms)
H
% Wells arrhythmic
I
Arrhythmia risk score
Event
Score
No arrhythmia
0
Variable beat period
1
EAD/DAD
2
Fibrillation
3
Quiescent
4

Figure S4. Analysis of the effect of E-4031 on hiPSC-CMs with different *KCNH2* mutations. Related to Figure 4. (A) Quantification of the arrhythmia subtypes detected with increasing concentrations of E-4031 for the indicated lines. **(B, F)** Analysis of the effect of increasing concentrations of E-4031 (0.3 nM - 300 µM) on the average beat period for the indicated lines. The dotted line demarcates the maximum concentration of E-4031 (300 nM: $KCNH2^{WT/WT}$, $KCNH2^{PR/WT}$ and $KCNH2^{TL/WT}$ hiPSC-CMs; 100 nM: $KCNH2^{PR/WT}$ cl2 and $KCNH2^{TL/WT}$ cl2 hiPSC-CMs) included in the analysis of the effect of this compound on (c)FPD. **(C, G)** Major-axis regression analysis on the relationship between beat period and either FPD (*left graphs*) or cFPD (*right graphs*) for the indicated lines upon increasing concentrations of E-4031. * indicates linear coefficients significantly different from 0 (P<0.0001). **(D)** cFPD (*upper graph*) and cFPD normalised to baseline (*lower graph*) of the indicated lines upon accumulative addition of E-4031. * indicates statistical significance to $KCNH2^{WT/WT}$ (cFPD: 0.3-300 nM, P<0.02; normalised cFPD: 300 nM, P<0.0001); # indicates statistical significance between $KCNH2^{PR/WT}$ and $KCNH2^{TL/WT}$ hiPSC-CMs (P<0.0001). **(E)** FPD **(***upper left graph***)**, FPD normalised to baseline **(***lower left graph***)**, cFPD (*upper right graph***)** and cFPD normalised to baseline **(***lower right graph***)** of $KCNH2^{PR/WT}$ cl2 and $KCNH2^{TL/WT}$ cl2 hiPSC-CMs upon accumulative addition of E-4031. # indicates statistical significance between mutated lines (P<0.006). **(H)** Scatter plot illustrating relationship between occurrence of arrhythmic events and concentration of E-4031 for $KCNH2^{PR/WT}$ cl2 and $KCNH2^{TL/WT}$ cl2 hiPSC-CMs. Curve fitting with nonlinear regression. **(I)** Arrhythmia risk scoring system and bar graph summarising the arrhythmia risk these two cell lines at different concentrations of E- 4031. Values (*n*) refer to the number of independent wells analysed from at least 3 differentiations for each of the cell lines $KCNH2^{WT/WT}$, $KCNH2^{PR/WT}$ and $KCNH2^{TL/WT}$, and from 2 differentiations for the cell lines $KCNH2^{PR/WT}$ cl2 and $KCNH2^{TL/WT}$ cl2.

Supplemental Tables

Table S2: Predicted gRNA off-target exonic sequences analysed for each *KCNH2* variant hiPSC line. Related to Figure 1.

	Off-target Sequence	Mismatch Position	Mismatch	Score	Chromosome	Strand	Gene
**$KCNH2^{PR/WT}$ clones**	CCGCTACTCTGAGTATGGGGCGG	*........*.....*..*.	4	0.02	chr17	+	***KCNH6***
**$KCNH2^{TL/WT}$ clone 1**	CTACCAGAAAATGAAAATGTGGG	.**......**.........	4	0.55	chrX	-	***BEND2***
	TCCCCAGAAGCAAAAAATTTAGG	*..........**.....*.	4	0.49	chr4	-	***HSPA4L***
	AGCCCAGAAACTGAAAATATTGG	**.......*........*.	4	0.46	chr7	-	***ETV1***
	TCCCCAACAGCTGAAAATGTGGG	*.....**............	3	0.42	chr7	-	***SGCE***
	CTGTAAGAAGCTGAAAATGTTGG	.****...............	4	0.2	chr1	+	***PRAMEF20***
**$KCNH2^{TL/WT}$ clone 2**	AATGCAGGACACTTCAGAGAAGG	....**.......*.*....	4	0.58	chr16	-	**ACSM5**
	AAAGTTGGACATTCCAAAGATGG	..*........*...**...	4	0.33	chr2	+	***SCN9A***
	AATGATGGATAATCCTGAAATGG	....*....*.*......*.	4	0.24	chr14	+	***SYT16***
	AAAGTTGGCGATTCCTGAGACGG	..*.....**.*........	4	0.09	chr7	+	***DLX5***
	TAGGATGGACTCTCCTGAGAAGG	*.*.*.....*.........	4	0.07	chr11	+	***CBL***

Table S3: Sequences of gRNAs and ssODNs used to generate the *KCNH2* variant hiPSC lines. Related to Figure 1

$KCNH2^{PR/WT}$ clones

**gRNA#1**	5'-TCGCTACTCAGAGTACGGCG-3'
**ssODN_pore**	5'-GGCTGCTGCGGCTGGTGCGCGTGGCGCGGAAGCTGGATCGCTACTCAGAGTACGGCGCGGCCGTGCTGTTCTTGCTCA TGTGCACCTTTGCGCTCATCGCGCACTGGCTAGCCTGCATCTGGTACG-3'

$KCNH2^{TL/WT}$ clone 1

**gRNA#2**	5'-TGTAAAAGTCGAAGACCCCC-3'
**ssODN_tail**	5'-GACCGGGCGTGGCAGCGGTGGTGCGTCTACCCCGCTCACCCAGCTCTGCTCTCTGGCTGCAGGCGCCTTCTCAGGAGT GTCCATCATTTTCAGCTTCTGGGGGGACAGTCGGGGCCGCCAGTAC-3'

$KCNH2^{TL/WT}$ clone 2

**gRNA#3**	5'-AATGTTGGACACTCCTGAGA-3'
**ssODN_tail**	5'-GACCGGGCGTGGCAGCGGTGGTGCGTCTACCCCGCTCACCCAGCTCTGCTCTCTGGCTGCAGGCGCCTTCTCAGGAGT GTCCATCATTTTCAGCTTCTGGGGGGACAGTCGGGGCCGCCAGTAC-3'
**ssODN_wt competitor**	5'-GACCGGGCGTGGCAGCGGTGGTGCGTCGACCCCGCTCACCCAGCTCTGCTCTCTGGCTGCAGGCGCCTTCTCAGGAGT GTCCAACATTTTCAGCTTCTGGGGGGACAGTCGGGGCCGCCAGTAC-3'

Table S4: List of primers used in this study. Related to Figures 1 and 2.

**Primer name**	**Sequence (5' - 3')**	**Purpose**
**KCNH2_exon 7_Fwd**	CAAGGAGGCAGGTGGTGTAG	amplification of *KCNH2* exon 7
**KCNH2_exon 7_Rev**	CCTCCAACTTGGGTTCCTCC	amplification of *KCNH2* exon 7
**KCNH2_exon 7_seq_Fwd**	CCCCATCAACGGAATGTG	sequencing of *KCNH2* exon 7
**KCNH2_exon 7_seq_Rev**	CACAGCCAATGAGCATGACG	sequencing of *KCNH2* exon 7
**KCNH2_exon 7_cDNA_Fwd**	CTGATCGGGCTGCTGAAGACT	determining *KCNH2* allele transcript expression for KCNH2$^{PR/WT}$
**KCNH2_exon 7_cDNA_Rev**	CCGAAGATGCTAGCGTACATG	determining *KCNH2* allele transcript expression for KCNH2$^{PR/WT}$
**KCNH6_off_target_Fwd**	GCTCTCACTGCTCCTCCATC	amplification of off-target for gRNA#1
**KCNH6_off_target_Rev**	TTCTCGAGTTGGTGTTGGG	amplification of off-target for gRNA#1
**KCNH6_off_target_seq_Fwd**	CTCATCCATGAAGCCTCCCC	sequencing of off-target for gRNA#1
**KCNH6_off_target_seq_Rev**	GCTGAAGGTGAAGTAGAGGG	sequencing of off-target for gRNA#1
**KCNH2_exon 13_Fwd**	CCCTGAGAGCAGTGAGGATG	amplification of *KCNH2* exon 13
**KCNH2_exon 13_Rev**	GGTGGTCACAGCACTGTAGG	amplification of *KCNH2* exon 13
**KCNH2_exon 13_seq_Fwd**	TCAGGTATCCCGGGCGAC	sequencing of *KCNH2* exon 13
**KCNH2_exon 13_seq_Rev**	CTCCCTCTACCAGACAACACC	sequencing of *KCNH2* exon 13
**KCNH2_exon 13_cDNA_Fwd**	CCCTGAGAGCAGTGAGGATG	determining *KCNH2* allele transcript expression for KCNH2$^{TL/WT}$
**KCNH2_exon 13_cDNA_Rev**	GGTGGTCACAGCACTGTAGG	determining *KCNH2* allele transcript expression for KCNH2$^{TL/WT}$
**KCNH2_gRNA#2_IVT_F**	TGTAATACGACTCACTATAGAATGTTGGACACTCCTGAGAGTTTTAGAGCTAGAAATAGC	Forward primer for in vitro transcription of gRNA#2
**KCNH2_gRNA#3_IVT_F**	TGTAATACGACTCACTATAGCCCCCAGAAGCTGAAAATGTGTTTTAGAGCTAGAAATAGC	Forward primer for in vitro transcription of gRNA#3
**gRNA_IVT_Rev**	AGCACCGACTCGGTGCCACT	Reverse primer for in vitro transcription of gRNA#2 and #3
**Off_T.ex13_#2_BEND2_Fwd**	GGGGAAAATTGGGGAAAGGG	assessing potential off-target for gRNA#2
**Off_T.ex13_#2_BEND2_Rev**	CAGCATGTCATGAACTGCAGG	assessing potential off-target for gRNA#2
**Off_T.ex13_#2_HSPA4L_Fwd**	GGTCATGCCCTAAGTCACAGG	assessing potential off-target for gRNA#2
**Off_T.ex13_#2_HSPA4L_Rev**	GACGGGGTTTTGCCATGTTG	assessing potential off-target for gRNA#2
**Off_T.ex13_#2_ETV1_Fwd**	TCCATTTGCGATTTGGTATGGAG	assessing potential off-target for gRNA#2
**Off_T.ex13_#2_ETV1_Rev**	CTGTCTGGCATGTGGGAGTC	assessing potential off-target for gRNA#2
**Off_T.ex13_#2_SGCE_Fwd**	GGTTACCCAGACCGACCTG	assessing potential off-target for gRNA#2
**Off_T.ex13_#2_SGCE_Rev**	GATGTGTTGTTTCCTCCGCC	assessing potential off-target for gRNA#2
**Off_T.ex13_#2_P.20_Fwd**	GTTTGGTCTGAAGCCATGGC	assessing potential off-target for gRNA#2
**Off_T.ex13_#2_P.20_Rev**	CAGGTGGCCTTCGAGGAAAG	assessing potential off-target for gRNA#2
**Off_T.ex13_#3_VWA8_Fwd**	CTTGACTCCCAGCTCTAGCC	assessing potential off-target for gRNA#3
**Off_T.ex13_#3_VWA8_Rev**	CTTTGGGGACTAAGGTGGGG	assessing potential off-target for gRNA#3
**Off_T.ex13_#3_CBL_Fwd**	CCCCTGCTGTGAGACTTCAG	assessing potential off-target for gRNA#3
**Off_T.ex13_#3_CBL_Rev**	AAGGCAGGGGAAAACTGAGG	assessing potential off-target for gRNA#3
**Off_T.ex13_#3_DLX5_Fwd**	GCAAAAACACACACAAGCGC	assessing potential off-target for gRNA#3
**Off_T.ex13_#3_DLX5_Rev**	GCTGTGACCCCCAATCTACC	assessing potential off-target for gRNA#3
**Off_T.ex13_#3_SYT16_Fwd**	CAGTGAGCCTGAAACACAGC	assessing potential off-target for gRNA#3
**Off_T.ex13_#3_SYT16_Rev**	CATGCCCGGCTCTATGCTAG	assessing potential off-target for gRNA#3
**Off_T.ex13_#3_SCN9A_Fwd**	TGTGTCCCCTACCCTGTTCC	assessing potential off-target for gRNA#3
**Off_T.ex13_#3_SCN9A_Rev**	GGTTCAGTACTTTCTTCAGTGGC	assessing potential off-target for gRNA#3
**Off_T.ex13_#3_ACSM5_Fwd**	GTCCATCTGGGGCATCTGAG	assessing potential off-target for gRNA#3
**Off_T.ex13_#3_ACSM5_Rev**	CTCTGCCTCCCGAGTTCAAG	assessing potential off-target for gRNA#3

Supplemental Methods

Culture of human induced pluripotent stem cells (hiPSCs)

For maintenance of the hiPSC lines, Essential 8™ Medium (Gibco) and Vitronectin (VTN-N; Gibco)-coated plates were used. For passaging, the cells were dissociated with TrypLE Select Enzyme (Gibco) and re-plated in Essential 8™ Medium containing RevitaCell™ Supplement (1:200 dilution; Gibco). For gene editing experiments, the hiPSCs were maintained either on irradiated mouse embryonic fibroblasts in human embryonic stem cell medium as previously described (Davis et al., 2009) or in StemFlex™ Medium (Gibco) according to the manufacturer's instructions.

Differentiation and culture of hiPSC-derived cardiomyocytes (hiPSC-CMs)

One day prior to differentiation, hiPSCs were seeded at 3.75 x 10^4/cm^2 into Matrigel® Matrix (BD)-coated wells in Essential 8™ Medium containing RevitaCell™ Supplement. The hiPSCs were differentiated into cardiomyocytes using the Pluricyte Cardiomyocyte Differentiation Kit (NCardia) according to the manufacturer's instructions, with cells maintained in Medium C until day 19-21 of differentiation. The hiPSC-CMs were dissociated with 5x TrypLE Select Enzyme for 10 min at 37°C and cryopreserved. For replating the hiPSC-CMs, the frozen cells were thawed at 37°C and transferred to a conical tube. Immediately thereafter, 1 ml of BPEL medium (van den Berg et al., 2014) was added dropwise (1 drop every 5 s), followed by ~4.7 ml BPEL (1 drop every 2 s). Cells were pelleted at 250*g* for 3 min, resuspended in Medium C and plated as required.

Exome sequencing

Genomic DNA (gDNA) from $KCNH2^{WT/WT}$ hiPSCs was extracted using the Blood and Cultured Cells DNA Mini Kit (Qiagen), according to manufacturer's instructions. Whole exome sequencing was performed by BGI (Hong Kong) using a HiSeq X platform (Illumina), with the library constructed from 6 µg gDNA using the SureSelect Human All Exon V5 +UTR enrichment kit (Agilent). Bioinformatics analysis (Mendel disease) was performed by BGI. Exome sequencing coverage and variant calls were manually curated for 107 genes known to be linked to inherited arrhythmia syndromes or cardiomyopathies (Pua et al., 2016).

Generation of the guide RNAs (gRNAs)

Candidate gRNAs were identified around the intended mutation site using the bioinformatic website http://crispor.tefor.net, and gRNAs with higher specificity were favoured to minimise potential off-target sites (Table S3). The gRNAs were either synthesised as chimeric single gRNA (sgRNA) by *in vitro* transcription or purchased as crRNA and complexed with tracrRNA (both from IDT) prior to transfection.

For *in vitro* transcription, primers containing the T7 promoter, crRNA and a sequence homologous to the tracrRNA component present in the vector pSp-Cas9(BB)-2A-puro_v2 (Addgene #62988) were designed along with a primer complementary to the 3' end of the tracrRNA (Table S4). A 110bp amplicon was generated by PCR and purified using the QiaQuick PCR purification kit (Qiagen). The sgRNAs were transcribed using the HiScribe T7 kit (New England Biolabs). Following purification using the NucleoSpin RNA Clean-up XS kit (Macherey-Nagel), the integrity of the sgRNAs was confirmed by electrophoresis using a 10% TBE-urea precast gel (Bio-Rad).

The crRNAs and tracrRNA were resuspended in Nuclease-Free Duplex Buffer (IDT) to a final concentration of 100 µM. For complexing the RNAs, a 20 µM reaction was prepared by mixing 1 µl of each RNA with 3 µl of nuclease-free duplex buffer, incubating at 95˚C for 5 min, and leaving to cool to room temperature for at least 2 h before transfection.

Design of the single stranded oligonucleotides (ssODNs)

Approximately 125 bp asymmetric oligonucleotides were designed to contain the desired mutations and to have at least 36 nucleotides homologous to the genomic sequence 3' of the Cas9-induced double strand break (Table S3). The ssODNs were purchased as Ultramer DNA oligos (IDT) with standard desalting purification and resuspended in TE buffer to a final concentration of 100 µM.

CRISPR/Cas9 and ssODN transfection into hiPSCs

The gRNA, Cas9 protein (IDT or kindly provided by Niels Giessen (D'Astolfo et al., 2015)) and ssODN were transfected into the hiPSCs by either electroporation or lipofection. For electroporation, 1 µg Cas9 and 240 ng gRNA were mixed in a 0.5 ml sterile Protein LoBind tube (Eppendorf) and incubated at 25°C for 10 min to form the Cas9 ribonucleotide protein (RNP) complex. The hiPSCs were harvested and 1 x 10^5 cells mixed with the Cas9 RNP and 40 pmol ssODN in a 10 µl total reaction. Electroporation was performed using the Neon Transfection System (ThermoFisher), and the electroporated cells transferred immediately into a Laminin-521

coated well containing 500 µl StemFlex™ Medium with RevitaCell™ Supplement (1:100). Approximately 3 days later, the cells were harvested and expanded for subcloning.

For lipofection, MEF-maintained hiPSCs were transfected when 60–75% confluent. On the day of transfection, medium was refreshed 1 h before the procedure. Transfection reactions were prepared in a 0.5 ml sterile Protein LoBind tube by mixing 1.5 µg Cas9 with 380 ng of sgRNA, followed by the addition of Opti-MEM™ I Reduced Serum Medium (Gibco) to a final volume of 25 µl, and incubated as described above to form the Cas9 RNP complex. For co-delivery of the donor template, 4 pmol of ssODN was diluted in Opti-MEM™ I Reduced Serum Medium to a final volume of 25 µl. A lipofectamine mixture was prepared by diluting Lipofectamine® 2000 Transfection Reagent (Invitrogen) in Opti-MEM™ I Reduced Serum Medium (1:10). The Cas9 RNP, and ssODN solution were then combined with the diluted lipofectamine to a final volume of 100 µl and incubated at room temperature for 10 min before added to the hiPSCs. Approximately 6 h after transfection the media was refreshed, and at 72 h, the cells harvested and expanded for subcloning.

Targeting strategy to generate the KCNH2$^{PR/WT}$ hiPSC lines

Using the electroporation method, the KCNH2$^{PR/WT}$ hiPSC lines, LUMC0020iHERG-03 (KCNH2$^{PR/WT}$) and LUMC0020iHERG-04 (KCNH2$^{PR/WT}$ cl2), were created by introducing a heterozygous point mutation to substitute alanine to threonine at position 561 of KCNH2. A gRNA site 45 nucleotides 5' of the mutation site was targeted with a crRNA-tracrRNA complex, and the ssODN (ssODN_Pore) was designed to have a silent mutation within the PAM sequence to aide both colony screening and to prevent re-cutting of the modified locus by Cas9. For screening the resulting colonies, approximately 1 kB surrounding the target site was amplified by PCR and digested with *HaeII* to identify putative mono-allelic targeted clones. These were subsequently confirmed to be heterozygous for the mutation by Sanger sequencing.

Targeting strategy to generate the KCNH2$^{TL/WT}$ hiPSC lines

The KCNH2$^{TL/WT}$ hiPSC line (LUMC0020iHERG-01) was generated by introducing a heterozygous point mutation to substitute asparagine to isoleucine at position 996 of KCNH2. The target sequence for the sgRNA included the site to mutate, and the ssODN (ssODN Tail) was designed to include the desired nucleotide modification. For the KCNH2$^{TL/WT}$ cl2 hiPSC line (LUMC0020iHERG-02) a different sgRNA also covering the mutation site was transfected, along with both the ssODN Tail as well as a ssODN lacking the mutation but including two silent mutations. This additional ssODN functioned as competitor homology template to improve the frequency of obtaining a heterozygous clone. For screening the resulting colonies, approximately 1 kB surrounding the target site was amplified by PCR and digested with *BccI* to identify putative mono-allelic targeted clones. These were subsequently confirmed to be heterozygous for the mutation by Sanger sequencing.

Subcloning and PCR screening of the hiPSCs

Transfected hiPSCs were clonally isolated by single-cell deposition using a flow cytometer. Briefly, the cells were harvested using TrypLE™ Select Enzyme and filtered to remove cell clumps. If the transfected hiPSCs had been cultured on MEFs, prior to subcloning, the cells were stained with anti-MEF antibody (PE-conjugated; Miltenyi Biotec) to exclude these cells. All lines were stained with 4',6 Diamidino-2-Phenylindole (DAPI, Invitrogen) to exclude dead cells. A single hiPSC was deposited directly into each well of a 96-well plate in the same culture conditions as what the transfected hiPSCs had been maintained in. To assist with clonal recovery, the culture media also contained RevitaCell™ Supplement (1:100). Medium was initially changed three days after deposition and cells were maintained for ~2 weeks with medium changes every 3-4 days. Wells containing hiPSC colonies were dissociated with StemPro™ Accutase™ Cell Dissociation Reagent (Gibco) and duplicated across two 96-well plates. DNA for PCR screening and restriction fragment length polymorphism (RFLP) analysis was isolated from the cells in one plate with QuickExtract™ DNA Extraction Solution (Lucigen), while the cells in the replicate plate were cryopreserved.

Off-target analysis

Potential genomic off-targets for each gRNA were identified using http://crispor.tefor.net. The top 5 candidates with up to 4 base mismatches, were evaluated for the presence of Cas9-induced mutations (Table S2). The region around the off-target site was PCR amplified from both the targeted and KCNH2$^{WT/WT}$ hiPSC lines. The PCR amplicons were treated with Exonuclease I and Shrimp Alkaline Phosphatase (both New England Biolabs) and sequenced by Sanger sequencing to confirm the absence of off-target modifications.

Karyotyping

The genetically-modified hiPSC lines were karyotyped by G-banding. Chromosome spreads and analyses were performed by Cell Guidance Systems (UK) or by the Laboratory for Diagnostic Genome Analysis (Leiden University Medical Center). For each cell line, 20 metaphase spreads were examined with samples of sufficient quality to detect numerical and large structural abnormalities.

Allele-specific expression of *KCNH2*

Total RNA was isolated from hiPSC-CMs using the NucleoSpin® RNA Kit (Macherey-Nagel) according to manufacturer's instructions, with DNAse treatment performed using the DNA-free™ DNA Removal Kit (Ambion). RNA was reverse transcribed into cDNA using the iScript™ cDNA Synthesis Kit (Bio-Rad). Using primer pairs flanking each mutation (Table S4), PCR products generated from the *KCNH2* transcripts were cloned into the pMiniT™ 2.0 vector using the NEB® PCR Cloning Kit (New England Biolabs). For each *KCNH2* variant line at least 29 clones containing the transcript underwent Sanger sequencing.

Flow cytometric analysis

A single cell suspension of hiPSCs or hiPSC-CMs was obtained by dissociating the cells with TrypLE™ Select Enzyme and filtering the cell suspension. Cells were fixed and permeabilised using the Fix & Perm Cell Permeabilization Kit (Invitrogen) according manufacturer's instructions. For the hiPSC-CMs, the cells were first incubated with a Viobility™ 405/520 fixable dye (Miltenyi Biotec) prior to fixation for subsequent exclusion of dead cells. The hiPSCs were incubated with the conjugated antibodies OCT4-BV421 (1:25, BD #565644), Sox2-A488 (1:200, eBioscience, #53-9811-80), Tra-1-60-PE (1:20, Miltenyi Biotec, #130-100-347) and SSEA4-PE-vio770 (1:100, Miltenyi Biotec, #130-105-082), while the hiPSC-CMs were incubated with cTnT-Vioblue (1:11, Miltenyi Biotec, #130-106-686) and MLC2v-PE (1:11, Miltenyi Biotec, #130-106-183). All antibodies were diluted in permeabilization medium (medium B; Invitrogen). Samples were measured using a MACSQuant VYB flow cytometer (Miltenyi Biotec), and data analysed using FlowJo software (FlowJo).

Immunofluorescence analysis

For hiPSCs, cells were fixed in 2% paraformaldehyde for 30 min, permeabilized with phosphate buffer saline (PBS)/0.1% Triton X-100 (Sigma-Aldrich) and blocked with 1% bovine serum albumin (BSA, Sigma-Aldrich) and 0.05% Tween (Merck) in PBS. Samples were incubated overnight at 4°C with antibodies specific for NANOG (1:200, R&D, #AF1997) and SSEA4 (1:200, Santa Cruz Biotechnology, #SC59368). These primary antibodies were detected with Alexa Fluor 555- (1:500, ThermoFisher, #A21432) and Alexa Fluor 488- (1:500, Life Technologies, #A-21202) conjugated antibodies, respectively. Nuclei were visualised with DAPI (0.3 μM) and images captured using an EVOS FL Auto 2 Cell Imaging System (ThermoFisher).

The hiPSC-CMs plated on glass coverslips were fixed using the Inside Stain Kit (Miltenyi Biotec) according to manufacturer's instructions. The fixed cells were incubated with α-actinin (1:250, Sigma-Aldrich, #A7811) and myosin heavy chain (1:50, Miltenyi Biotec, #130-112-757) antibodies, followed by Alexa Fluor 594- (1:250, ThermoFisher, #A-21203) and Vio515- (1:100, Miltenyi Biotec, #130-112-760) conjugated secondary antibodies. Nuclei were stained with DAPI and images captured using a confocal laser scanning microscope SP8 (Leica) at 40x magnification.

Western Blot

Protein isolation and western blot in hiPSC-CMs were performed as previously described (Bellin et al., 2013). Briefly, samples were lysed in RIPA Lysis and Extraction Buffer and total protein measured using the Pierce™ BCA Protein Assay (both Thermo Fisher) according to manufacturer's protocols. The protein samples (40 μg) were loaded on an 8% polyacrylamide gel, and transfer performed using standard protocols (Kurien and Scofield, 2006). The membrane was incubated with the primary antibodies hERG1a (1:1000, Cell Signalling Technology, #12889) and β-actin (1:2000, Abcam, #8227 or 1:2000, Merck, #MAB1501R), followed by appropriate HRP-conjugated secondary antibodies (1:10000, Cell Signalling Technology, #7074 and #7076S), and the chemiluminescence signal detected using WesternBright Quantum HRP substrate (Isogen Life Science).

Patch clamp data acquisition

Electrophysiological recordings were performed on single hiPSC-CMs. For action potential (AP) and rapid delayed rectifier potassium current (I_{Kr}) measurements, spontaneously contracting cardiomyocytes were selected in Tyrode's solution containing (in mM): NaCl 140, KCl 5.4, $CaCl_2$ 1.8, $MgCl_2$ 1.0, glucose 5.5, and HEPES 5.0; pH 7.4 (NaOH). I_{Kr} and APs were measured with the ruptured or perforated patch-clamp technique, respectively, using an Axopatch 200B amplifier (Molecular Devices) at 36 ± 0.2°C. Voltage control and data acquisition of I_{Kr} and APs were performed with pClamp 10.4/Clampfit (Axon Instruments) and custom-made software, respectively. Potentials were corrected for the calculated liquid junction potentials (Barry and Lynch, 1991), which was 15 mV for both AP and I_{Kr} measurements. Low-resistance patch pipettes (2-3 MΩ; Borosilicate glass capillaries, Harvard Apparatus) were used, and for I_{Kr} measurements, series resistance (Rs) was compensated for ≥80%. I_{Kr} and APs were low-pass filtered (cut-off frequency of 5 kHz and 2 kHz, respectively), and digitised (55 kHz and 40 kHz, respectively).

Voltage-clamp experiments
I_{Kr} was measured using 4 s hyper- and depolarising pulses from a holding potential of -40 mV, at a cycle length of 10 s. The extracellular solution was Tyrode's solution, while the pipette solution contained (in mM): K-gluconate 125, KCl 20, K_2-ATP 5, HEPES 10, and EGTA 10; pH 7.2 (KOH). The L-type Ca^{2+} current was blocked by adding 5 µM nifedipine (Sigma) to the extracellular solution. I_{Kr} was measured as an E-4031-sensitive current by subtracting the current recorded before and after application of 5 µM E-4031 (Tocris). I_{Kr} densities (steady-state and tail) were calculated by dividing current amplitude (pA), measured at the depolarizing voltage steps and upon stepping back to the holding potential, by cell membrane capacitance (pF). Cell membrane capacitance was measured by dividing the decay time constant of the capacitive transient in response to 5 mV hyperpolarising steps from -40 mV, by the Rs.

Current-clamp experiments
APs were measured in Tyrode's solution, while the pipette solution contained (in mM): K-gluconate 125; KCl 20; NaCl 5.0; amphotericin-B 0.44, and HEPES 10; pH 7.2 (KOH). Because hiPSC-CMs typically have a small or even absent inward rectifying potassium current (I_{K1}), the cells have a depolarised resting membrane potential (RMP) and are frequently spontaneously active. We therefore injected an *in silico* 2 pA/pF I_{K1} with kinetics of $Kir_{2.1}$ channels through dynamic clamp, resulting in quiescent hiPSC-CMs with a RMP of less than -75 mV. APs were elicited at 0.2 Hz, 0.5 Hz, 1 Hz, 2 Hz, 3 Hz and 4 Hz by 3 ms, ~1.2x threshold current pulses through the patch pipette. The RMP, upstroke velocity, AP amplitude, AP plateau amplitude and AP duration at 20%, 50% and 90% repolarisation were analysed. Parameters from 13 consecutive APs were averaged.

MEA electrophysiology
Multi-electrode array (MEA) experiments to record spontaneous electrical activity of hiPSC-CMs were done using 60-electrode MEA chips or multi-well MEA plates (both Multichannel Systems). The MEAs were coated with human fibronectin (40 µg/ml, Alfa Aesar) for 1 h at 37°C, and hiPSC-CMs seeded directly on the electrodes at a density of 1.5×10^5 cells/cm^2. Medium was changed at least 1 h before baseline recordings. All measurements were made at 37°C in Medium C and at least 15 min after placing the MEA in the recording system. Extracellular recordings for the 60-electrode MEA chips were performed using a MEA1060INV amplifier (Multichannel systems) as previously described (Sala et al., 2017). Recordings for the multi-well MEA plates were made using a Multi-well-MEA system (Multichannel systems) at a sampling rate of 20 kHz and high- and low pass filters of 3500 Hz and 1 Hz respectively.

For evaluating E-4031-induced effects on the hiPSC-CMs, sequential addition of increasing concentrations of E-4031 was performed. E-4031 (Tocris) was dissolved in DMSO (Sigma-Aldrich) at 10 mM, with serial dilutions made in Medium C. The final concentration of the drug was achieved by stepwise removal of medium and addition of the same volume of diluted E-4031 to the well. No more than 7% of the total volume was replaced. The response to each E-4031 concentration was recorded for 1 min after an incubation period of 1 min. The maximum amount of DMSO present in the culture medium was 0.3% at the highest concentration of E-4031. Controls indicated that this percentage of DMSO did not alter the field potential duration (FPD) of the hiPSC-CMs or trigger arrhythmic events.

MEA traces were analysed with the investigator blinded, with FPD and peak-to-peak intervals quantified over the whole recording time as previously described (Sala et al., 2017). Good quality traces were defined by the presence of a clear peak corresponding to the Na^+ influx and membrane depolarization, a clear repolarization phase corresponding to K^+ efflux, and a high signal-to-noise ratio (SNR). The SNR was very high for the vast majority of recordings. However if the trace was noisy, filtering was performed prior to analysis. Fridericia's formula for frequency correction was used to correct for FPD dependence on beating rate (cFPD)

$$\frac{FPD}{\sqrt[3]{RR}}$$

where FPD is in ms and RR in s. Compound responses of the hiPSC-CMs were normalised to their baseline measurement. Arrhythmia-like events were classified and scored based on the following categories: no arrhythmia (0); variable beat period (1); abnormal depolarisations (2); fibrillation (3); quiescent (4). Fibrillation was defined as a severe reduction in the FP amplitude, leading to an almost complete loss the Na^+ peak, concomitant with the presence of uncoordinated electrical activity. Quiescent cells were counted as arrhythmic cells with the highest score based on the I_{Kr}-blocking effects of E-4031. Increasing amounts of E-4031 prolonged the FPD which resulted at high concentrations in a failure of membrane potential to properly repolarise, and consequently an inability to evoke APs at such depolarised diastolic potentials.

For evaluating lumacaftor-induced effects on hiPSC-CMs, the cells were seeded at a density of 4.4 x 10^5 cells/cm^2 and cultured on 96-well MEA plates (M768-tMEA-96OPT, Axion Biosystems) as described above. 7 days after seeding, the hiPSC-CMs were treated with 5 µM (final concentration) lumacaftor (VX-809; MedChemExpress) or vehicle control (final concentration 0.1% DMSO) for 8 days with the compound refreshed every 48 hrs. Extracellular recordings were performed using a Maestro Pro system (Axion Biosystems) before addition of the compounds and after 8 days of treatment. MEA traces were analysed using the platform-based Cardiac Analysis Tool to determine the FPD and beat period. Frequency correction was not used for lumacaftor responses since this overestimated the rate-dependency of the FPD. Compound responses of the hiPSC-CMs were normalised to their baseline measurement.

Optical evaluation using the Triple Transient Measurement (TTM) system

The hiPSC-CMs were plated at between 3 – 5.5 x 10^4 cells per well in black glass-bottom 96-well plates (Greiner), pre-coated with 1:100 Matrigel in DMEM/F-12 (Gibco). Medium was replaced the next day and thereafter every 2-3 days with Pluricyte Cardiomyocyte Medium (PCM; NCardia). Analysis was performed 5-7 days after plating. Cells were incubated for 20 min at 37°C (protected from light) with a voltage sensitive dye (6 µM ANNINE-6plus; Sensitive Farbstoffe), a Ca^{2+} sensitive dye (6 µM Rhod 3; ThermoFisher) and a cell membrane labelling dye (5 µM CellMask Deep Red; ThermoFisher) in PCM for measuring electrical activity, Ca^{2+} flux and contraction, respectively. The dye medium was then removed and PCM added, with cells left to recover for 10-15 min at 37°C protected from light before analysis.

Recording and data processing were performed using a bespoke fast optical switch microscopy system and algorithms developed in-house (van Meer et al., 2019). Briefly, cells were paced at 1.2 Hz using 10 ms pulses of 14 V with a pair of field stimulation electrodes placed in the culture medium. An Eclipse Ti microscope (Nikon) was fitted with a high-power three LED system (Mightex; 470 nm, 560 nm and 656 nm) including collimators and excitation filters (Semrock; 470±14 nm, 544±12 nm, 650±7.5 nm). A 63x oil immersion objective was used, together with an image intensifier (Photonis) and a high-speed camera (Optronis). Plates were maintained in an environmental chamber at 5% CO_2 and 37°C. Three fields of view were measured per well, with recordings of all three fluorescence channels performed at 1000 frames/s for 7 s using fast optical switching of LEDs (1 ms per channel). The three channel signals were then separated and peaks averaged. The 90% durations were then calculated. For voltage this was taken as the top of the peak to 90% of repolarisation, whereas for Ca^{2+} and contraction this was taken from 10% above baseline in the upstroke to 90% of the decay. Five wells were analysed per differentiation of hiPSC-CMs, with three independent differentiations used to generate the mean data per line (n = 15 wells, 45 fields of view).

References

Barry, P.H., and Lynch, J.W. (1991). Liquid junction potentials and small cell effects in patch-clamp analysis. J. Membr. Biol. *121*, 101–117.

Bellin, M., Casini, S., Davis, R.P., D'Aniello, C., Haas, J., Ward-van Oostwaard, D., Tertoolen, L.G.J., Jung, C.B., Elliott, D.A., Welling, A., et al. (2013). Isogenic human pluripotent stem cell pairs reveal the role of a KCNH2 mutation in long-QT syndrome. EMBO J. *32*, 3161–3175.

van den Berg, C.W., Elliott, D.A., Braam, S.R., Mummery, C.L., and Davis, R.P. (2014). Differentiation of Human Pluripotent Stem Cells to Cardiomyocytes Under Defined Conditions. Methods Mol. Biol. *1353*, 163–180.

D'Astolfo, D.S., Pagliero, R.J., Pras, A., Karthaus, W.R., Clevers, H., Prasad, V., Lebbink, R.J., Rehmann, H., and Geijsen, N. (2015). Efficient Intracellular Delivery of Native Proteins. Cell *161*, 674–690.

Davis, R.P., Grandela, C., Sourris, K., Hatzistavrou, T., Dottori, M., Elefanty, A.G., Stanley, E.G., and Costa, M. (2009). Generation of Human Embryonic Stem Cell Reporter Knock-In Lines by Homologous Recombination. Curr Protoc Stem Cell Biol Chap.5 Unit 5B 1 1 1-34.

Kurien, B., and Scofield, R. (2006). Western blotting. Methods *38*, 283–293.

van Meer, B.J., Krotenberg, A., Sala, L., Davis, R.P., Eschenhagen, T., Denning, C., Tertoolen, L.G.J., and Mummery, C.L. (2019). Simultaneous measurement of excitation-contraction coupling parameters identifies mechanisms underlying contractile responses of hiPSC-derived cardiomyocytes. Nat. Commun. *10,* 4325.

Pua, C.J., Bhalshankar, J., Miao, K., Walsh, R., John, S., Lim, S.Q., Chow, K., Buchan, R., Soh, B.Y., Lio, P.M., et al. (2016). Development of a Comprehensive Sequencing Assay for Inherited Cardiac Condition Genes. J. Cardiovasc. Transl. Res. *9*, 3–11.

Sala, L., Ward-van Oostwaard, D., Tertoolen, L.G.J., Mummery, C.L., and Bellin, M. (2017). Electrophysiological Analysis of human Pluripotent Stem Cell-derived Cardiomyocytes (hPSC-CMs) Using Multi-electrode Arrays (MEAs). J. Vis. Exp. *123,* e55587.
